# Supplementary figures and images for: Structure of a bacterial Rhs effector exported by the type VI secretion system
Source: PLoS Pathog. 2022 Jan 5;18(1):e1010182. doi: 10.1371/journal.ppat.1010182 (PMC8765631; doi:10.1371/journal.ppat.1010182)

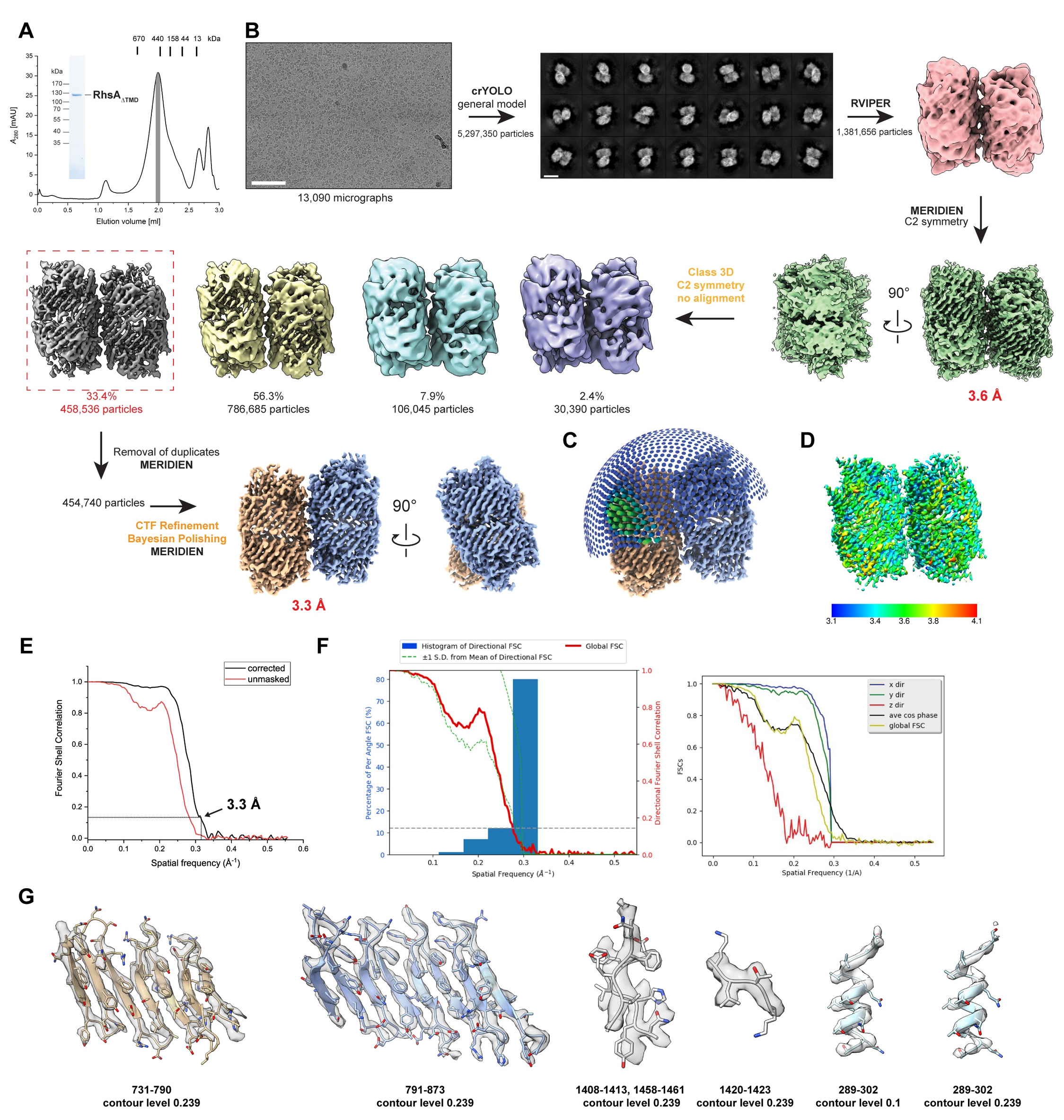

Supplement: S1 Fig — (A) Purification of RhsAΔTMD via size-exclusion chromatography using a Superose 6 5/150 increase column. Molecular weight standards are indicated at their respective elution volumes. The grey bar denotes pooled and concentrated fractions used for cryo-EM analysis. The same material was analyzed for purity via semi-denaturing SDS-PAGE imaged with a stain-free filter. (B) Representative cryo-EM micrograph of RhsAΔTMD used for structural determination. Scale bar EM micrograph, 100 nm. Particles were picked with the general model of crYOLO. The rest of the processing workflow is indicated and summarized in the Materials and Methods section. Scale bar 2D class averages, 10 nm. Used software packages are highlighted. Orange font depict steps carried out in Relion. The final map was calculated using MERIDIEN and postprocessed with DeepEMhancer. (C) Angular distribution plot of the final reconstruction. (D) Local resolution estimates visualized on a map postprocessed in SPHIRE. (E) Fourier shell correlation plot calculated from two independently processed maps. Resolution estimation is reported at the gold standard cutoff of 0.143. (F) Resolution anisotropy was assessed with the 3DFSC online server tool. (G) Selected regions of the map are shown as transparent surface with the built atomic models as stick representations. (TIF) [file ppat.1010182.s001.tif]

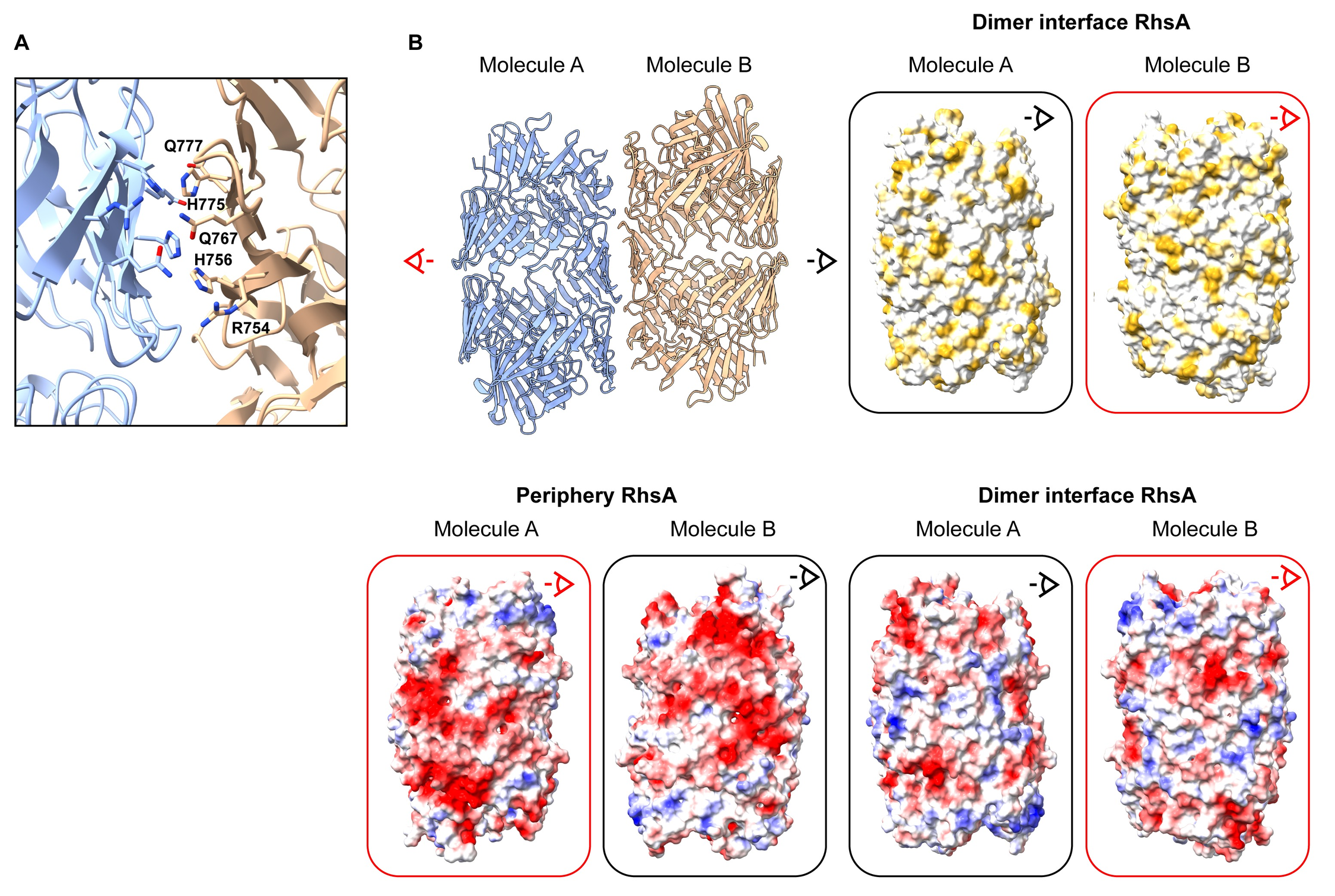

Supplement: S2 Fig — (A) Close up view of the dimer interface of the two RhsA molecules at the central symmetry axis. Potential candidate residues engaging in stabilizing interactions are labelled and shown in stick representation. (B) Surface properties of the dimer interface and the periphery of RhsA. The surface of the interface is colored according to its Coulomb potential indicating positively (red, -20) and negatively (blue, +20) charged areas. The second representation shows the same interface but colored according to hydrophobicity. Ochre indicates hydrophobic and white indicates hydrophilic regions. The area facing the outer peripheries of both barrels shows that these regions would electrostatically repel each other and explain why only two barrels can interact at the same time. (TIF) [file ppat.1010182.s002.tif]

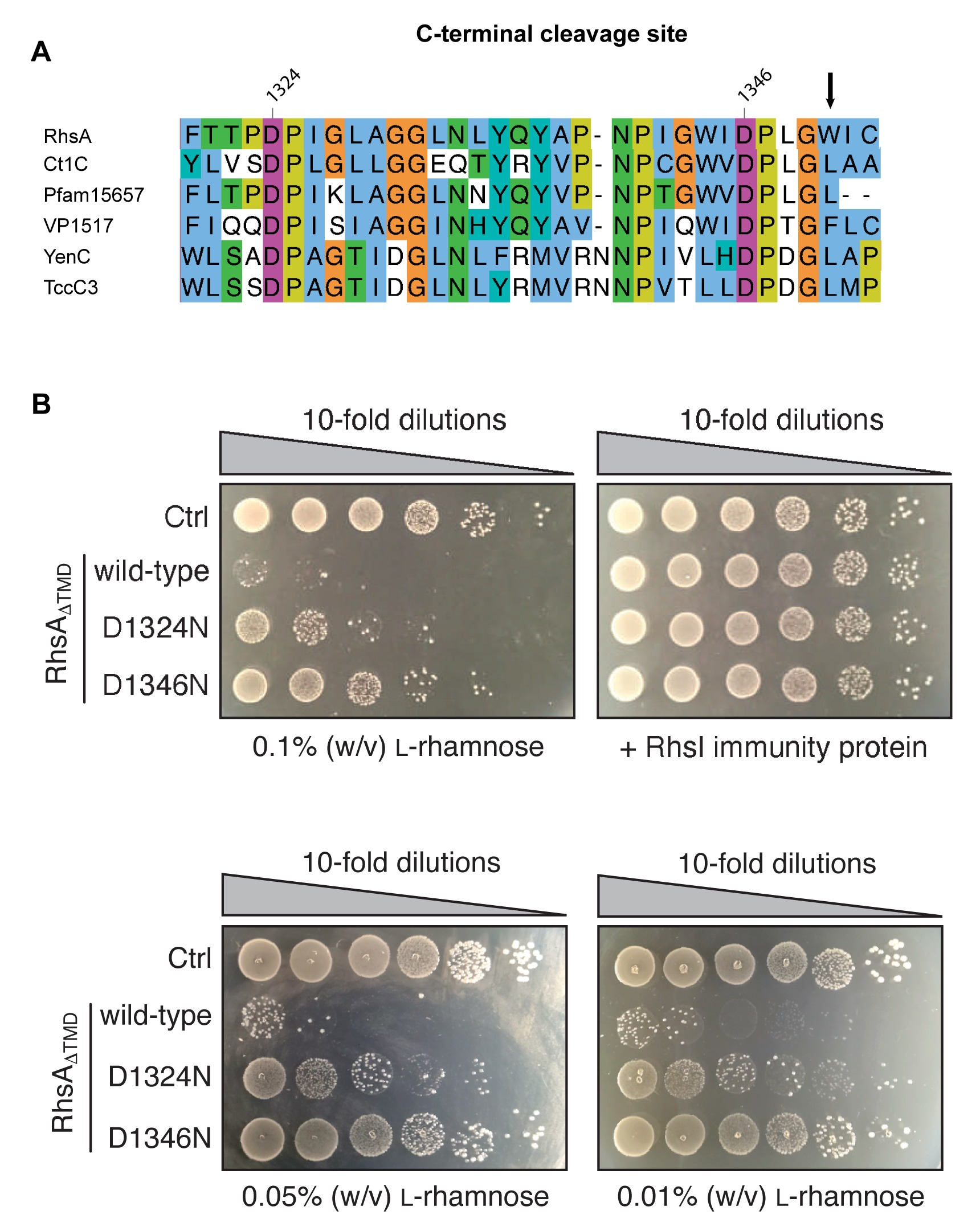

Supplement: S3 Fig — (A) Multiple sequence alignment of the C-terminal cleavage site. The cleavage site is indicated by the black arrow. Critical residues are highlighted by the residue numbering. Coloring is according to the ClustalW color code. (B) In E. coli toxicity assays show reduced toxicity of RhsAΔTMD harboring mutations of the catalytic aspartates D1324N and D1346N, respectively. Toxicity could by reversed by overexpression of the immune protein RhsI. The lower panel represents the same toxicity assay but with lower RhsA expression levels due to lower inducer concentration (L-rhamnose). (TIF) [file ppat.1010182.s003.tif]

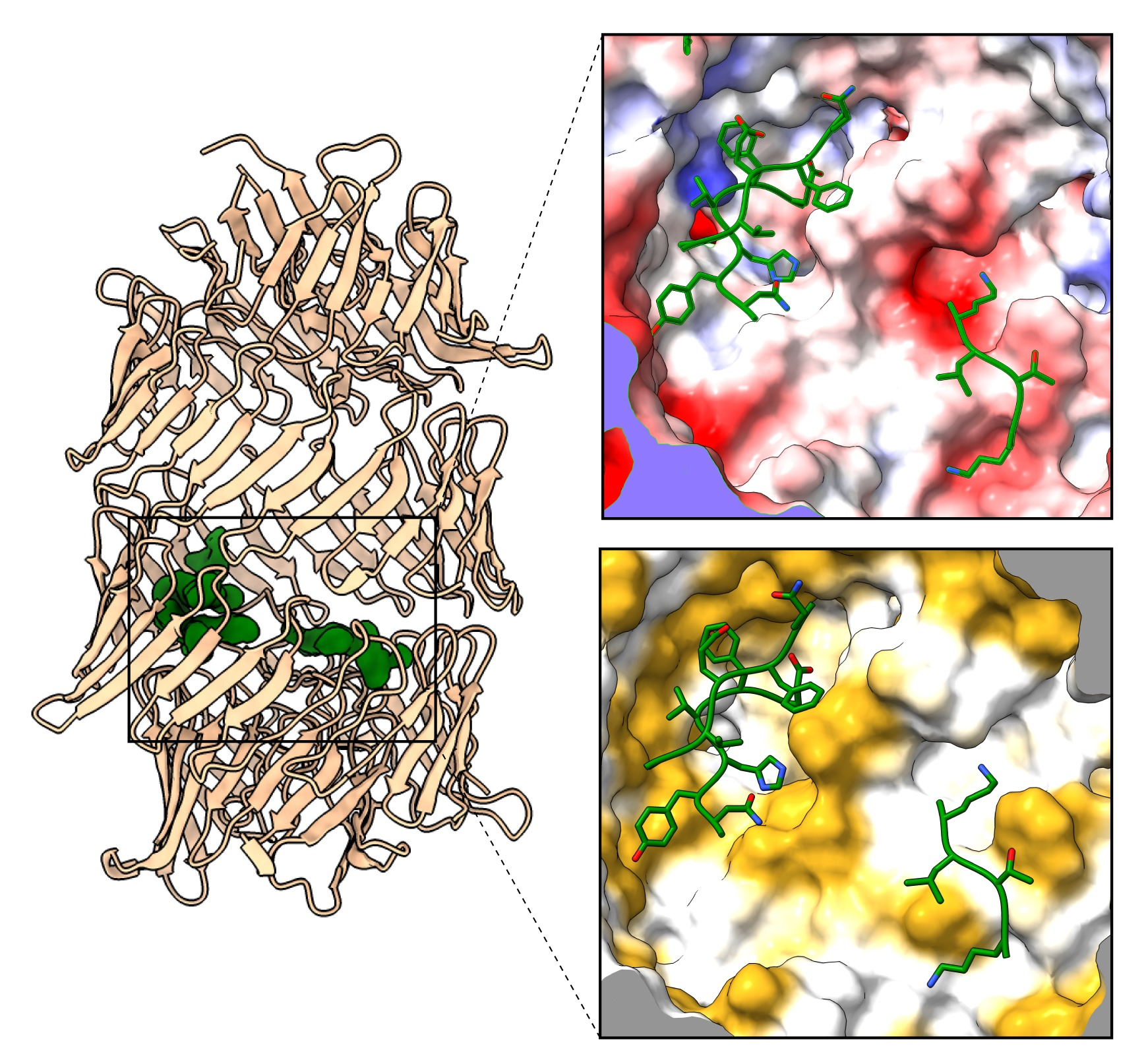

Supplement: S4 Fig — Interaction of three β-strands belonging to the toxin domain of RhsA with the Rhs barrel. The toxin fragments are stabilized by the interaction with hydrophobic and hydrophilic surfaces inside the Rhs barrel. The molecular surface is colored according to hydrophobicity with ochre and white indicating hydrophobic and hydrophilic regions, respectively. The electrostatic representation is colored according to Coulomb potential, which depicts positively (red, -20) and negatively (blue, +20) charged areas. (TIF) [file ppat.1010182.s004.tif]

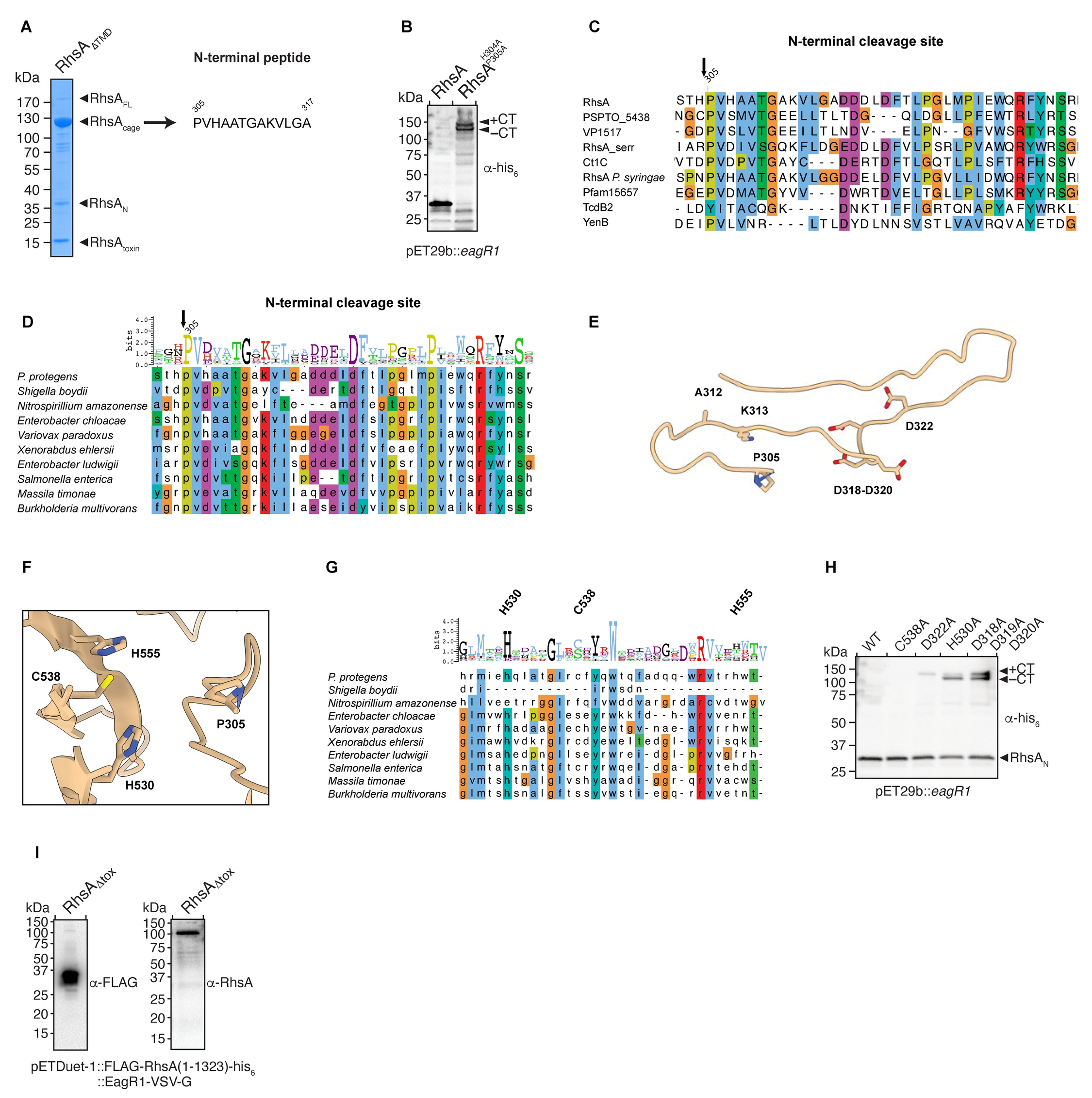

Supplement: S5 Fig — (A) The N-terminal cleavage site was identified by protein sequencing. The band containing the Rhscage was excised and analyzed by de novo protein sequencing using LC-MS/MS. (B) Western blot analysis of wild-type or a N-terminal cleavage resistant mutant (H304A/P305A) confirming the location of the cleavage site at P305. (C) Multiple sequence alignment of the N-terminal cleavage site. The cleavage site is indicated by a black arrow. Coloring is according to the ClustalW color code. (D) Sequence alignment of the T6SS class I prePAAR effectors highlighting the N-terminal cleavage site. Residue conservation is depicted as Weblogo. The Weblogo represents residue conservation. (E) Cartoon representation of the N-terminal cleavage site in RhsA. Residues A312 and K313 as well as conserved and potential catalytically active residues are show in stick representation. Residues A312 and K313 correspond to the catalytically active glutamates in TseI [18]. (F) Hypothesized cysteine protease motif which is near the N-terminal cleavage site P305. (G) Multiple sequence alignment highlighting the conservation of the hypothesized cysteine protease motif among class I prePAAR effectors. The Weblogo represents residue conservation. (H) Western blot analysis of potential residues involved in N-terminal cleavage and generation of the cleavage product RhsAN. Impaired cleavage was assessed by appearance of the full-length RhsA chain which is a mixture of both species, the C-terminally cleaved fragment (-CT) and the C-terminally uncleaved fragment (+CT). RhsA was coexpressed with its cognate chaperone EagR1. The blot was performed against N-terminal His6-tagged proteins (α-His). (I) Western blot analysis to examine the potential involvement of RhsA’s C-terminal toxin domain in the autoproteolysis of its N-terminal region. An RhsA truncation lacking the C-terminal toxin domain (RhsAΔtox, residues 1–1323) was expressed and examined for N-terminal cleavage using antibodies that recognize its N [file ppat.1010182.s005.tif]

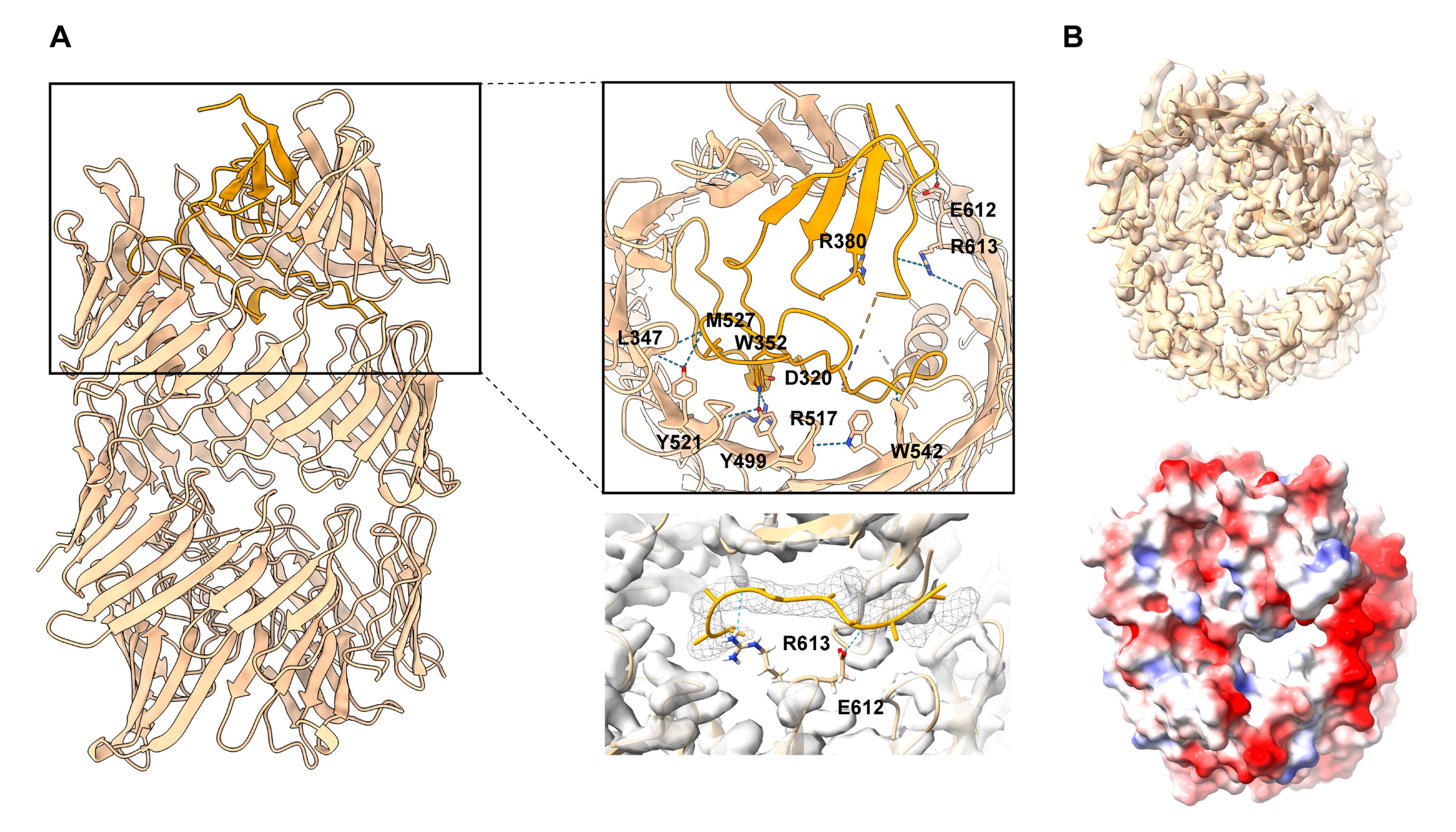

Supplement: S6 Fig — (A) Only a few hydrophilic interactions stabilize the plug domain. Residues participating in hydrogen bonds are labeled and shown in stick representation. The cork and the seal are colored in orange whereas the Rhs repeats is colored in beige. (B) The barrel would not be closed without the observed density corresponding to the seal. The model for the seal was manually removed to visualize the opening though which toxin is threaded into the target cell after removal of the seal and the anchor helix. (TIF) [file ppat.1010182.s006.tif]

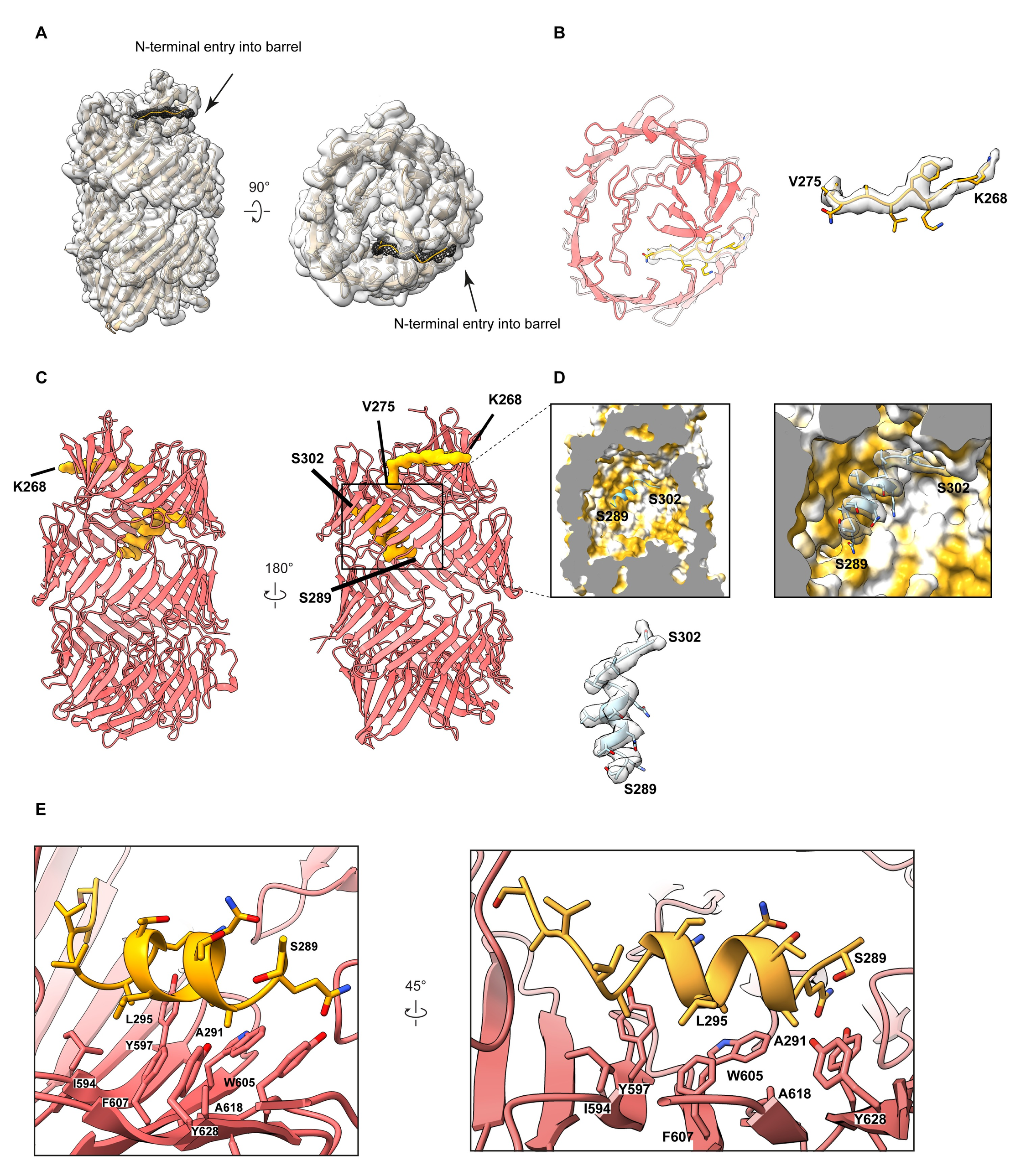

Supplement: S7 Fig — (A-B) A small density, corresponding to seal (residues K268-V275) of RhsA enters the barrel from the top (mesh). This results in a complete sealing of the cocoon. (C–E) The seal leads further down into an amphipathic helix which strongly interacts with the inner surface of the cocoon and thus serves as an anchor point for the N-terminal domain. The amphipathic helix is stabilized by hydrophobic interactions with Rhs repeats. (TIF) [file ppat.1010182.s007.tif]

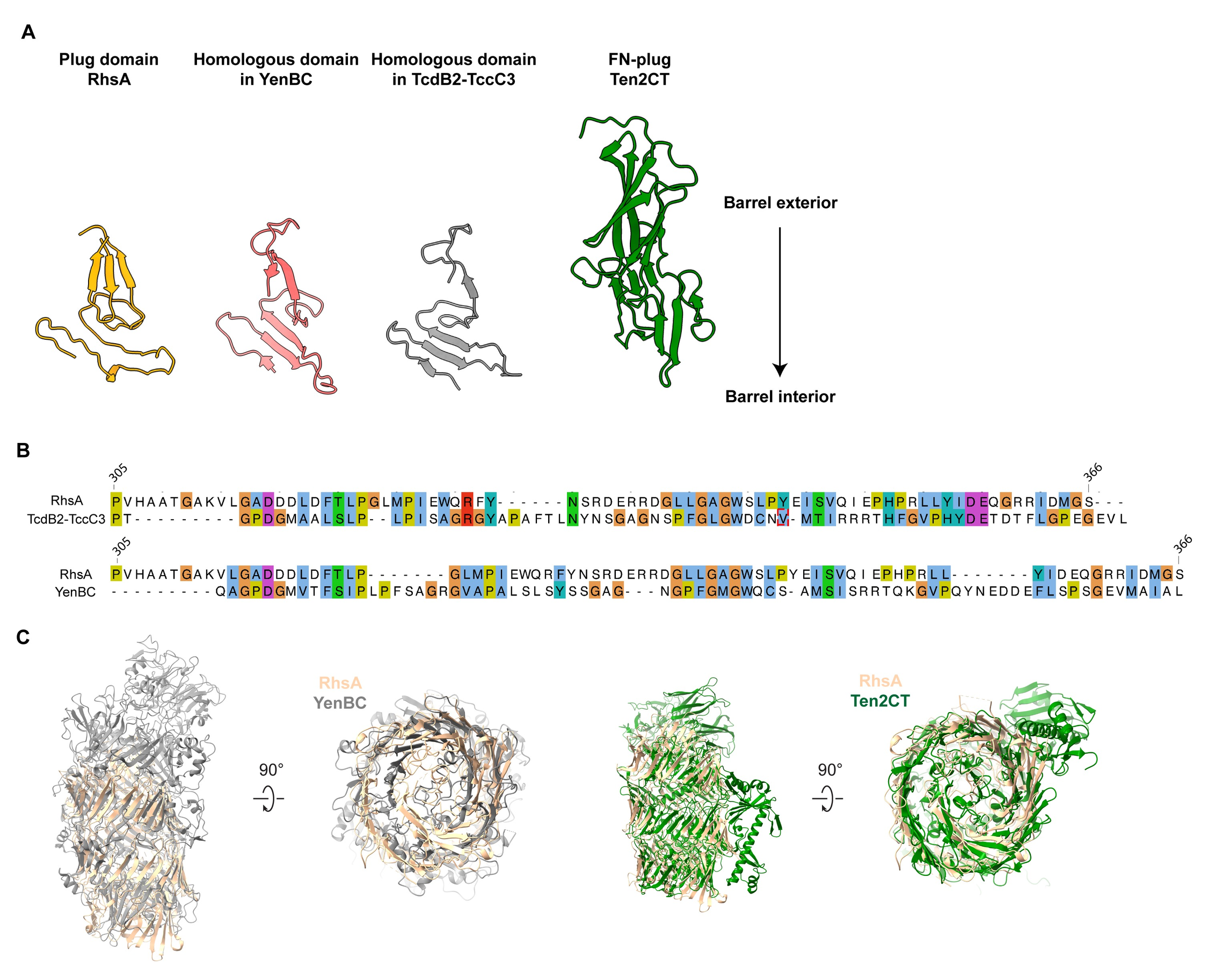

Supplement: S8 Fig — (A) Comparison of the plug domains of RhsA (orange) with YenBC (PDB ID: 4IGL), TcdB2-TccC3 (PDB ID: 6H6G) and Ten2CT (PDB ID: 6FB3). (B) Sequence alignment of the plug domain of RhsA with the sequences of the homologous domains found in TcdB2-TccC3 (top) and YenBC (bottom) are shown. Residues are colored according to the ClustalW color code. (C) Structural overlay of RhsA with YenBC (left) and Ten2CT (right). (TIF) [file ppat.1010182.s008.tif]

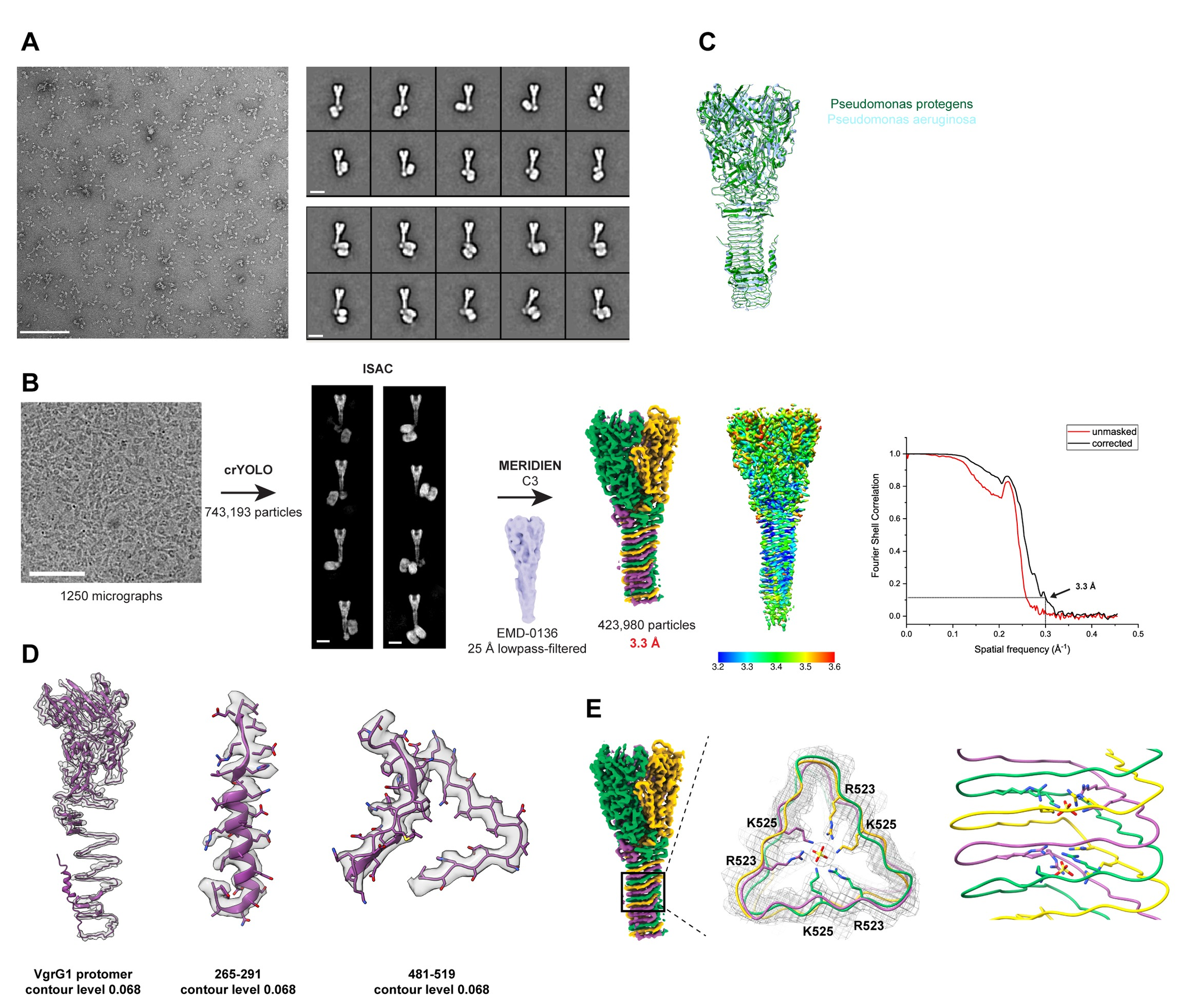

Supplement: S9 Fig — (A) Representative negative stain micrograph and 2D class averages of the intact PFC consisting of VgrG1, RhsA and EagR1. Scale bar micrograph, 100 nm. Scale bar 2D class averages, 10 nm. (B) Cryo-EM micrograph of the PFC and image processing workflow. Particles were picked with the general model of crYOLO and classified with ISAC. Refinement in MERIDIEN led to a reconstruction of 3.3 Å (FSC = 0.143 criterion). The final reconstruction was either colored individually for each protomer or according to local resolution. Fourier shell correlation was plotted according to two independent refined maps. Dashed line indicates the gold standard FSC criterion of 0.143. Scale bar micrograph, 100 nm. Scale bar 2D class averages, 10 nm. (C) Overlay of VgrG1 from P. protegens (green) with VgrG1 from P. aeruginosa (blue, PDB ID: 6H3N). (D) Map quality of selected parts of the structure (transparent surface) with built atomic models (cartoon representation). (E) Potential ion binding site in the middle part of the β-prism of P. protegens VgrG1. Sulfate ions were modelled into the spherical densities. Coordinating residues are displayed in stick representation. (TIF) [file ppat.1010182.s009.tif]

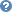

Supplement: S1 Dataset — (ZIP) [file ppat.1010182.s013.zip › S1_file/img/q.png]

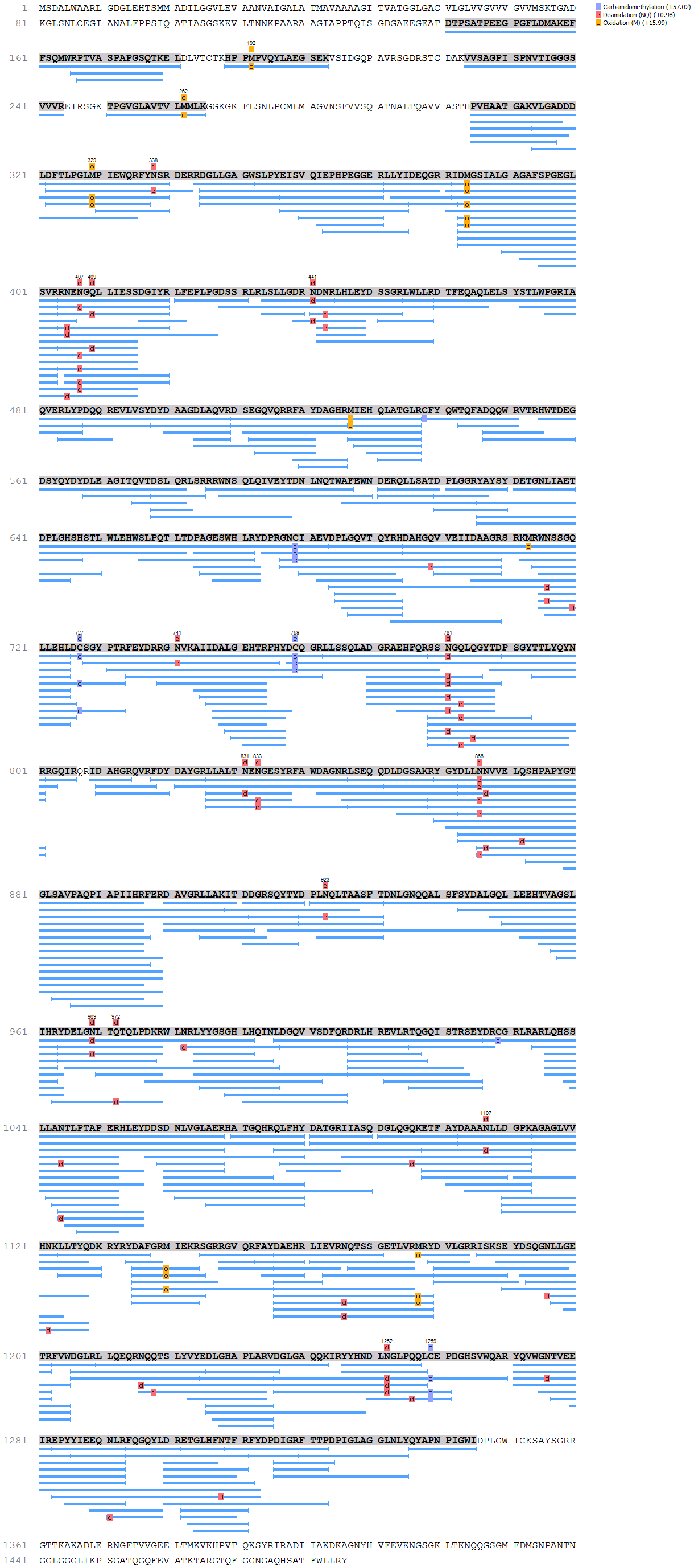

Supplement: S1 Dataset — (ZIP) [file ppat.1010182.s013.zip › S1_file/img/cov_2.png]

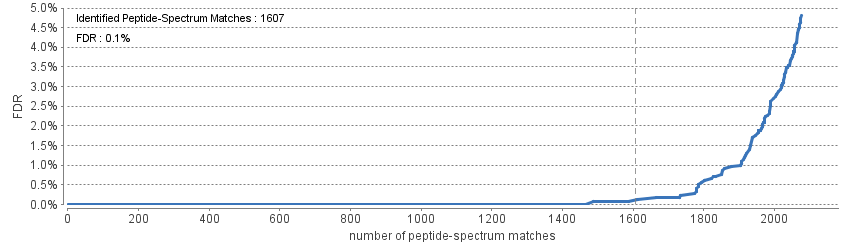

Supplement: S1 Dataset — (ZIP) [file ppat.1010182.s013.zip › S1_file/img/FDRFigure3131743580031004604.png]

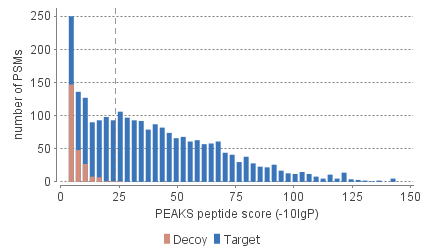

Supplement: S1 Dataset — (ZIP) [file ppat.1010182.s013.zip › S1_file/img/ScoreHistogram4629262077623001452.png]

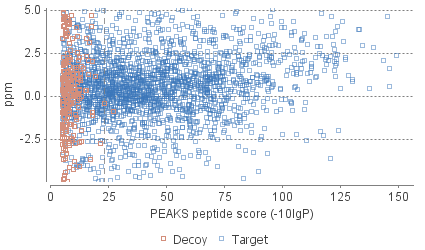

Supplement: S1 Dataset — (ZIP) [file ppat.1010182.s013.zip › S1_file/img/ScorePlotFigure8860971637774178472.png]

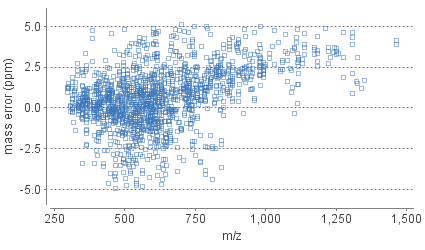

Supplement: S1 Dataset — (ZIP) [file ppat.1010182.s013.zip › S1_file/img/ErrorPlotFigure7158942499804334216.png]

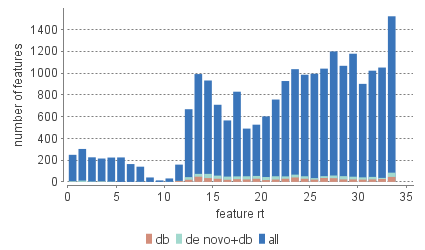

Supplement: S1 Dataset — (ZIP) [file ppat.1010182.s013.zip › S1_file/img/FeatureRtHistogram7180665927518386389.png]

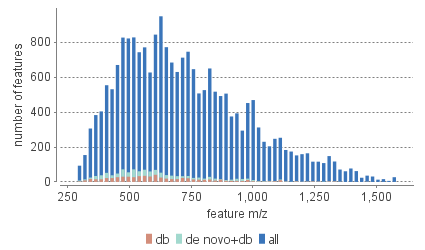

Supplement: S1 Dataset — (ZIP) [file ppat.1010182.s013.zip › S1_file/img/FeatureMzHistogram8370668363565372825.png]

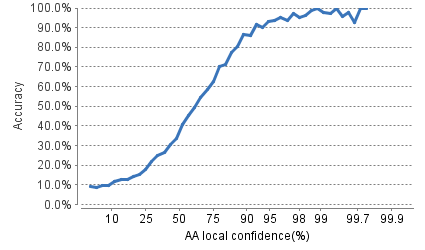

Supplement: S1 Dataset — (ZIP) [file ppat.1010182.s013.zip › S1_file/img/DenovoFDRCurveFigure871652953567853351.png]

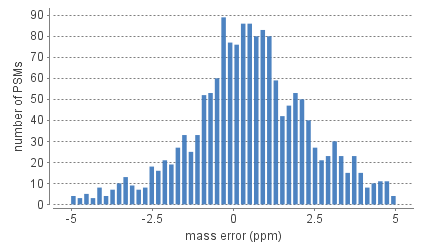

Supplement: S1 Dataset — (ZIP) [file ppat.1010182.s013.zip › S1_file/img/ErrorCalibratedHistogram9122836498367187363.png]

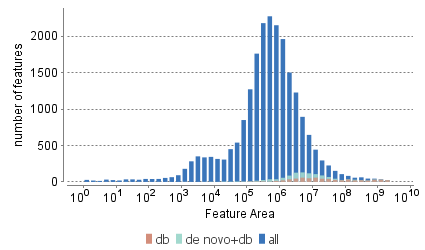

Supplement: S1 Dataset — (ZIP) [file ppat.1010182.s013.zip › S1_file/img/FeatureIntensityDistributionHistogram5853475060124105132.png]
