# Supplementary material for: Structure of a bacterial Rhs effector exported by the type VI secretion system
Source: PLoS Pathog. 2022 Jan 5;18(1):e1010182. doi: 10.1371/journal.ppat.1010182 (PMC8765631; doi:10.1371/journal.ppat.1010182)
Supplement: S1 Table — (DOCX) [file ppat.1010182.s010.docx]

| Organism | Genotype | Description | Reference |
| --- | --- | --- | --- |
| *P. protegens* Pf-5 | wild-type |  | Paulsen et al., Nature Biotechnology, 2005[1] |
|  | PFL_6096_D1324N | Expresses RhsA D1324N | This study |
|  | PFL_6096_D1346N | Expresses RhsA D1346N | This study |
|  | ΔPFL_6079 attB::*lacZ*, TetR | *pppA* deletion, constitutive *lacZ* expression, Tet^R^ | Ahmad et al., eLife, 2020[2] |
|  | ΔPFL_6079 ΔPFL_6096 ΔPFL_6097 attB::lacZ, TetR | *pppA* *rhsA* *rhsI* deletion, constitutive *lacZ* expression, Tet^R^ | Ahmad et al., eLife, 2020[2] |
| *E. coli* SM10 λpir | *thi thr leu tonA lac Y supE recA*::RP4-2-Tc::Mu | Conjugation strain | BioMedal LifeScience |
| *E. coli* XL-1 Blue | *recA1* *endA1* *gyrA96 thi-1 hsdR17 supE44 relA1 lac* [F´ *proAB lacI*^q^ Z∆*M15* Tn*10* (Tet^R^)] | Cloning strain | Novagen |
| *E. coli* BL21 (DE3) CodonPlus | F^-^ ompT gal dcm lon hsdS_B_(r_B_^-^ m_B_^-^) λ(DE3) pLysS(cm^R^) | Protein expression strain | Novagen |

**References**

1. Paulsen IT, Press CM, Ravel J, Kobayashi DY, Myers GSA, Mavrodi D V., et al. Complete genome sequence of the plant commensal Pseudomonas fluorescens Pf-5. Nat Biotechnol. 2005;23: 873–878. doi:10.1038/nbt1110

2. Ahmad S, Tsang KK, Sachar K, Quentin D, Tashin TM, Bullen NP, et al. Structural basis for effector transmembrane domain recognition by type VI secretion system chaperones. Elife. 2020;9: 1–29. doi:10.7554/eLife.62816
