# Supplementary material for: Structure of a bacterial Rhs effector exported by the type VI secretion system
Source: PLoS Pathog. 2022 Jan 5;18(1):e1010182. doi: 10.1371/journal.ppat.1010182 (PMC8765631; doi:10.1371/journal.ppat.1010182)
Supplement: S2 Table — (DOCX) [file ppat.1010182.s011.docx]

| Plasmid | Relevant features | Reference |
| --- | --- | --- |
| pETDuet-1 | Co-expression vector with *lacI*, T7 promoter, N-terminal His_6_ tag in MCS-1, Amp^R^ | Novagen |
| pET29b | Expression vector with *lacI*, T7 promoter, C-terminal His_6_ tag, Kan^R^ | Novagen |
| pEXG2 | Allelic exchange vector containing *sacB*, Gm^R^ | Hmelo et al., Nature Protocols, 2015[1] |
| pSCrhaB2-CV | Expression vector with *PrhaB*, Tmp^R^ | Cardona et al., Plasmid, 2005[2] |
| pPSV39-CV | Expression vector with *lacI*, *lacUV5* promoter, C-terminal VSV-G tag, Gm^R^ | Silverman, Cell, 2013[3] |
| pETDuet-1::PFL_6096 ::PFL_6097 | Co-expression vector for RhsA and RhsI | Ahmad et al., eLife, 2020 |
| pETDuet-1::PFL_6096_74-CT ::PFL_6097 | Co-expression vector for RhsA_ΔNT_ and RhsI | Ahmad et al., eLife, 2020[4] |
| pET29b::PFL_6094 | Expression vector for VgrG1 | This study |
| pETDuet-1::PFL_6096_74-CT_VSV-G ::PFL_6097 | Co-expression vector for C-terminally VSV-G tagged RhsA_ΔNT_ and RhsI | This study |
| pETDuet-1::PFL_6096_74-CT_D1324N_VSV-G ::PFL_6097 | Co-expression vector for C-terminally VSV-G tagged RhsA_ΔNT_ D1324N and RhsI | This study |
| pETDuet-1::PFL_6096_74-CT_D1346N_VSV-G ::PFL_6097 | Co-expression vector for C-terminally VSV-G tagged RhsA_ΔNT_ D1346N and RhsI | This study |
| pETDuet-1::PFL_6096_C538A ::PFL_6097 | Co-expression vector for RhsA_C538A_ and RhsI | This study |
| pETDuet-1::PFL_6096_D322A ::PFL_6097 | Co-expression vector for RhsA_D322A_ and RhsI | This study |
| pETDuet-1::PFL_6096_H530A ::PFL_6097 | Co-expression vector for RhsA_H530A_ and RhsI | This study |
| pETDuet-1::PFL_6096_D318A_D319A_D320A ::PFL_6097 | Co-expression vector for RhsA_D318A, D319A, D320_ and RhsI | This study |
| pETDuet-1::PFL_6096_H304A_P305A ::PFL_6097 | Co-expression vector for RhsA_H304A, P305A_  and RhsI | This study |
| pETDuet-1::PFL_6096_290-CT ::PFL_6097 | Co-expression vector for RhsA_ΔN_ and RhsI | This study |
| pETDuet-1::PFL_6096_1-1323 ::PFL_6097 | Co-expression vector for RhsA_Δtox_ and RhsI | This study |
| pET29b::PFL_6095 | Expression vector for EagR1 | Ahmad et al., eLife, 2020[4] |
| pEXG2::PFL_6096_D1324N | Allelic exchange construct for generating *rhsA*_D1324N | This study |
| pEXG2::PFL_6096_D1346N | Allelic exchange construct for generating *rhsA*_D1346N | This study |
| pSCrhaB2-CV:: PFL_6096_74-CT | Expression vector for RhsA_ΔTMD_ | This study |
| pSCrhaB2-CV:: PFL_6096_74-CT_D1324N | Expression vector for RhsA_ΔTMD_ D1324N | This study |
| pSCrhaB2-CV:: PFL_6096_74-CT_D1346N | Expression vector for RhsA_ΔTMD_ D1346N | This study |
| pPSV39-CV::PFL_6097 | Expression vector for RhsI | Tang et al, JBC, 2018[5] |

**References**

1. Hmelo LR, Borlee BR, Almblad H, Love ME, Randall TE, Tseng BS, et al. Precision-engineering the Pseudomonas aeruginosa genome with two-step allelic exchange. Nat Protoc. 2015;10: 1820–1841. doi:10.1038/nprot.2015.115

2. Cardona ST, Valvano MA. An expression vector containing a rhamnose-inducible promoter provides tightly regulated gene expression in Burkholderia cenocepacia. Plasmid. 2005;54: 219–228. doi:10.1016/j.plasmid.2005.03.004

3. Silverman JM, Agnello DM, Zheng H, Andrews BT, Li M, Catalano CE, et al. Haemolysin Coregulated Protein Is an Exported Receptor and Chaperone of Type VI Secretion Substrates. Mol Cell. 2013;51: 584–593. doi:10.1016/j.molcel.2013.07.025

4. Ahmad S, Tsang KK, Sachar K, Quentin D, Tashin TM, Bullen NP, et al. Structural basis for effector transmembrane domain recognition by type VI secretion system chaperones. Elife. 2020;9: 1–29. doi:10.7554/eLife.62816

5. Tang JY, Bullen NP, Ahmad S, Whitney JC. Diverse NADase effector families mediate interbacterial antagonism via the type VI secretion system. J Biol Chem. 2018;293: 1504–1514. doi:10.1074/jbc.ra117.000178
