# Supplementary material for: Structure of a bacterial Rhs effector exported by the type VI secretion system
Source: PLoS Pathog. 2022 Jan 5;18(1):e1010182. doi: 10.1371/journal.ppat.1010182 (PMC8765631; doi:10.1371/journal.ppat.1010182)
Supplement: S1 Dataset — (ZIP) [file ppat.1010182.s013.zip › S1_file/protein.html]

proteins


Summary

  

# 1. Notes

# 2. Result Statistics

**Figure 1.**
False discovery rate (FDR) curve. X axis is the number of peptide-spectrum matches (PSM) being kept. Y axis is the corresponding FDR.

  


  

**Figure 2.**
PSM score distribution. **(a)**
Distribution of PEAKS peptide score; **(b)**
Scatterplot of PEAKS peptide score versus precursor mass error.

|  |  |  |  |
| --- | --- | --- | --- |
| **(a)**  |  | | **(b)**  |  | |

**Figure 3.**
Distribution of peptide feature detection. **(a)**
Feature m/z distribution; **(b)**
Feature RT distribution

|  |  |  |  |
| --- | --- | --- | --- |
| **(a)**  |  | | **(b)**  |  | |

**Figure 4.**
Distribution of identified peptide features. **(a)**
Feature abundance distribution **(b)**
*De novo*
sequencing validation.

|  |  |  |  |
| --- | --- | --- | --- |
| **(a)**  |  | | **(b)**  |  | |

|  |  |  |  |  |  |  |  |  |  |  |  |  |  |  |  |  |  |  |  |  |  |  |  |  |  |  |  |  |  |  |  |  |  |  |  |  |  |  |  |  |  |  |  |  |  |  |  |  |  |  |  |  |  |  |  |  |  |  |  |  |  |  |  |  |  |  |  |
| --- | --- | --- | --- | --- | --- | --- | --- | --- | --- | --- | --- | --- | --- | --- | --- | --- | --- | --- | --- | --- | --- | --- | --- | --- | --- | --- | --- | --- | --- | --- | --- | --- | --- | --- | --- | --- | --- | --- | --- | --- | --- | --- | --- | --- | --- | --- | --- | --- | --- | --- | --- | --- | --- | --- | --- | --- | --- | --- | --- | --- | --- | --- | --- | --- | --- | --- | --- |
| **Table 1.** Statistics of data.  | # of MS scans | 2472 | | # of MS/MS scans | 7366 | | # of Features | 21984 | | # of Chimera scans | 0 |    **Table 2.** Result filtration parameters.  | Peptide -10lgP | ≥23.3 | | Protein -10lgP | ≥20 | | Proteins unique peptides | ≥0 | | De novo ALC Score | ≥50% |    **Table 3.** Statistics of filtered result.  | Peptide-Spectrum Matches | 1607 | | Peptide sequences | 451 | | Protein groups | 1 | | Proteins | 1 | | Proteins (#Unique Peptides) | 1 (>2); 0 (=2); 0 (=1); | | FDR (Peptide-Spectrum Matches) | 0.1% | | FDR (Peptide Sequences) | 0.2% | | FDR (Protein Group) | 100.0% | | De Novo Only Spectra | 1271 | | # of identified Features | 1225 | | # of identified MS/MS scans | 1607 | | **Table 4.** PTM profile.  | Name | ∆Mass | Position | #PSM | -10lgP | Abundance | AScore || Deamidation | .98 | NQ | 147 | 133.15 | 2.96E8 | 0.00 | | Oxidation | 15.99 | M | 59 | 148.98 | 1.86E8 | 1000.00 | | Carbamidomethyl | 57.02 | C | 52 | 145.90 | 4.41E8 | 1000.00 | |

# 3. Experiment Control

**Figure 5.**
Precursor mass error of peptide-spectrum matches (PSM) in filtered result. **(a)**
Distribution of precursor mass error in ppm; **(b)**
Scatterplot of precursor m/z versus precursor mass error in ppm.

|  |  |  |  |
| --- | --- | --- | --- |
| **(a)**  |  | | **(b)**  |  | |

**Table 5.**
Number of identified peptides in each sample by the number of missed cleavages

|  |  |  |  |  |  |  |  |  |  |  |  |  |  |  |  |  |  |  |
| --- | --- | --- | --- | --- | --- | --- | --- | --- | --- | --- | --- | --- | --- | --- | --- | --- | --- | --- |
| |  |  |  |  |  |  | | --- | --- | --- | --- | --- | --- | | Missed Cleavages | 0 | 1 | 2 | 3 | 4+ | | Tr | 170 | 42 | 2 | 0 | 0 | | AspN | 198 | 30 | 8 | 1 | 0 | |

# 4. Other Information

|  |  |  |  |
| --- | --- | --- | --- |
| **Table 6.** Search parameters.  | Search Engine Name: PEAKS Parent Mass Error Tolerance: 5.0 ppm Fragment Mass Error Tolerance: 0.6 Da Precursor Mass Search Type: monoisotopic Enzyme: Specified by each sample Max Missed Cleavages: 7 Digest Mode: Semispecific Variable Modifications:    Carbamidomethylation: 57.02   Deamidation (NQ): 0.98   Oxidation (M): 15.99 Max Variable PTM Per Peptide: 7 Database: protein1 Taxon: All Contaminant Database: proteases Searched Entry: 11 FDR Estimation: Enabled Merge Options: no merge Precursor Options: corrected Charge Options: no correction Filter Charge: 2 - 8 Process: true Associate chimera: yes | | **Table 7.** Instrument parameters.  | Fractions: 21L153\_Guenther\_MPI\_Tr.raw, 21L153\_Guenther\_MPI\_AspN .raw Ion Source: ESI(nano-spray) Fragmentation Mode: CID, CAD(y and b ions) MS Scan Mode: FT-ICR/Orbitrap MS/MS Scan Mode: Linear Ion Trap | |

  

Protein List

  

|  |
| --- |
| Protein Accession Contains: |
| Protein Description Contains: |
| Peptide Sample Area >= |
| Protein Ptm Contains: |

| Protein Group | Protein ID | Accession | -10lgP | Coverage (%) | Coverage (%) Tr | Coverage (%) AspN | Coverage (%) TrProAla | Coverage (%) AspNProAla | Area Tr | Area AspN | Area TrProAla | Area AspNProAla | #Peptides | #Unique | #Spec Tr | #Spec AspN | #Spec TrProAla | #Spec AspNProAla | PTM | Avg. Mass | Description |
| --- | --- | --- | --- | --- | --- | --- | --- | --- | --- | --- | --- | --- | --- | --- | --- | --- | --- | --- | --- | --- | --- |
| 1 | 2 | protein2|FullLength | 719.22 | 76 | 68 | 69 | 0 | 0 | 7.06E10 | 8.05E10 |  |  | 395 | 395 | 821 | 785 | 0 | 0 | Y | 164912 | user pasted sequence |
| total 1 proteins |
| --- |

  

protein2|FullLength

back to list

  

| Protein Coverage
| Supporting Peptides
|

Protein Coverage:

Supporting Peptides:

| Peptide | Uniq | -10lgP | Mass | Length | ppm | m/z | z | RT | Fraction | Scan | Source File | Area Tr | Area AspN | Area TrProAla | Area AspNProAla | #Feature | #Feature Tr | #Feature AspN | #Feature TrProAla | #Feature AspNProAla | Start | End | PTM |
| --- | --- | --- | --- | --- | --- | --- | --- | --- | --- | --- | --- | --- | --- | --- | --- | --- | --- | --- | --- | --- | --- | --- | --- |
| R.IDM(+15.99)GSIALGAGAFSPGEGLSVR.R | Y | 148.98 | 2120.0571 | 22 | 2.3 | 1061.0383 | 2 | 30.79 | 1 | F1:4197 | 21L153\_Guenther\_MPI\_Tr.raw | 2.34E8 |  |  |  | 6 | 6 | 0 | 0 | 0 | 382 | 403 | Oxidation (M) |
| R.LYYGSGHLHQINLDGQVVSDFQR.D | Y | 145.95 | 2645.2986 | 23 | 1.6 | 1323.6587 | 2 | 27.34 | 1 | F1:3568 | 21L153\_Guenther\_MPI\_Tr.raw | 1.42E9 |  |  |  | 13 | 13 | 0 | 0 | 0 | 984 | 1006 |  |
| R.GNC(+57.02)IAEVDPLGQVTQYR.H | Y | 145.90 | 1918.9207 | 17 | 0.6 | 960.4681 | 2 | 29.25 | 1 | F1:3921 | 21L153\_Guenther\_MPI\_Tr.raw | 5.56E8 |  |  |  | 7 | 7 | 0 | 0 | 0 | 677 | 693 | Carbamidomethylation |
| R.SSNGQLQGYTDPSGYTTLYQYNR.R | Y | 143.41 | 2612.1780 | 23 | 2.1 | 1307.0990 | 2 | 27.30 | 1 | F1:3561 | 21L153\_Guenther\_MPI\_Tr.raw | 4.34E7 |  |  |  | 5 | 5 | 0 | 0 | 0 | 779 | 801 |  |
| R.SSN(+.98)GQLQGYTDPSGYTTLYQYNR.R | Y | 133.15 | 2613.1619 | 23 | 3.9 | 1307.5933 | 2 | 27.40 | 1 | F1:3579 | 21L153\_Guenther\_MPI\_Tr.raw | 4.72E8 |  |  |  | 6 | 6 | 0 | 0 | 0 | 779 | 801 | Deamidation (NQ) |
| R.NENGQLLIESSDGIYR.L | Y | 130.18 | 1806.8748 | 16 | 1.0 | 904.4456 | 2 | 27.21 | 1 | F1:3544 | 21L153\_Guenther\_MPI\_Tr.raw | 5.91E7 |  |  |  | 3 | 3 | 0 | 0 | 0 | 405 | 420 |  |
| K.SEYDSQGNLLGETR.F | Y | 128.20 | 1567.7114 | 14 | 1.2 | 784.8640 | 2 | 20.94 | 1 | F1:2427 | 21L153\_Guenther\_MPI\_Tr.raw | 1.94E9 |  |  |  | 11 | 11 | 0 | 0 | 0 | 1189 | 1202 |  |
| K.ETFAYDAAANLLDGPK.A | Y | 127.29 | 1694.8151 | 16 | 2.1 | 848.4166 | 2 | 31.14 | 1 | F1:4258 | 21L153\_Guenther\_MPI\_Tr.raw | 3.94E8 |  |  |  | 5 | 5 | 0 | 0 | 0 | 1098 | 1113 |  |
| R.HLEYDDSDNLVGLAER.H | Y | 127.12 | 1844.8540 | 16 | 2.2 | 923.4363 | 2 | 26.30 | 1 | F1:3376 | 21L153\_Guenther\_MPI\_Tr.raw | 2.03E9 |  |  |  | 8 | 8 | 0 | 0 | 0 | 1053 | 1068 |  |
| R.LQHSSLLANTLPTAPER.H | Y | 126.38 | 1846.9901 | 17 | 3.2 | 924.5052 | 2 | 24.60 | 1 | F1:3069 | 21L153\_Guenther\_MPI\_Tr.raw | 3.1E9 |  |  |  | 11 | 11 | 0 | 0 | 0 | 1036 | 1052 |  |
| R.HDAHGQVVEIIDAAGR.S | Y | 124.19 | 1686.8438 | 16 | -2.0 | 844.4275 | 2 | 23.50 | 1 | F1:2885 | 21L153\_Guenther\_MPI\_Tr.raw | 4.08E9 |  |  |  | 16 | 16 | 0 | 0 | 0 | 694 | 709 |  |
| R.NEN(+.98)GQLLIESSDGIYR.L | Y | 123.78 | 1807.8588 | 16 | 4.0 | 904.9403 | 2 | 27.69 | 1 | F1:3633 | 21L153\_Guenther\_MPI\_Tr.raw | 2.61E8 |  |  |  | 2 | 2 | 0 | 0 | 0 | 405 | 420 | Deamidation (NQ) |
| R.ISKSEYDSQGNLLGETR.F | Y | 123.24 | 1895.9225 | 17 | 1.9 | 948.9703 | 2 | 19.27 | 1 | F1:2142 | 21L153\_Guenther\_MPI\_Tr.raw | 2E6 |  |  |  | 2 | 2 | 0 | 0 | 0 | 1186 | 1202 |  |
| L.DGPKAGAGLVVHNKLLTYQ.D | Y | 122.86 | 1980.0792 | 19 | 2.5 | 991.0493 | 2 | 23.98 | 2 | F2:2964 | 21L153\_Guenther\_MPI\_AspN.raw |  | 2.92E9 |  |  | 9 | 0 | 9 | 0 | 0 | 1110 | 1128 |  |
| R.YDELGNLTQTQLPDKR.W | Y | 122.66 | 1889.9482 | 16 | 1.1 | 945.9825 | 2 | 24.64 | 1 | F1:3076 | 21L153\_Guenther\_MPI\_Tr.raw | 2.04E9 |  |  |  | 10 | 10 | 0 | 0 | 0 | 964 | 979 |  |
| R.RIDMGSIALGAGAFSPGEGLSVR.R | Y | 122.30 | 2260.1633 | 23 | 2.7 | 1131.0920 | 2 | 32.16 | 1 | F1:4437 | 21L153\_Guenther\_MPI\_Tr.raw | 1.48E8 |  |  |  | 6 | 6 | 0 | 0 | 0 | 381 | 403 |  |
| R.C(+57.02)FYQWTQFADQQWR.V | Y | 122.09 | 1962.8472 | 14 | 3.0 | 982.4338 | 2 | 33.74 | 1 | F1:4708 | 21L153\_Guenther\_MPI\_Tr.raw | 2.11E6 |  |  |  | 2 | 2 | 0 | 0 | 0 | 538 | 551 | Carbamidomethylation |
| K.VLGADDDLDFTLPGLMPIEWQR.F | Y | 121.61 | 2500.2307 | 22 | 3.5 | 1251.1270 | 2 | 34.64 | 1 | F1:4857 | 21L153\_Guenther\_MPI\_Tr.raw | 8.1E6 |  |  |  | 6 | 6 | 0 | 0 | 0 | 314 | 335 |  |
| R.YDELGNLTQTQLPDK.R | Y | 120.71 | 1733.8472 | 15 | 2.3 | 867.9329 | 2 | 26.42 | 1 | F1:3399 | 21L153\_Guenther\_MPI\_Tr.raw | 2.68E8 |  |  |  | 6 | 6 | 0 | 0 | 0 | 964 | 978 |  |
| R.IDMGSIALGAGAFSPGEGLSVR.R | Y | 118.69 | 2104.0623 | 22 | 2.3 | 1053.0408 | 2 | 33.36 | 1 | F1:4647 | 21L153\_Guenther\_MPI\_Tr.raw | 6.07E8 |  |  |  | 4 | 4 | 0 | 0 | 0 | 382 | 403 |  |
| T.DNLGNQQALSFSY.D | Y | 118.24 | 1455.6630 | 13 | 2.2 | 728.8403 | 2 | 28.91 | 2 | F2:3881 | 21L153\_Guenther\_MPI\_AspN.raw |  | 4.8E8 |  |  | 3 | 0 | 3 | 0 | 0 | 932 | 944 |  |
| R.LLALTNENGESYR.F | Y | 116.46 | 1478.7365 | 13 | -2.1 | 740.3740 | 2 | 23.52 | 1 | F1:2889 | 21L153\_Guenther\_MPI\_Tr.raw | 5.28E8 |  |  |  | 3 | 3 | 0 | 0 | 0 | 826 | 838 |  |
| R.YAYSYDETGNLIAETDPLGH.S | Y | 116.25 | 2227.9910 | 20 | 2.9 | 1115.0060 | 2 | 31.96 | 1 | F1:4402 | 21L153\_Guenther\_MPI\_Tr.raw | 4.98E6 |  |  |  | 2 | 2 | 0 | 0 | 0 | 626 | 645 |  |
| R.RNENGQLLIESSDGIYR.L | Y | 111.87 | 1962.9758 | 17 | 1.4 | 982.4965 | 2 | 25.20 | 1 | F1:3172 | 21L153\_Guenther\_MPI\_Tr.raw | 2.32E8 |  |  |  | 5 | 5 | 0 | 0 | 0 | 404 | 420 |  |
| H.PVHAATGAKVLGADD.D | Y | 109.62 | 1420.7310 | 15 | -0.4 | 711.3725 | 2 | 14.26 | 2 | F2:1202 | 21L153\_Guenther\_MPI\_AspN.raw |  | 1.35E8 |  |  | 6 | 0 | 6 | 0 | 0 | 305 | 319 |  |
| R.EVLVSYDYDAAGDLAQVR.D | Y | 109.36 | 1982.9585 | 18 | 2.1 | 992.4886 | 2 | 28.67 | 1 | F1:3814 | 21L153\_Guenther\_MPI\_Tr.raw | 9.44E7 |  |  |  | 6 | 6 | 0 | 0 | 0 | 492 | 509 |  |
| R.LLALTNEN(+.98)GESYR.F | Y | 109.34 | 1479.7205 | 13 | -0.8 | 740.8669 | 2 | 23.86 | 1 | F1:2943 | 21L153\_Guenther\_MPI\_Tr.raw | 1.49E9 |  |  |  | 6 | 6 | 0 | 0 | 0 | 826 | 838 | Deamidation (NQ) |
| K.VLGADDDLDFTLPGLM(+15.99)PIEWQR.F | Y | 108.54 | 2516.2256 | 22 | 3.3 | 1259.1243 | 2 | 34.20 | 1 | F1:4792 | 21L153\_Guenther\_MPI\_Tr.raw | 1.82E7 |  |  |  | 3 | 3 | 0 | 0 | 0 | 314 | 335 | Oxidation (M) |
| I.DMGSIALGAGAFSPGEGLSVR.R | Y | 108.39 | 1990.9781 | 21 | 2.6 | 996.4989 | 2 | 32.42 | 2 | F2:4495 | 21L153\_Guenther\_MPI\_AspN.raw |  | 1.53E6 |  |  | 1 | 0 | 1 | 0 | 0 | 383 | 403 |  |
| T.DPLGGRYAYSY.D | Y | 108.36 | 1260.5774 | 11 | 2.0 | 631.2972 | 2 | 24.22 | 2 | F2:3011 | 21L153\_Guenther\_MPI\_AspN.raw |  | 1.3E9 |  |  | 4 | 0 | 4 | 0 | 0 | 620 | 630 |  |
| L.DC(+57.02)SGYPTRFEY.D | Y | 107.69 | 1393.5608 | 11 | 1.8 | 697.7889 | 2 | 24.82 | 2 | F2:3125 | 21L153\_Guenther\_MPI\_AspN.raw |  | 3.13E8 |  |  | 3 | 0 | 3 | 0 | 0 | 726 | 736 | Carbamidomethylation |
| H.PVHAATGAKVLGADDDL.D | Y | 105.99 | 1648.8420 | 17 | 1.4 | 825.4294 | 2 | 20.79 | 2 | F2:2382 | 21L153\_Guenther\_MPI\_AspN.raw | 6.78E6 | 3.88E9 |  |  | 11 | 5 | 6 | 0 | 0 | 305 | 321 |  |
| R.EPYYIEEQNLRFQGQYL.D | Y | 105.84 | 2189.0430 | 17 | 1.3 | 1095.5302 | 2 | 31.67 | 2 | F2:4367 | 21L153\_Guenther\_MPI\_AspN.raw |  | 7.95E7 |  |  | 4 | 0 | 4 | 0 | 0 | 1283 | 1299 |  |
| Y.DALGQLLEEHTVAGSLIHRY.D | Y | 105.15 | 2221.1492 | 20 | 3.8 | 741.3931 | 3 | 32.80 | 2 | F2:4562 | 21L153\_Guenther\_MPI\_AspN.raw |  | 4.5E8 |  |  | 4 | 0 | 4 | 0 | 0 | 945 | 964 |  |
| Y.DATGRIIASQDGLQGQKETFAY.D | Y | 104.83 | 2368.1658 | 22 | 4.4 | 1185.0953 | 2 | 25.62 | 2 | F2:3272 | 21L153\_Guenther\_MPI\_AspN.raw |  | 4.89E7 |  |  | 6 | 0 | 6 | 0 | 0 | 1081 | 1102 |  |
| R.SSNGQ(+.98)LQGYTDPSGYTTLYQY.N | Y | 103.79 | 2343.0178 | 21 | 3.7 | 1172.5205 | 2 | 30.23 | 1 | F1:4096 | 21L153\_Guenther\_MPI\_Tr.raw | 9.64E6 |  |  |  | 2 | 2 | 0 | 0 | 0 | 779 | 799 | Deamidation (NQ) |
| V.DPLGQVTQYRH.D | Y | 103.70 | 1312.6523 | 11 | -0.4 | 657.3332 | 2 | 18.03 | 2 | F2:1884 | 21L153\_Guenther\_MPI\_AspN.raw |  | 2.8E9 |  |  | 5 | 0 | 5 | 0 | 0 | 684 | 694 |  |
| R.LSEQQDLDGSAK.R | Y | 103.44 | 1289.6099 | 12 | -0.3 | 645.8120 | 2 | 17.30 | 1 | F1:1805 | 21L153\_Guenther\_MPI\_Tr.raw | 1.62E9 |  |  |  | 8 | 8 | 0 | 0 | 0 | 847 | 858 |  |
| R.NQQ(+.98)TSLYVYEDLGHAPLAR.V | Y | 102.86 | 2175.0596 | 19 | 2.4 | 1088.5397 | 2 | 29.77 | 1 | F1:4017 | 21L153\_Guenther\_MPI\_Tr.raw | 3.62E6 |  |  |  | 1 | 1 | 0 | 0 | 0 | 1216 | 1234 | Deamidation (NQ) |
| R.YQVWGNTVEEIREPY.Y | Y | 102.12 | 1881.8896 | 15 | 2.6 | 941.9545 | 2 | 30.26 | 1 | F1:4103 | 21L153\_Guenther\_MPI\_Tr.raw | 5.65E6 |  |  |  | 3 | 3 | 0 | 0 | 0 | 1271 | 1285 |  |
| K.ETFAYDAAAN(+.98)LLDGPK.A | Y | 101.18 | 1695.7991 | 16 | 2.2 | 848.9087 | 2 | 29.35 | 1 | F1:3940 | 21L153\_Guenther\_MPI\_Tr.raw | 9.65E6 |  |  |  | 4 | 4 | 0 | 0 | 0 | 1098 | 1113 | Deamidation (NQ) |
| R.WNSSGQLLEHLDC(+57.02)SGYPTR.F | Y | 100.99 | 2219.0066 | 19 | 4.0 | 1110.5150 | 2 | 27.73 | 1 | F1:3640 | 21L153\_Guenther\_MPI\_Tr.raw | 1.73E8 |  |  |  | 4 | 4 | 0 | 0 | 0 | 715 | 733 | Carbamidomethylation |
| R.N(+.98)QQTSLYVYEDLGHAPLAR.V | Y | 100.25 | 2175.0596 | 19 | 2.4 | 1088.5397 | 2 | 29.74 | 1 | F1:4012 | 21L153\_Guenther\_MPI\_Tr.raw | 3.62E6 |  |  |  | 1 | 1 | 0 | 0 | 0 | 1216 | 1234 | Deamidation (NQ) |
| H.PVHAATGAKVLGAD.D | Y | 99.63 | 1305.7041 | 14 | -0.2 | 653.8592 | 2 | 14.19 | 2 | F2:1188 | 21L153\_Guenther\_MPI\_AspN.raw | 1.83E5 | 9.67E8 |  |  | 7 | 1 | 6 | 0 | 0 | 305 | 318 |  |
| T.DPAGESWHLRY.D | Y | 99.00 | 1329.6101 | 11 | 2.1 | 665.8137 | 2 | 24.77 | 2 | F2:3114 | 21L153\_Guenther\_MPI\_AspN.raw |  | 1.74E9 |  |  | 6 | 0 | 6 | 0 | 0 | 663 | 673 |  |
| Y.DYDAAGDLAQVR.D | Y | 98.85 | 1292.5996 | 12 | 0.5 | 647.3074 | 2 | 22.39 | 1 | F1:2679 | 21L153\_Guenther\_MPI\_Tr.raw | 1.56E7 |  |  |  | 3 | 3 | 0 | 0 | 0 | 498 | 509 |  |
| R.RNEN(+.98)GQLLIESSDGIYR.L | Y | 98.27 | 1963.9598 | 17 | 2.0 | 982.9891 | 2 | 26.02 | 1 | F1:3322 | 21L153\_Guenther\_MPI\_Tr.raw | 9.45E8 |  |  |  | 4 | 4 | 0 | 0 | 0 | 404 | 420 | Deamidation (NQ) |
| R.RN(+.98)ENGQLLIESSDGIYR.L | Y | 96.91 | 1963.9598 | 17 | 3.0 | 655.6625 | 3 | 26.14 | 1 | F1:3345 | 21L153\_Guenther\_MPI\_Tr.raw | 6.46E8 |  |  |  | 1 | 1 | 0 | 0 | 0 | 404 | 420 | Deamidation (NQ) |
| R.SSNGQLQ(+.98)GYTDPSGYTTLYQY.N | Y | 96.28 | 2343.0178 | 21 | 3.6 | 1172.5204 | 2 | 30.17 | 1 | F1:4086 | 21L153\_Guenther\_MPI\_Tr.raw | 9.64E6 |  |  |  | 1 | 1 | 0 | 0 | 0 | 779 | 799 | Deamidation (NQ) |
| Y.DPLNQLTAASFT.D | Y | 96.17 | 1276.6299 | 12 | 1.8 | 639.3234 | 2 | 31.37 | 2 | F2:4313 | 21L153\_Guenther\_MPI\_AspN.raw |  | 6.3E8 |  |  | 1 | 0 | 1 | 0 | 0 | 920 | 931 |  |
| R.RIDM(+15.99)GSIALGAGAFSPGEGLSVR.R | Y | 96.00 | 2276.1582 | 23 | 0.7 | 759.7272 | 3 | 29.06 | 1 | F1:3886 | 21L153\_Guenther\_MPI\_Tr.raw | 7.2E7 |  |  |  | 3 | 3 | 0 | 0 | 0 | 381 | 403 | Oxidation (M) |
| Y.TDPSGYTTLYQYNR.R | Y | 95.71 | 1677.7634 | 14 | 3.0 | 839.8915 | 2 | 24.11 | 1 | F1:2986 | 21L153\_Guenther\_MPI\_Tr.raw | 6.32E6 |  |  |  | 3 | 3 | 0 | 0 | 0 | 788 | 801 |  |
| W.DAGNRLSEQQDLDGSAKRYGY.D | Y | 95.58 | 2342.0886 | 21 | 0.9 | 781.7042 | 3 | 19.79 | 2 | F2:2202 | 21L153\_Guenther\_MPI\_AspN.raw |  | 2.53E7 |  |  | 6 | 0 | 6 | 0 | 0 | 842 | 862 |  |
| R.GNCIAEVDPLGQVTQYR.H | Y | 95.44 | 1861.8992 | 17 | 2.0 | 931.9587 | 2 | 30.45 | 1 | F1:4134 | 21L153\_Guenther\_MPI\_Tr.raw | 3.1E6 |  |  |  | 2 | 2 | 0 | 0 | 0 | 677 | 693 |  |
| I.DALGEHTRFHY.D | Y | 95.29 | 1344.6211 | 11 | 0.5 | 673.3182 | 2 | 17.79 | 2 | F2:1842 | 21L153\_Guenther\_MPI\_AspN.raw |  | 1.76E9 |  |  | 6 | 0 | 6 | 0 | 0 | 747 | 757 |  |
| R.YDELGN(+.98)LTQTQLPDKR.W | Y | 95.24 | 1890.9323 | 16 | 2.2 | 946.4755 | 2 | 24.53 | 1 | F1:3058 | 21L153\_Guenther\_MPI\_Tr.raw | 7.25E8 |  |  |  | 2 | 2 | 0 | 0 | 0 | 964 | 979 | Deamidation (NQ) |
| T.DPLGHSHSTLWLEHWSLPQTLT.D | Y | 94.90 | 2554.2605 | 22 | 4.6 | 1278.1434 | 2 | 33.70 | 2 | F2:4719 | 21L153\_Guenther\_MPI\_AspN.raw |  | 2.26E8 |  |  | 4 | 0 | 4 | 0 | 0 | 641 | 662 |  |
| T.DNLNQTWAFEWN.D | Y | 93.57 | 1536.6633 | 12 | -3.4 | 769.3364 | 2 | 33.98 | 2 | F2:4769 | 21L153\_Guenther\_MPI\_AspN.raw |  | 1.41E7 |  |  | 5 | 0 | 5 | 0 | 0 | 599 | 610 |  |
| R.DTFEQAQLELSYSTLWPGR.I | Y | 93.25 | 2240.0750 | 19 | 1.0 | 747.6997 | 3 | 33.92 | 1 | F1:4743 | 21L153\_Guenther\_MPI\_Tr.raw | 9.91E6 |  |  |  | 2 | 2 | 0 | 0 | 0 | 460 | 478 |  |
| Q.DGLQGQKETFAY.D | Y | 92.88 | 1355.6357 | 12 | 2.8 | 678.8270 | 2 | 23.11 | 2 | F2:2807 | 21L153\_Guenther\_MPI\_AspN.raw |  | 1.51E9 |  |  | 6 | 0 | 6 | 0 | 0 | 1091 | 1102 |  |
| W.SLPYEISVQIEPHPEGGER.L | Y | 92.22 | 2136.0486 | 19 | 3.5 | 1069.0353 | 2 | 27.97 | 1 | F1:3685 | 21L153\_Guenther\_MPI\_Tr.raw | 3.91E7 |  |  |  | 4 | 4 | 0 | 0 | 0 | 353 | 371 |  |
| R.M(+15.99)IEHQLATGLR.C | Y | 91.83 | 1283.6656 | 11 | -0.3 | 642.8399 | 2 | 15.82 | 1 | F1:1538 | 21L153\_Guenther\_MPI\_Tr.raw | 1.3E9 |  |  |  | 9 | 9 | 0 | 0 | 0 | 527 | 537 | Oxidation (M) |
| Y.DSQGNLLGETRFVW.D | Y | 90.70 | 1620.7896 | 14 | -2.4 | 811.4001 | 2 | 33.88 | 2 | F2:4752 | 21L153\_Guenther\_MPI\_AspN.raw |  | 2.92E8 |  |  | 3 | 0 | 3 | 0 | 0 | 1192 | 1205 |  |
| R.IIASQDGLQGQK.E | Y | 90.66 | 1256.6725 | 12 | 1.1 | 629.3442 | 2 | 17.93 | 1 | F1:1919 | 21L153\_Guenther\_MPI\_Tr.raw | 2.35E9 |  |  |  | 7 | 7 | 0 | 0 | 0 | 1086 | 1097 |  |
| W.DAGNRLSEQQ.D | Y | 90.56 | 1116.5160 | 10 | 0.6 | 559.2656 | 2 | 12.49 | 2 | F2:852 | 21L153\_Guenther\_MPI\_AspN.raw |  | 1.64E7 |  |  | 3 | 0 | 3 | 0 | 0 | 842 | 851 |  |
| V.DGLGAQQKIRYYHN.D | Y | 89.80 | 1661.8274 | 14 | 2.3 | 831.9229 | 2 | 14.65 | 2 | F2:1278 | 21L153\_Guenther\_MPI\_AspN.raw |  | 3.31E9 |  |  | 9 | 0 | 9 | 0 | 0 | 1236 | 1249 |  |
| L.EEHTVAGSLIHRY.D | Y | 89.12 | 1510.7528 | 13 | 0.8 | 756.3843 | 2 | 18.27 | 2 | F2:1927 | 21L153\_Guenther\_MPI\_AspN.raw |  | 3.09E9 |  |  | 8 | 0 | 8 | 0 | 0 | 952 | 964 |  |
| A.EHFQRSSN(+.98)GQLQGYT.D | Y | 88.75 | 1751.7863 | 15 | 1.2 | 876.9015 | 2 | 15.01 | 2 | F2:1345 | 21L153\_Guenther\_MPI\_AspN.raw |  | 3.38E8 |  |  | 5 | 0 | 5 | 0 | 0 | 774 | 788 | Deamidation (NQ) |
| R.LHLEYDSSGR.L | Y | 88.70 | 1175.5571 | 10 | 0.1 | 588.7859 | 2 | 15.82 | 1 | F1:1539 | 21L153\_Guenther\_MPI\_Tr.raw | 1.99E9 |  |  |  | 10 | 10 | 0 | 0 | 0 | 445 | 454 |  |
| Y.DLEAGITQVT.D | Y | 88.57 | 1045.5292 | 10 | 0.8 | 523.7723 | 2 | 25.11 | 2 | F2:3179 | 21L153\_Guenther\_MPI\_AspN.raw |  | 2.85E8 |  |  | 3 | 0 | 3 | 0 | 0 | 568 | 577 |  |
| F.AYDAAANLLDGPK.A | Y | 88.37 | 1317.6564 | 13 | -0.1 | 659.8354 | 2 | 26.33 | 1 | F1:3382 | 21L153\_Guenther\_MPI\_Tr.raw | 1.56E7 |  |  |  | 3 | 3 | 0 | 0 | 0 | 1101 | 1113 |  |
| Q.EQRNQQTSLYVYE.D | Y | 88.12 | 1656.7743 | 13 | 2.0 | 829.3961 | 2 | 21.99 | 2 | F2:2604 | 21L153\_Guenther\_MPI\_AspN.raw |  | 7.82E8 |  |  | 3 | 0 | 3 | 0 | 0 | 1213 | 1225 |  |
| R.MIEHQLATGLR.C | Y | 87.00 | 1267.6707 | 11 | 0.4 | 634.8428 | 2 | 17.84 | 1 | F1:1902 | 21L153\_Guenther\_MPI\_Tr.raw | 1.51E9 |  |  |  | 8 | 8 | 0 | 0 | 0 | 527 | 537 |  |
| R.YQVWGNTVEEIR.E | Y | 86.26 | 1492.7310 | 12 | 3.3 | 747.3752 | 2 | 27.66 | 1 | F1:3628 | 21L153\_Guenther\_MPI\_Tr.raw | 8.04E7 |  |  |  | 3 | 3 | 0 | 0 | 0 | 1271 | 1282 |  |
| R.NQTSSGETLVR.M | Y | 86.03 | 1190.5891 | 11 | -0.3 | 596.3016 | 2 | 18.06 | 1 | F1:1943 | 21L153\_Guenther\_MPI\_Tr.raw | 1.65E9 |  |  |  | 10 | 10 | 0 | 0 | 0 | 1166 | 1176 |  |
| R.GNVKAIIDALGEHTR.F | Y | 85.70 | 1592.8634 | 15 | 1.5 | 531.9625 | 3 | 27.74 | 1 | F1:3642 | 21L153\_Guenther\_MPI\_Tr.raw | 6.14E6 |  |  |  | 3 | 3 | 0 | 0 | 0 | 740 | 754 |  |
| R.DGLLGAGWSLPY.E | Y | 85.56 | 1247.6187 | 12 | 0.9 | 624.8171 | 2 | 34.57 | 2 | F2:4865 | 21L153\_Guenther\_MPI\_AspN.raw | 3.09E5 | 1.41E7 |  |  | 4 | 1 | 3 | 0 | 0 | 345 | 356 |  |
| L.DFTLPGLMPIEWQR.F | Y | 85.02 | 1701.8549 | 14 | 2.1 | 851.9365 | 2 | 34.36 | 2 | F2:4835 | 21L153\_Guenther\_MPI\_AspN.raw |  | 1.07E7 |  |  | 3 | 0 | 3 | 0 | 0 | 322 | 335 |  |
| R.LFEPLPGDSSR.L | Y | 84.66 | 1216.6088 | 11 | 0.1 | 609.3117 | 2 | 24.22 | 1 | F1:3003 | 21L153\_Guenther\_MPI\_Tr.raw | 2.25E9 |  |  |  | 9 | 9 | 0 | 0 | 0 | 421 | 431 |  |
| D.PSGYTTLYQYNR.R | Y | 84.42 | 1461.6888 | 12 | -0.9 | 731.8510 | 2 | 23.00 | 1 | F1:2787 | 21L153\_Guenther\_MPI\_Tr.raw | 6.02E7 |  |  |  | 3 | 3 | 0 | 0 | 0 | 790 | 801 |  |
| R.YQVWGNTVEEIREPYYIEEQNLR.F | Y | 84.34 | 2927.4089 | 23 | 3.2 | 976.8134 | 3 | 31.65 | 1 | F1:4347 | 21L153\_Guenther\_MPI\_Tr.raw | 8.91E7 |  |  |  | 5 | 5 | 0 | 0 | 0 | 1271 | 1293 |  |
| H.PVHAATGAKVLGA.D | Y | 84.30 | 1190.6771 | 13 | 0.1 | 596.3459 | 2 | 14.38 | 2 | F2:1225 | 21L153\_Guenther\_MPI\_AspN.raw |  | 3.11E8 |  |  | 6 | 0 | 6 | 0 | 0 | 305 | 317 |  |
| C.IAEVDPLGQVTQYR.H | Y | 83.87 | 1587.8257 | 14 | 2.2 | 794.9219 | 2 | 28.07 | 1 | F1:3703 | 21L153\_Guenther\_MPI\_Tr.raw | 5.88E6 |  |  |  | 2 | 2 | 0 | 0 | 0 | 680 | 693 |  |
| R.EPYYIEEQNLR.F | Y | 83.79 | 1452.6885 | 11 | 0.4 | 727.3518 | 2 | 23.56 | 1 | F1:2895 | 21L153\_Guenther\_MPI\_Tr.raw | 2.97E8 |  |  |  | 3 | 3 | 0 | 0 | 0 | 1283 | 1293 |  |
| L.DRETGLHFNTFRFY.D | Y | 83.09 | 1801.8535 | 14 | 4.0 | 901.9376 | 2 | 29.91 | 2 | F2:4053 | 21L153\_Guenther\_MPI\_AspN.raw |  | 7.6E8 |  |  | 7 | 0 | 7 | 0 | 0 | 1300 | 1313 |  |
| Y.DAYGRLLALTNENG.E | Y | 82.75 | 1505.7474 | 14 | 0.6 | 753.8815 | 2 | 29.24 | 2 | F2:3937 | 21L153\_Guenther\_MPI\_AspN.raw |  | 1.44E8 |  |  | 3 | 0 | 3 | 0 | 0 | 821 | 834 |  |
| R.HWTDEGDSYQYDYDLEAGITQVTDSLQR.L | Y | 82.66 | 3304.4431 | 28 | 3.7 | 1102.4924 | 3 | 33.86 | 1 | F1:4731 | 21L153\_Guenther\_MPI\_Tr.raw | 3.78E7 |  |  |  | 3 | 3 | 0 | 0 | 0 | 555 | 582 |  |
| N.LDGQVVSDFQR.D | Y | 82.11 | 1262.6255 | 11 | 0.2 | 632.3201 | 2 | 22.49 | 1 | F1:2697 | 21L153\_Guenther\_MPI\_Tr.raw | 8.22E6 |  |  |  | 3 | 3 | 0 | 0 | 0 | 996 | 1006 |  |
| K.VVSAGPISPNVTIGGGSVVVR.E | Y | 81.74 | 1964.1055 | 21 | 3.7 | 983.0636 | 2 | 27.55 | 1 | F1:3607 | 21L153\_Guenther\_MPI\_Tr.raw | 5.61E6 |  |  |  | 1 | 1 | 0 | 0 | 0 | 224 | 244 |  |
| R.FDYDAYGR.L | Y | 81.71 | 1005.4192 | 8 | -1.5 | 503.7161 | 2 | 21.21 | 1 | F1:2475 | 21L153\_Guenther\_MPI\_Tr.raw | 1E9 |  |  |  | 12 | 12 | 0 | 0 | 0 | 818 | 825 |  |
| S.DGIYRLFEPLPG.D | Y | 81.34 | 1375.7135 | 12 | -1.4 | 688.8630 | 2 | 33.88 | 2 | F2:4751 | 21L153\_Guenther\_MPI\_AspN.raw |  | 8.57E8 |  |  | 3 | 0 | 3 | 0 | 0 | 416 | 427 |  |
| R.YYHNDLN(+.98)GLPQQLC(+57.02)EPDGHSVWQAR.Y | Y | 81.28 | 2997.3464 | 25 | 2.7 | 1000.1254 | 3 | 27.19 | 1 | F1:3540 | 21L153\_Guenther\_MPI\_Tr.raw | 1.63E8 |  |  |  | 5 | 5 | 0 | 0 | 0 | 1246 | 1270 | Deamidation (NQ); Carbamidomethylation |
| K.TPGVGLAVTVLM(+15.99)MLK.G | Y | 81.26 | 1544.8669 | 15 | 1.2 | 773.4417 | 2 | 34.85 | 1 | F1:4887 | 21L153\_Guenther\_MPI\_Tr.raw | 1.2E5 |  |  |  | 1 | 1 | 0 | 0 | 0 | 251 | 265 | Oxidation (M) |
| K.AIIDALGEHTR.F | Y | 81.24 | 1194.6356 | 11 | -1.1 | 598.3244 | 2 | 21.89 | 1 | F1:2590 | 21L153\_Guenther\_MPI\_Tr.raw | 2.75E9 |  |  |  | 8 | 8 | 0 | 0 | 0 | 744 | 754 |  |
| R.LLYIDEQGR.R | Y | 81.20 | 1105.5768 | 9 | 0.2 | 553.7958 | 2 | 23.21 | 1 | F1:2829 | 21L153\_Guenther\_MPI\_Tr.raw | 1.33E9 |  |  |  | 9 | 9 | 0 | 0 | 0 | 372 | 380 |  |
| R.FAYDAEHR.L | Y | 80.78 | 1007.4460 | 8 | -0.6 | 504.7300 | 2 | 13.56 | 1 | F1:1129 | 21L153\_Guenther\_MPI\_Tr.raw | 2.23E9 |  |  |  | 13 | 13 | 0 | 0 | 0 | 1153 | 1160 |  |
| N.TVEEIREPYYIEEQNLR.F | Y | 80.76 | 2180.0750 | 17 | 2.3 | 1091.0472 | 2 | 26.18 | 1 | F1:3353 | 21L153\_Guenther\_MPI\_Tr.raw | 1.78E8 |  |  |  | 5 | 5 | 0 | 0 | 0 | 1277 | 1293 |  |
| R.ARLQHSSLLANTLPTAPERHLEY.D | Y | 80.07 | 2616.3772 | 23 | 0.8 | 873.1337 | 3 | 25.05 | 2 | F2:3169 | 21L153\_Guenther\_MPI\_AspN.raw |  | 8.62E7 |  |  | 6 | 0 | 6 | 0 | 0 | 1034 | 1056 |  |
| R.FYDPDIGR.F | Y | 79.39 | 981.4556 | 8 | -1.7 | 491.7342 | 2 | 20.43 | 1 | F1:2344 | 21L153\_Guenther\_MPI\_Tr.raw | 1.95E9 |  |  |  | 8 | 8 | 0 | 0 | 0 | 1312 | 1319 |  |
| Y.VYEDLGHAPLAR.V | Y | 79.21 | 1339.6884 | 12 | -0.6 | 670.8511 | 2 | 18.93 | 1 | F1:2081 | 21L153\_Guenther\_MPI\_Tr.raw | 1.6E7 |  |  |  | 5 | 5 | 0 | 0 | 0 | 1223 | 1234 |  |
| P.DIGRFTTPDPIGLAGGLNLY.Q | Y | 78.97 | 2089.0842 | 20 | 4.0 | 1045.5536 | 2 | 34.05 | 2 | F2:4783 | 21L153\_Guenther\_MPI\_AspN.raw |  | 2.15E6 |  |  | 1 | 0 | 1 | 0 | 0 | 1316 | 1335 |  |
| Y.EISVQIEPHPEGGER.L | Y | 78.88 | 1675.8165 | 15 | 0.9 | 838.9163 | 2 | 19.27 | 1 | F1:2143 | 21L153\_Guenther\_MPI\_Tr.raw | 1.4E7 |  |  |  | 5 | 5 | 0 | 0 | 0 | 357 | 371 |  |
| K.HPPM(+15.99)PVQYLAEGSEK.V | Y | 78.43 | 1697.8082 | 15 | -1.4 | 566.9426 | 3 | 20.93 | 1 | F1:2425 | 21L153\_Guenther\_MPI\_Tr.raw | 2.77E6 |  |  |  | 4 | 4 | 0 | 0 | 0 | 189 | 203 | Oxidation (M) |
| D.PLNQLTAASFT.D | Y | 77.83 | 1161.6030 | 11 | 0.4 | 581.8090 | 2 | 28.75 | 2 | F2:3851 | 21L153\_Guenther\_MPI\_AspN.raw |  | 2.91E7 |  |  | 2 | 0 | 2 | 0 | 0 | 921 | 931 |  |
| F.SYDALGQLLEEHTVAGSLIHR.Y | Y | 77.75 | 2308.1812 | 21 | 1.4 | 770.4021 | 3 | 32.99 | 1 | F1:4582 | 21L153\_Guenther\_MPI\_Tr.raw | 9.84E7 |  |  |  | 3 | 3 | 0 | 0 | 0 | 943 | 963 |  |
| N.DNRLHLEY.D | Y | 77.24 | 1058.5145 | 8 | 0.8 | 530.2650 | 2 | 22.13 | 2 | F2:2633 | 21L153\_Guenther\_MPI\_AspN.raw |  | 2.54E9 |  |  | 9 | 0 | 9 | 0 | 0 | 442 | 449 |  |
| R.EVLRTQGQISTRSEY.D | Y | 76.71 | 1765.8959 | 15 | 0.4 | 883.9556 | 2 | 15.75 | 2 | F2:1477 | 21L153\_Guenther\_MPI\_AspN.raw |  | 3.85E8 |  |  | 6 | 0 | 6 | 0 | 0 | 1012 | 1026 |  |
| R.FTTPDPIGLAGGLNLY.Q | Y | 76.60 | 1647.8508 | 16 | 1.9 | 824.9343 | 2 | 34.27 | 1 | F1:4803 | 21L153\_Guenther\_MPI\_Tr.raw | 2.44E6 |  |  |  | 3 | 3 | 0 | 0 | 0 | 1320 | 1335 |  |
| Y.GTGLSAVPAQPIAPIIHR.F | Y | 76.60 | 1797.0260 | 18 | 2.3 | 899.5223 | 2 | 26.25 | 1 | F1:3366 | 21L153\_Guenther\_MPI\_Tr.raw | 8.71E7 |  |  |  | 6 | 6 | 0 | 0 | 0 | 879 | 896 |  |
| R.RFAYDAGHR.M | Y | 76.03 | 1091.5260 | 9 | 2.3 | 364.8501 | 3 | 13.02 | 1 | F1:1019 | 21L153\_Guenther\_MPI\_Tr.raw | 3.09E8 |  |  |  | 6 | 6 | 0 | 0 | 0 | 518 | 526 |  |
| R.DAVGRLLAKITDDGRSQYTY.D | Y | 75.72 | 2241.1389 | 20 | 0.4 | 748.0539 | 3 | 31.01 | 2 | F2:4250 | 21L153\_Guenther\_MPI\_AspN.raw |  | 9.92E6 |  |  | 3 | 0 | 3 | 0 | 0 | 900 | 919 |  |
| L.SAVPAQPIAPIIHR.F | Y | 75.48 | 1468.8513 | 14 | -0.4 | 735.4326 | 2 | 22.77 | 1 | F1:2745 | 21L153\_Guenther\_MPI\_Tr.raw | 5.93E7 |  |  |  | 6 | 6 | 0 | 0 | 0 | 883 | 896 |  |
| R.RNENGQ(+.98)LLIESSDGIYR.L | Y | 75.47 | 1963.9598 | 17 | 1.6 | 655.6616 | 3 | 26.05 | 1 | F1:3327 | 21L153\_Guenther\_MPI\_Tr.raw | 6.46E8 |  |  |  | 1 | 1 | 0 | 0 | 0 | 404 | 420 | Deamidation (NQ) |
| R.LLSSQLADGR.A | Y | 75.08 | 1058.5720 | 10 | -1.5 | 530.2925 | 2 | 16.39 | 1 | F1:1643 | 21L153\_Guenther\_MPI\_Tr.raw | 2.52E9 |  |  |  | 10 | 10 | 0 | 0 | 0 | 763 | 772 |  |
| R.ETGLHFNTFR.F | Y | 74.93 | 1220.5938 | 10 | -2.0 | 611.3029 | 2 | 23.67 | 1 | F1:2914 | 21L153\_Guenther\_MPI\_Tr.raw | 2.73E9 |  |  |  | 14 | 14 | 0 | 0 | 0 | 1302 | 1311 |  |
| Y.RHDAHGQVVEIIDAAGR.S | Y | 74.78 | 1842.9448 | 17 | -1.2 | 615.3215 | 3 | 22.14 | 1 | F1:2632 | 21L153\_Guenther\_MPI\_Tr.raw | 6.28E6 |  |  |  | 3 | 3 | 0 | 0 | 0 | 693 | 709 |  |
| R.SQYTYDPLNQLTAA.S | Y | 74.57 | 1583.7467 | 14 | 1.0 | 792.8814 | 2 | 32.03 | 1 | F1:4414 | 21L153\_Guenther\_MPI\_Tr.raw | 4.7E6 |  |  |  | 3 | 3 | 0 | 0 | 0 | 915 | 928 |  |
| R.LQHSSLLAN(+.98)TLPTAPER.H | Y | 74.30 | 1847.9741 | 17 | 2.2 | 617.0000 | 3 | 24.50 | 1 | F1:3051 | 21L153\_Guenther\_MPI\_Tr.raw | 1.07E8 |  |  |  | 1 | 1 | 0 | 0 | 0 | 1036 | 1052 | Deamidation (NQ) |
| R.QLFHYDATGR.I | Y | 74.16 | 1206.5781 | 10 | -0.6 | 604.2960 | 2 | 19.33 | 1 | F1:2153 | 21L153\_Guenther\_MPI\_Tr.raw | 2.03E9 |  |  |  | 9 | 9 | 0 | 0 | 0 | 1076 | 1085 |  |
| P.DQQREVLVSY.D | Y | 74.03 | 1235.6146 | 10 | 4.7 | 618.8175 | 2 | 22.66 | 2 | F2:2729 | 21L153\_Guenther\_MPI\_AspN.raw |  | 9.4E8 |  |  | 1 | 0 | 1 | 0 | 0 | 488 | 497 |  |
| R.LQHSSLLANTLPTAPERHLEY.D | Y | 73.86 | 2389.2390 | 21 | 1.3 | 797.4213 | 3 | 26.27 | 2 | F2:3390 | 21L153\_Guenther\_MPI\_AspN.raw |  | 8.6E7 |  |  | 6 | 0 | 6 | 0 | 0 | 1036 | 1056 |  |
| K.AGAGLVVHNK.L | Y | 73.68 | 964.5454 | 10 | -0.5 | 483.2797 | 2 | 12.96 | 1 | F1:1007 | 21L153\_Guenther\_MPI\_Tr.raw | 4.9E7 |  |  |  | 6 | 6 | 0 | 0 | 0 | 1114 | 1123 |  |
| Y.DATGRIIASQ.D | Y | 73.38 | 1030.5406 | 10 | 1.2 | 516.2782 | 2 | 14.46 | 2 | F2:1241 | 21L153\_Guenther\_MPI\_AspN.raw |  | 1.37E9 |  |  | 7 | 0 | 7 | 0 | 0 | 1081 | 1090 |  |
| R.DAVGRLLAKITD.D | Y | 73.38 | 1270.7245 | 12 | 2.6 | 636.3712 | 2 | 29.94 | 2 | F2:4059 | 21L153\_Guenther\_MPI\_AspN.raw |  | 9.97E7 |  |  | 3 | 0 | 3 | 0 | 0 | 900 | 911 |  |
| R.VDGLGAQQK.I | Y | 73.23 | 914.4821 | 9 | 0.3 | 458.2485 | 2 | 13.11 | 1 | F1:1037 | 21L153\_Guenther\_MPI\_Tr.raw | 1.01E7 |  |  |  | 2 | 2 | 0 | 0 | 0 | 1235 | 1243 |  |
| W.GNTVEEIREPYYIEEQNLR.F | Y | 73.20 | 2351.1394 | 19 | 0.8 | 784.7211 | 3 | 26.78 | 1 | F1:3465 | 21L153\_Guenther\_MPI\_Tr.raw | 2.96E7 |  |  |  | 2 | 2 | 0 | 0 | 0 | 1275 | 1293 |  |
| E.DLGHAPLARV.D | Y | 73.01 | 1047.5825 | 10 | 0.3 | 524.7987 | 2 | 19.17 | 2 | F2:2088 | 21L153\_Guenther\_MPI\_AspN.raw |  | 3.9E9 |  |  | 11 | 0 | 11 | 0 | 0 | 1226 | 1235 |  |
| R.TQGQISTRSEY.D | Y | 72.99 | 1268.5997 | 11 | 1.1 | 635.3079 | 2 | 14.03 | 2 | F2:1157 | 21L153\_Guenther\_MPI\_AspN.raw |  | 4.18E6 |  |  | 2 | 0 | 2 | 0 | 0 | 1016 | 1026 |  |
| R.DSEGQVQR.R | Y | 72.77 | 917.4202 | 8 | -0.2 | 459.7173 | 2 | 11.76 | 1 | F1:833 | 21L153\_Guenther\_MPI\_Tr.raw | 4.41E5 |  |  |  | 3 | 3 | 0 | 0 | 0 | 510 | 517 |  |
| E.EQNLRFQGQYL.D | Y | 72.47 | 1394.6942 | 11 | 1.6 | 698.3555 | 2 | 26.39 | 2 | F2:3414 | 21L153\_Guenther\_MPI\_AspN.raw |  | 1.58E8 |  |  | 3 | 0 | 3 | 0 | 0 | 1289 | 1299 |  |
| R.FQGQYLDR.E | Y | 72.14 | 1025.4930 | 8 | -1.0 | 513.7533 | 2 | 16.66 | 1 | F1:1691 | 21L153\_Guenther\_MPI\_Tr.raw | 1.25E9 |  |  |  | 11 | 11 | 0 | 0 | 0 | 1294 | 1301 |  |
| Y.DAGHRMIEHQLATGLR.C | Y | 71.51 | 1803.9163 | 16 | 0.8 | 902.9661 | 2 | 17.38 | 2 | F2:1767 | 21L153\_Guenther\_MPI\_AspN.raw |  | 3.36E8 |  |  | 9 | 0 | 9 | 0 | 0 | 522 | 537 |  |
| L.DGSAKRYGY.D | Y | 71.50 | 1015.4723 | 9 | -1.0 | 508.7429 | 2 | 13.23 | 2 | F2:999 | 21L153\_Guenther\_MPI\_AspN.raw |  | 1.22E7 |  |  | 6 | 0 | 6 | 0 | 0 | 854 | 862 |  |
| R.FHYDC(+57.02)QGR.L | Y | 71.34 | 1081.4399 | 8 | 0.9 | 541.7277 | 2 | 13.17 | 1 | F1:1049 | 21L153\_Guenther\_MPI\_Tr.raw | 3.44E8 |  |  |  | 10 | 10 | 0 | 0 | 0 | 755 | 762 | Carbamidomethylation |
| N.DERQLLSAT.D | Y | 71.29 | 1031.5247 | 9 | 1.3 | 516.7703 | 2 | 19.95 | 2 | F2:2231 | 21L153\_Guenther\_MPI\_AspN.raw |  | 1.5E9 |  |  | 3 | 0 | 3 | 0 | 0 | 611 | 619 |  |
| Y.DELGNLTQTQLP.D | Y | 71.24 | 1327.6620 | 12 | 1.0 | 664.8389 | 2 | 29.19 | 2 | F2:3929 | 21L153\_Guenther\_MPI\_AspN.raw |  | 1.05E9 |  |  | 8 | 0 | 8 | 0 | 0 | 965 | 976 |  |
| A.DGRAEHFQRSSN(+.98)GQLQGYT.D | Y | 71.08 | 2150.9729 | 19 | 2.9 | 1076.4968 | 2 | 14.85 | 2 | F2:1315 | 21L153\_Guenther\_MPI\_AspN.raw |  | 6.42E8 |  |  | 6 | 0 | 6 | 0 | 0 | 770 | 788 | Deamidation (NQ) |
| T.DDGRSQYTY.D | Y | 71.06 | 1103.4519 | 9 | -0.7 | 552.7328 | 2 | 14.56 | 2 | F2:1260 | 21L153\_Guenther\_MPI\_AspN.raw |  | 4.54E8 |  |  | 6 | 0 | 6 | 0 | 0 | 911 | 919 |  |
| Q.SHPAPYGTGLSAVPAQPIAPIIHR.F | Y | 70.99 | 2449.3230 | 24 | 3.3 | 817.4510 | 3 | 26.59 | 1 | F1:3430 | 21L153\_Guenther\_MPI\_Tr.raw | 8.09E6 |  |  |  | 3 | 3 | 0 | 0 | 0 | 873 | 896 |  |
| A.N(+.98)TLPTAPERHLEY.D | Y | 70.98 | 1540.7521 | 13 | 0.5 | 771.3837 | 2 | 21.67 | 2 | F2:2545 | 21L153\_Guenther\_MPI\_AspN.raw |  | 1.42E7 |  |  | 3 | 0 | 3 | 0 | 0 | 1044 | 1056 | Deamidation (NQ) |
| D.PLGQVTQYR.H | Y | 70.39 | 1060.5665 | 9 | 0.6 | 531.2908 | 2 | 16.82 | 1 | F1:1721 | 21L153\_Guenther\_MPI\_Tr.raw | 2.44E7 |  |  |  | 3 | 3 | 0 | 0 | 0 | 685 | 693 |  |
| E.DLGHAPLARVDGLGAQQKIRYYHN.D | Y | 70.29 | 2691.3994 | 24 | 3.9 | 898.1439 | 3 | 23.24 | 2 | F2:2828 | 21L153\_Guenther\_MPI\_AspN.raw |  | 2.29E8 |  |  | 9 | 0 | 9 | 0 | 0 | 1226 | 1249 |  |
| Y.DAAANLLDGPK.A | Y | 70.21 | 1083.5560 | 11 | -0.8 | 542.7849 | 2 | 23.10 | 1 | F1:2806 | 21L153\_Guenther\_MPI\_Tr.raw | 4.34E6 |  |  |  | 3 | 3 | 0 | 0 | 0 | 1103 | 1113 |  |
| R.YDVLGR.R | Y | 69.99 | 721.3759 | 6 | 0.1 | 361.6953 | 2 | 17.42 | 1 | F1:1829 | 21L153\_Guenther\_MPI\_Tr.raw | 1.42E9 |  |  |  | 9 | 9 | 0 | 0 | 0 | 1179 | 1184 |  |
| R.FAYDAGHR.M | Y | 69.83 | 935.4249 | 8 | -0.8 | 468.7194 | 2 | 13.44 | 1 | F1:1104 | 21L153\_Guenther\_MPI\_Tr.raw | 2.29E9 |  |  |  | 19 | 19 | 0 | 0 | 0 | 519 | 526 |  |
| R.YDAFGR.M | Y | 69.27 | 727.3289 | 6 | -2.2 | 364.6709 | 2 | 15.83 | 1 | F1:1541 | 21L153\_Guenther\_MPI\_Tr.raw | 1.04E9 |  |  |  | 9 | 9 | 0 | 0 | 0 | 1134 | 1139 |  |
| R.LLALTN(+.98)ENGESYR.F | Y | 69.18 | 1479.7205 | 13 | 3.3 | 740.8699 | 2 | 23.77 | 1 | F1:2931 | 21L153\_Guenther\_MPI\_Tr.raw | 1.49E9 |  |  |  | 1 | 1 | 0 | 0 | 0 | 826 | 838 | Deamidation (NQ) |
| R.LYYGSGHLHQIN.L | Y | 68.83 | 1400.6837 | 12 | -1.3 | 701.3482 | 2 | 17.21 | 1 | F1:1790 | 21L153\_Guenther\_MPI\_Tr.raw | 4.38E6 |  |  |  | 3 | 3 | 0 | 0 | 0 | 984 | 995 |  |
| C.SGYPTRFEY.D | Y | 68.70 | 1118.5032 | 9 | 0.9 | 560.2593 | 2 | 23.98 | 2 | F2:2965 | 21L153\_Guenther\_MPI\_AspN.raw |  | 2.77E7 |  |  | 3 | 0 | 3 | 0 | 0 | 728 | 736 |  |
| R.LLAKITDDGR.S | Y | 68.59 | 1100.6189 | 10 | -1.0 | 551.3162 | 2 | 15.50 | 1 | F1:1478 | 21L153\_Guenther\_MPI\_Tr.raw | 7.65E6 |  |  |  | 5 | 5 | 0 | 0 | 0 | 905 | 914 |  |
| L.DGPKAGAGLVVHN(+.98)KLLTYQ.D | Y | 68.56 | 1981.0632 | 19 | 3.2 | 496.2747 | 4 | 24.96 | 2 | F2:3150 | 21L153\_Guenther\_MPI\_AspN.raw |  | 1.98E6 |  |  | 2 | 0 | 2 | 0 | 0 | 1110 | 1128 | Deamidation (NQ) |
| Y.DPDIGRFTTP.D | Y | 68.53 | 1117.5404 | 10 | 1.3 | 559.7782 | 2 | 24.39 | 2 | F2:3043 | 21L153\_Guenther\_MPI\_AspN.raw |  | 1.47E8 |  |  | 3 | 0 | 3 | 0 | 0 | 1314 | 1323 |  |
| N.DN(+.98)RLHLEY.D | Y | 68.26 | 1059.4985 | 8 | 1.4 | 530.7573 | 2 | 22.00 | 2 | F2:2607 | 21L153\_Guenther\_MPI\_AspN.raw |  | 1.56E7 |  |  | 6 | 0 | 6 | 0 | 0 | 442 | 449 | Deamidation (NQ) |
| S.DNLVGLAERHATGQHRQLFHY.D | Y | 68.26 | 2461.2363 | 21 | 3.1 | 1231.6293 | 2 | 25.39 | 2 | F2:3228 | 21L153\_Guenther\_MPI\_AspN.raw |  | 5.35E9 |  |  | 10 | 0 | 10 | 0 | 0 | 1060 | 1080 |  |
| T.DTPSATPEEGPGFL.D | Y | 67.90 | 1416.6409 | 14 | 1.9 | 709.3290 | 2 | 28.79 | 2 | F2:3859 | 21L153\_Guenther\_MPI\_AspN.raw |  | 1.13E7 |  |  | 2 | 0 | 2 | 0 | 0 | 141 | 154 |  |
| A.NTLPTAPERHLEY.D | Y | 67.75 | 1539.7681 | 13 | 0.9 | 770.8920 | 2 | 20.27 | 2 | F2:2289 | 21L153\_Guenther\_MPI\_AspN.raw |  | 4.79E6 |  |  | 3 | 0 | 3 | 0 | 0 | 1044 | 1056 |  |
| Y.DAAANLLDGPKAGAGLVVHNKLLTYQ.D | Y | 67.69 | 2648.4285 | 26 | 1.9 | 883.8185 | 3 | 28.20 | 2 | F2:3749 | 21L153\_Guenther\_MPI\_AspN.raw |  | 4.33E6 |  |  | 2 | 0 | 2 | 0 | 0 | 1103 | 1128 |  |
| T.DNLNQTWAF.E | Y | 67.44 | 1107.4985 | 9 | 0.3 | 554.7567 | 2 | 32.75 | 2 | F2:4554 | 21L153\_Guenther\_MPI\_AspN.raw |  | 9.2E6 |  |  | 3 | 0 | 3 | 0 | 0 | 599 | 607 |  |
| R.WNSSGQLLEHLD.C | Y | 67.39 | 1397.6575 | 12 | 2.3 | 699.8376 | 2 | 28.35 | 1 | F1:3753 | 21L153\_Guenther\_MPI\_Tr.raw | 9.13E6 |  |  |  | 2 | 2 | 0 | 0 | 0 | 715 | 726 |  |
| S.GHLHQINLDGQVVSDFQR.D | Y | 67.34 | 2062.0344 | 18 | 0.5 | 688.3524 | 3 | 25.40 | 1 | F1:3211 | 21L153\_Guenther\_MPI\_Tr.raw | 2.38E6 |  |  |  | 3 | 3 | 0 | 0 | 0 | 989 | 1006 |  |
| K.AGAGLVVHNKLLTYQDK.R | Y | 67.20 | 1826.0050 | 17 | -1.2 | 609.6749 | 3 | 22.42 | 1 | F1:2685 | 21L153\_Guenther\_MPI\_Tr.raw | 4.97E6 |  |  |  | 3 | 3 | 0 | 0 | 0 | 1114 | 1130 |  |
| L.DGQVVSDFQR.D | Y | 67.10 | 1149.5414 | 10 | 0.2 | 575.7781 | 2 | 15.95 | 2 | F2:1511 | 21L153\_Guenther\_MPI\_AspN.raw |  | 2.61E6 |  |  | 3 | 0 | 3 | 0 | 0 | 997 | 1006 |  |
| R.PTVASPAPGSQTK.E | Y | 66.93 | 1239.6459 | 13 | 0.4 | 620.8304 | 2 | 12.81 | 1 | F1:979 | 21L153\_Guenther\_MPI\_Tr.raw | 2.84E6 |  |  |  | 3 | 3 | 0 | 0 | 0 | 167 | 179 |  |
| H.SSLLANTLPTAPER.H | Y | 66.65 | 1468.7886 | 14 | 0.7 | 735.4021 | 2 | 26.34 | 1 | F1:3383 | 21L153\_Guenther\_MPI\_Tr.raw | 1.1E7 |  |  |  | 3 | 3 | 0 | 0 | 0 | 1039 | 1052 |  |
| I.DALGEHTRFHYD.C | Y | 66.51 | 1459.6479 | 12 | 0.7 | 730.8318 | 2 | 17.23 | 2 | F2:1736 | 21L153\_Guenther\_MPI\_AspN.raw |  | 1.82E7 |  |  | 3 | 0 | 3 | 0 | 0 | 747 | 758 |  |
| Q.DLDGSAKRYGY.D | Y | 66.11 | 1243.5833 | 11 | 0.4 | 622.7991 | 2 | 19.56 | 2 | F2:2160 | 21L153\_Guenther\_MPI\_AspN.raw |  | 1.77E8 |  |  | 9 | 0 | 9 | 0 | 0 | 852 | 862 |  |
| R.FQGQYLDRETGLHFNTFR.F | Y | 65.89 | 2228.0762 | 18 | 1.7 | 743.7006 | 3 | 27.23 | 1 | F1:3547 | 21L153\_Guenther\_MPI\_Tr.raw | 2.84E7 |  |  |  | 4 | 4 | 0 | 0 | 0 | 1294 | 1311 |  |
| W.NSSGQLLEHL.D | Y | 65.89 | 1096.5513 | 10 | 1.3 | 549.2836 | 2 | 26.38 | 2 | F2:3411 | 21L153\_Guenther\_MPI\_AspN.raw |  | 6.44E6 |  |  | 2 | 0 | 2 | 0 | 0 | 716 | 725 |  |
| K.LLTYQDKR.Y | Y | 65.86 | 1035.5713 | 8 | -0.7 | 518.7925 | 2 | 13.82 | 1 | F1:1170 | 21L153\_Guenther\_MPI\_Tr.raw | 2.03E8 |  |  |  | 6 | 6 | 0 | 0 | 0 | 1124 | 1131 |  |
| S.EYDSQGNLLGETR.F | Y | 65.22 | 1480.6793 | 13 | -0.6 | 741.3465 | 2 | 20.84 | 1 | F1:2410 | 21L153\_Guenther\_MPI\_Tr.raw | 2.75E6 |  |  |  | 3 | 3 | 0 | 0 | 0 | 1190 | 1202 |  |
| R.YGYDLLN(+.98)NVVELQSHPAPYGTGLSAVPAQPIAPIIHR.F | Y | 65.14 | 3971.0581 | 37 | 2.1 | 993.7739 | 4 | 32.74 | 1 | F1:4539 | 21L153\_Guenther\_MPI\_Tr.raw | 1.4E8 |  |  |  | 1 | 1 | 0 | 0 | 0 | 860 | 896 | Deamidation (NQ) |
| R.DGLLGAGWSLPYEISVQIEPHPEGGERLLYI.D | Y | 64.43 | 3407.7400 | 31 | 3.3 | 1136.9243 | 3 | 34.66 | 2 | F2:4880 | 21L153\_Guenther\_MPI\_AspN.raw |  | 1.3E6 |  |  | 4 | 0 | 4 | 0 | 0 | 345 | 375 |  |
| R.NQQTSLYVYEDLGHAPLAR.V | Y | 63.34 | 2174.0757 | 19 | 4.6 | 725.7025 | 3 | 27.99 | 1 | F1:3688 | 21L153\_Guenther\_MPI\_Tr.raw | 1.25E9 |  |  |  | 5 | 5 | 0 | 0 | 0 | 1216 | 1234 |  |
| D.PLGQVTQYRH.D | Y | 63.31 | 1197.6255 | 10 | -1.4 | 599.8192 | 2 | 14.29 | 2 | F2:1208 | 21L153\_Guenther\_MPI\_AspN.raw |  | 7.68E7 |  |  | 9 | 0 | 9 | 0 | 0 | 685 | 694 |  |
| R.NDN(+.98)RLHLEYDSSGR.L | Y | 62.99 | 1675.7550 | 14 | -1.5 | 559.5914 | 3 | 16.54 | 1 | F1:1670 | 21L153\_Guenther\_MPI\_Tr.raw | 8.36E5 |  |  |  | 3 | 3 | 0 | 0 | 0 | 441 | 454 | Deamidation (NQ) |
| D.DGRSQYTY.D | Y | 62.96 | 988.4250 | 8 | 1.3 | 495.2204 | 2 | 14.44 | 2 | F2:1238 | 21L153\_Guenther\_MPI\_AspN.raw |  | 7.1E7 |  |  | 3 | 0 | 3 | 0 | 0 | 912 | 919 |  |
| Y.DAAGDLAQVR.D | Y | 62.63 | 1014.5094 | 10 | 2.3 | 508.2632 | 2 | 17.06 | 1 | F1:1763 | 21L153\_Guenther\_MPI\_Tr.raw | 3.74E6 |  |  |  | 3 | 3 | 0 | 0 | 0 | 500 | 509 |  |
| W.TQFADQQWR.V | Y | 62.53 | 1178.5469 | 9 | -1.5 | 590.2798 | 2 | 20.37 | 1 | F1:2332 | 21L153\_Guenther\_MPI\_Tr.raw | 1.62E7 |  |  |  | 3 | 3 | 0 | 0 | 0 | 543 | 551 |  |
| P.DKRWLNRLYYGSGHLHQINL.D | Y | 62.48 | 2482.2981 | 20 | 3.4 | 1242.1605 | 2 | 27.41 | 2 | F2:3599 | 21L153\_Guenther\_MPI\_AspN.raw |  | 1.89E8 |  |  | 3 | 0 | 3 | 0 | 0 | 977 | 996 |  |
| Y.EDLGHAPLARV.D | Y | 62.42 | 1176.6251 | 11 | 1.3 | 589.3206 | 2 | 19.10 | 2 | F2:2075 | 21L153\_Guenther\_MPI\_AspN.raw |  | 9.16E7 |  |  | 5 | 0 | 5 | 0 | 0 | 1225 | 1235 |  |
| Y.DPLN(+.98)QLTAASFT.D | Y | 62.10 | 1277.6139 | 12 | 2.5 | 639.8158 | 2 | 29.55 | 2 | F2:3989 | 21L153\_Guenther\_MPI\_AspN.raw |  | 1.3E7 |  |  | 3 | 0 | 3 | 0 | 0 | 920 | 931 | Deamidation (NQ) |
| Y.DAEHRLIEVR.N | Y | 61.94 | 1236.6575 | 10 | 0.5 | 413.2267 | 3 | 15.50 | 2 | F2:1433 | 21L153\_Guenther\_MPI\_AspN.raw |  | 3.75E7 |  |  | 6 | 0 | 6 | 0 | 0 | 1156 | 1165 |  |
| Y.DETGNLIAET.D | Y | 61.89 | 1061.4877 | 10 | 2.6 | 531.7525 | 2 | 23.09 | 2 | F2:2802 | 21L153\_Guenther\_MPI\_AspN.raw |  | 5.19E8 |  |  | 3 | 0 | 3 | 0 | 0 | 631 | 640 |  |
| Y.DAEHRLIEVRNQTSSG.E | Y | 61.87 | 1810.8921 | 16 | 2.0 | 906.4551 | 2 | 15.31 | 2 | F2:1400 | 21L153\_Guenther\_MPI\_AspN.raw |  | 1.84E7 |  |  | 6 | 0 | 6 | 0 | 0 | 1156 | 1171 |  |
| R.YAYSYDETGNLIAETDPLGHSHSTL.W | Y | 61.74 | 2753.2456 | 25 | 2.1 | 918.7578 | 3 | 31.86 | 1 | F1:4384 | 21L153\_Guenther\_MPI\_Tr.raw | 4.01E6 |  |  |  | 2 | 2 | 0 | 0 | 0 | 626 | 650 |  |
| T.DPLGHSHSTLWL.E | Y | 61.45 | 1361.6727 | 12 | 1.1 | 681.8444 | 2 | 30.09 | 2 | F2:4087 | 21L153\_Guenther\_MPI\_AspN.raw |  | 5.03E7 |  |  | 6 | 0 | 6 | 0 | 0 | 641 | 652 |  |
| R.DAVGRLLAKIT.D | Y | 61.26 | 1155.6975 | 11 | 3.7 | 578.8582 | 2 | 30.08 | 2 | F2:4085 | 21L153\_Guenther\_MPI\_AspN.raw | 8.41E6 | 2.68E9 |  |  | 9 | 2 | 7 | 0 | 0 | 900 | 910 |  |
| D.PLGGRYAYSY.D | Y | 61.10 | 1145.5505 | 10 | 1.0 | 573.7831 | 2 | 21.98 | 2 | F2:2603 | 21L153\_Guenther\_MPI\_AspN.raw |  | 3.82E7 |  |  | 3 | 0 | 3 | 0 | 0 | 621 | 630 |  |
| A.LGEHTRFHY.D | Y | 60.86 | 1158.5570 | 9 | -0.8 | 580.2853 | 2 | 17.93 | 2 | F2:1866 | 21L153\_Guenther\_MPI\_AspN.raw |  | 2.15E7 |  |  | 3 | 0 | 3 | 0 | 0 | 749 | 757 |  |
| R.HDAHGQ(+.98)VVEIIDAAGR.S | Y | 60.77 | 1687.8278 | 16 | 4.0 | 563.6188 | 3 | 23.43 | 1 | F1:2871 | 21L153\_Guenther\_MPI\_Tr.raw | 3.63E7 |  |  |  | 1 | 1 | 0 | 0 | 0 | 694 | 709 | Deamidation (NQ) |
| D.SDNLVGLAER.H | Y | 60.31 | 1072.5513 | 10 | -1.8 | 537.2819 | 2 | 23.13 | 1 | F1:2813 | 21L153\_Guenther\_MPI\_Tr.raw | 3.78E6 |  |  |  | 2 | 2 | 0 | 0 | 0 | 1059 | 1068 |  |
| R.WNSQLQIVEYT.D | Y | 60.29 | 1379.6721 | 11 | -0.9 | 690.8427 | 2 | 30.95 | 2 | F2:4239 | 21L153\_Guenther\_MPI\_AspN.raw |  | 2.45E6 |  |  | 1 | 0 | 1 | 0 | 0 | 588 | 598 |  |
| I.DM(+15.99)GSIALGAGAFSPGEGLSVRRNE.N | Y | 60.18 | 2406.1597 | 24 | -1.7 | 803.0591 | 3 | 27.62 | 2 | F2:3639 | 21L153\_Guenther\_MPI\_AspN.raw |  | 2.79E6 |  |  | 1 | 0 | 1 | 0 | 0 | 383 | 406 | Oxidation (M) |
| D.PAGESWHLRY.D | Y | 60.15 | 1214.5833 | 10 | 0.6 | 405.8686 | 3 | 23.85 | 2 | F2:2940 | 21L153\_Guenther\_MPI\_AspN.raw |  | 3.33E7 |  |  | 3 | 0 | 3 | 0 | 0 | 664 | 673 |  |
| Y.DRRGN(+.98)VKAII.D | Y | 59.96 | 1141.6567 | 10 | 0.5 | 571.8359 | 2 | 18.36 | 2 | F2:1945 | 21L153\_Guenther\_MPI\_AspN.raw |  | 1.95E7 |  |  | 5 | 0 | 5 | 0 | 0 | 737 | 746 | Deamidation (NQ) |
| H.LHQINLDGQVVSDFQR.D | Y | 59.81 | 1867.9540 | 16 | -0.2 | 623.6584 | 3 | 26.89 | 1 | F1:3484 | 21L153\_Guenther\_MPI\_Tr.raw | 5.96E6 |  |  |  | 1 | 1 | 0 | 0 | 0 | 991 | 1006 |  |
| N.RLSEQQDLDGSAK.R | Y | 59.77 | 1445.7109 | 13 | 1.0 | 723.8635 | 2 | 13.18 | 1 | F1:1053 | 21L153\_Guenther\_MPI\_Tr.raw | 3.9E6 |  |  |  | 3 | 3 | 0 | 0 | 0 | 846 | 858 |  |
| L.GQLLEEHTVAGSLIHR.Y | Y | 59.75 | 1758.9376 | 16 | -1.4 | 587.3190 | 3 | 23.94 | 1 | F1:2955 | 21L153\_Guenther\_MPI\_Tr.raw | 8.99E6 |  |  |  | 2 | 2 | 0 | 0 | 0 | 948 | 963 |  |
| L.EHWSLPQTLT.D | Y | 59.48 | 1210.5983 | 10 | 0.9 | 606.3069 | 2 | 28.38 | 2 | F2:3782 | 21L153\_Guenther\_MPI\_AspN.raw |  | 2.05E7 |  |  | 2 | 0 | 2 | 0 | 0 | 653 | 662 |  |
| Y.DAGHRM(+15.99)IEHQLATGLR.C | Y | 59.33 | 1819.9111 | 16 | -1.0 | 607.6437 | 3 | 14.78 | 2 | F2:1303 | 21L153\_Guenther\_MPI\_AspN.raw |  | 1.03E8 |  |  | 6 | 0 | 6 | 0 | 0 | 522 | 537 | Oxidation (M) |
| K.SEYDSQGNLLG.E | Y | 59.20 | 1181.5200 | 11 | 0.7 | 591.7677 | 2 | 23.11 | 1 | F1:2809 | 21L153\_Guenther\_MPI\_Tr.raw | 2.31E6 |  |  |  | 3 | 3 | 0 | 0 | 0 | 1189 | 1199 |  |
| R.KMRWNSSGQLLEHL.D | Y | 58.85 | 1697.8672 | 14 | 1.0 | 566.9636 | 3 | 26.73 | 2 | F2:3475 | 21L153\_Guenther\_MPI\_AspN.raw |  | 2.21E7 |  |  | 3 | 0 | 3 | 0 | 0 | 712 | 725 |  |
| R.LYPDQQR.E | Y | 58.29 | 918.4559 | 7 | -0.8 | 460.2349 | 2 | 13.26 | 1 | F1:1070 | 21L153\_Guenther\_MPI\_Tr.raw | 1.29E8 |  |  |  | 5 | 5 | 0 | 0 | 0 | 485 | 491 |  |
| H.PVHAATGAK.V | Y | 58.29 | 850.4661 | 9 | -0.1 | 426.2403 | 2 | 11.51 | 1 | F1:803 | 21L153\_Guenther\_MPI\_Tr.raw | 7.1E4 |  |  |  | 2 | 2 | 0 | 0 | 0 | 305 | 313 |  |
| T.DPLGGRYA.Y | Y | 58.04 | 847.4188 | 8 | 0.6 | 424.7169 | 2 | 16.36 | 2 | F2:1585 | 21L153\_Guenther\_MPI\_AspN.raw |  | 2.74E6 |  |  | 3 | 0 | 3 | 0 | 0 | 620 | 627 |  |
| R.QLLSATDPLGGR.Y | Y | 57.66 | 1226.6619 | 12 | 3.4 | 614.3403 | 2 | 25.15 | 1 | F1:3163 | 21L153\_Guenther\_MPI\_Tr.raw | 1.12E8 |  |  |  | 6 | 6 | 0 | 0 | 0 | 614 | 625 |  |
| L.DRETGLHFNTFR.F | Y | 57.66 | 1491.7218 | 12 | 1.1 | 498.2484 | 3 | 22.03 | 2 | F2:2613 | 21L153\_Guenther\_MPI\_AspN.raw |  | 6.99E6 |  |  | 2 | 0 | 2 | 0 | 0 | 1300 | 1311 |  |
| M.IEHQLATGLR.C | Y | 57.56 | 1136.6301 | 10 | -1.3 | 569.3216 | 2 | 17.94 | 1 | F1:1920 | 21L153\_Guenther\_MPI\_Tr.raw | 8.62E6 |  |  |  | 3 | 3 | 0 | 0 | 0 | 528 | 537 |  |
| K.ETFAYDAAANLLD.G | Y | 57.14 | 1412.6459 | 13 | 1.2 | 707.3311 | 2 | 33.30 | 1 | F1:4636 | 21L153\_Guenther\_MPI\_Tr.raw | 2.19E6 |  |  |  | 3 | 3 | 0 | 0 | 0 | 1098 | 1110 |  |
| I.DM(+15.99)GSIALGAGAFSPGEGLSVRRNENGQLLIESS.D | Y | 57.13 | 3347.6416 | 33 | 3.1 | 1116.8912 | 3 | 31.20 | 2 | F2:4283 | 21L153\_Guenther\_MPI\_AspN.raw |  | 4.83E6 |  |  | 1 | 0 | 1 | 0 | 0 | 383 | 415 | Oxidation (M) |
| Q.LFHYDATGR.I | Y | 57.09 | 1078.5195 | 9 | 0.3 | 540.2672 | 2 | 19.51 | 1 | F1:2190 | 21L153\_Guenther\_MPI\_Tr.raw | 3.39E6 |  |  |  | 3 | 3 | 0 | 0 | 0 | 1077 | 1085 |  |
| L.DFTLPGLM(+15.99)PIEWQRFY.N | Y | 57.08 | 2027.9814 | 16 | 3.1 | 1015.0011 | 2 | 34.53 | 2 | F2:4860 | 21L153\_Guenther\_MPI\_AspN.raw |  | 2.72E5 |  |  | 1 | 0 | 1 | 0 | 0 | 322 | 337 | Oxidation (M) |
| E.EIREPYYIEEQNLRFQGQYL.D | Y | 57.05 | 2587.2705 | 20 | 1.1 | 863.4318 | 3 | 31.03 | 2 | F2:4253 | 21L153\_Guenther\_MPI\_AspN.raw |  | 3.86E7 |  |  | 3 | 0 | 3 | 0 | 0 | 1280 | 1299 |  |
| S.LLANTLPTAPER.H | Y | 56.88 | 1294.7245 | 12 | -1.8 | 648.3683 | 2 | 23.08 | 1 | F1:2802 | 21L153\_Guenther\_MPI\_Tr.raw | 3.53E6 |  |  |  | 3 | 3 | 0 | 0 | 0 | 1041 | 1052 |  |
| R.VDGLGAQQKIR.Y | Y | 56.86 | 1183.6672 | 11 | 0.2 | 592.8410 | 2 | 13.95 | 1 | F1:1196 | 21L153\_Guenther\_MPI\_Tr.raw | 2E6 |  |  |  | 1 | 1 | 0 | 0 | 0 | 1235 | 1245 |  |
| F.AYDAEHR.L | Y | 56.78 | 860.3776 | 7 | 0.5 | 431.1963 | 2 | 13.71 | 1 | F1:1151 | 21L153\_Guenther\_MPI\_Tr.raw | 1.26E7 |  |  |  | 3 | 3 | 0 | 0 | 0 | 1154 | 1160 |  |
| Y.TTLYQYNR.R | Y | 56.00 | 1057.5193 | 8 | -2.3 | 529.7657 | 2 | 15.93 | 1 | F1:1561 | 21L153\_Guenther\_MPI\_Tr.raw | 1.58E7 |  |  |  | 3 | 3 | 0 | 0 | 0 | 794 | 801 |  |
| R.YGYDLLNNVVELQSHPAPYGTGLSAVPAQPIAPIIHR.F | Y | 55.78 | 3970.0740 | 37 | 3.1 | 1324.3694 | 3 | 34.32 | 1 | F1:4812 | 21L153\_Guenther\_MPI\_Tr.raw | 4.16E7 |  |  |  | 1 | 1 | 0 | 0 | 0 | 860 | 896 |  |
| L.GAGAFSPGEGLSVR.R | Y | 55.75 | 1303.6520 | 14 | 2.0 | 652.8346 | 2 | 24.20 | 1 | F1:3001 | 21L153\_Guenther\_MPI\_Tr.raw | 3.77E6 |  |  |  | 2 | 2 | 0 | 0 | 0 | 390 | 403 |  |
| R.LSLLGDR.N | Y | 55.54 | 772.4443 | 7 | 0.4 | 387.2296 | 2 | 25.61 | 1 | F1:3249 | 21L153\_Guenther\_MPI\_Tr.raw | 1.62E9 |  |  |  | 5 | 5 | 0 | 0 | 0 | 434 | 440 |  |
| L.DRETGLHFNTF.R | Y | 55.54 | 1335.6207 | 11 | 1.4 | 668.8185 | 2 | 26.60 | 2 | F2:3451 | 21L153\_Guenther\_MPI\_AspN.raw |  | 9.81E6 |  |  | 3 | 0 | 3 | 0 | 0 | 1300 | 1310 |  |
| E.TGLHFNTFR.F | Y | 55.11 | 1091.5513 | 9 | 0.8 | 546.7833 | 2 | 23.69 | 1 | F1:2917 | 21L153\_Guenther\_MPI\_Tr.raw | 1.05E7 |  |  |  | 3 | 3 | 0 | 0 | 0 | 1303 | 1311 |  |
| R.YYHNDLN(+.98)GLPQQL.C | Y | 55.09 | 1574.7365 | 13 | 5.0 | 788.3795 | 2 | 27.32 | 1 | F1:3564 | 21L153\_Guenther\_MPI\_Tr.raw | 1.06E7 |  |  |  | 1 | 1 | 0 | 0 | 0 | 1246 | 1258 | Deamidation (NQ) |
| D.SQGNLLGETR.F | Y | 54.76 | 1073.5465 | 10 | -2.1 | 537.7794 | 2 | 16.64 | 1 | F1:1688 | 21L153\_Guenther\_MPI\_Tr.raw | 2.03E6 |  |  |  | 3 | 3 | 0 | 0 | 0 | 1193 | 1202 |  |
| Y.DAFGRMIEKR.S | Y | 54.60 | 1221.6288 | 10 | 0.9 | 408.2172 | 3 | 17.35 | 2 | F2:1759 | 21L153\_Guenther\_MPI\_AspN.raw |  | 2.03E7 |  |  | 3 | 0 | 3 | 0 | 0 | 1135 | 1144 |  |
| I.DMGSIALGAGAFSPGEGLSVRRNEN(+.98)GQLLIESS.D | Y | 54.58 | 3332.6306 | 33 | 1.4 | 1111.8857 | 3 | 32.48 | 2 | F2:4506 | 21L153\_Guenther\_MPI\_AspN.raw |  | 2.2E7 |  |  | 2 | 0 | 2 | 0 | 0 | 383 | 415 | Deamidation (NQ) |
| A.GAFSPGEGLSVR.R | Y | 54.01 | 1175.5934 | 12 | 1.7 | 588.8050 | 2 | 23.66 | 1 | F1:2911 | 21L153\_Guenther\_MPI\_Tr.raw | 4.65E6 |  |  |  | 1 | 1 | 0 | 0 | 0 | 392 | 403 |  |
| K.LLTYQDK.R | Y | 53.84 | 879.4702 | 7 | 0.9 | 440.7427 | 2 | 15.29 | 1 | F1:1439 | 21L153\_Guenther\_MPI\_Tr.raw | 1.13E9 |  |  |  | 3 | 3 | 0 | 0 | 0 | 1124 | 1130 |  |
| R.FAWDAGNR.L | Y | 53.83 | 935.4249 | 8 | -3.2 | 468.7183 | 2 | 21.80 | 1 | F1:2573 | 21L153\_Guenther\_MPI\_Tr.raw | 5.53E8 |  |  |  | 5 | 5 | 0 | 0 | 0 | 839 | 846 |  |
| H.DAHGQVVEII.D | Y | 53.82 | 1079.5610 | 10 | 1.4 | 540.7886 | 2 | 26.33 | 2 | F2:3401 | 21L153\_Guenther\_MPI\_AspN.raw |  | 1.8E9 |  |  | 5 | 0 | 5 | 0 | 0 | 695 | 704 |  |
| R.IIASQDGLQGQKETFAYDAAANLLDGPK.A | Y | 53.80 | 2933.4771 | 28 | 2.0 | 978.8349 | 3 | 32.75 | 1 | F1:4542 | 21L153\_Guenther\_MPI\_Tr.raw | 7.41E6 |  |  |  | 2 | 2 | 0 | 0 | 0 | 1086 | 1113 |  |
| I.DM(+15.99)GSIALGAGAFSPGEGLSVRRN(+.98)ENGQLLIESS.D | Y | 53.55 | 3348.6255 | 33 | 1.5 | 1117.2174 | 3 | 30.93 | 2 | F2:4236 | 21L153\_Guenther\_MPI\_AspN.raw |  | 4.87E6 |  |  | 3 | 0 | 3 | 0 | 0 | 383 | 415 | Oxidation (M); Deamidation (NQ) |
| L.GHAPLARV.D | Y | 53.32 | 819.4715 | 8 | 0.3 | 410.7431 | 2 | 19.23 | 2 | F2:2101 | 21L153\_Guenther\_MPI\_AspN.raw |  | 5.26E7 |  |  | 3 | 0 | 3 | 0 | 0 | 1228 | 1235 |  |
| F.SPGEGLSVR.R | Y | 53.22 | 900.4664 | 9 | 0.3 | 451.2406 | 2 | 14.88 | 1 | F1:1361 | 21L153\_Guenther\_MPI\_Tr.raw | 1.19E7 |  |  |  | 3 | 3 | 0 | 0 | 0 | 395 | 403 |  |
| P.YYIEEQNLR.F | Y | 53.17 | 1226.5931 | 9 | 0.1 | 614.3039 | 2 | 21.64 | 1 | F1:2543 | 21L153\_Guenther\_MPI\_Tr.raw | 1.49E6 |  |  |  | 2 | 2 | 0 | 0 | 0 | 1285 | 1293 |  |
| Q.VVEIIDAAGR.S | Y | 53.03 | 1041.5818 | 10 | -3.0 | 521.7966 | 2 | 22.18 | 1 | F1:2640 | 21L153\_Guenther\_MPI\_Tr.raw | 4.16E6 |  |  |  | 3 | 3 | 0 | 0 | 0 | 700 | 709 |  |
| D.GPKAGAGLVVHNKLLTYQ.D | Y | 52.98 | 1865.0522 | 18 | 0.8 | 622.6918 | 3 | 22.78 | 2 | F2:2751 | 21L153\_Guenther\_MPI\_AspN.raw |  | 1.3E7 |  |  | 3 | 0 | 3 | 0 | 0 | 1111 | 1128 |  |
| K.ITDDGR.S | Y | 52.61 | 675.3187 | 6 | 0.4 | 338.6668 | 2 | 11.78 | 1 | F1:835 | 21L153\_Guenther\_MPI\_Tr.raw | 2.78E5 |  |  |  | 3 | 3 | 0 | 0 | 0 | 909 | 914 |  |
| F.AYDAGHR.M | Y | 52.51 | 788.3565 | 7 | 0.0 | 395.1855 | 2 | 13.47 | 1 | F1:1112 | 21L153\_Guenther\_MPI\_Tr.raw | 1.07E7 |  |  |  | 1 | 1 | 0 | 0 | 0 | 520 | 526 |  |
| S.DGIYRLF.E | Y | 52.16 | 882.4599 | 7 | 1.4 | 442.2379 | 2 | 31.70 | 2 | F2:4373 | 21L153\_Guenther\_MPI\_AspN.raw |  | 8.93E7 |  |  | 3 | 0 | 3 | 0 | 0 | 416 | 422 |  |
| I.DMGSIALGAGAFSPGEGLSVRRNENGQLLIESS.D | Y | 52.02 | 3331.6465 | 33 | 0.3 | 1111.5564 | 3 | 34.99 | 2 | F2:4926 | 21L153\_Guenther\_MPI\_AspN.raw |  | 4.28E5 |  |  | 2 | 0 | 2 | 0 | 0 | 383 | 415 |  |
| Y.DC(+57.02)QGRLLSSQLA.D | Y | 51.92 | 1346.6613 | 12 | 4.4 | 674.3409 | 2 | 23.12 | 2 | F2:2809 | 21L153\_Guenther\_MPI\_AspN.raw |  | 6.34E8 |  |  | 1 | 0 | 1 | 0 | 0 | 758 | 769 | Carbamidomethylation |
| R.TQGQISTR.S | Y | 51.90 | 889.4617 | 8 | 0.9 | 445.7386 | 2 | 12.15 | 1 | F1:883 | 21L153\_Guenther\_MPI\_Tr.raw | 8.94E5 |  |  |  | 3 | 3 | 0 | 0 | 0 | 1016 | 1023 |  |
| N.DLN(+.98)GLPQQLC(+57.02)EP.D | Y | 51.83 | 1383.6340 | 12 | 3.2 | 692.8265 | 2 | 27.47 | 2 | F2:3611 | 21L153\_Guenther\_MPI\_AspN.raw |  | 5.28E6 |  |  | 2 | 0 | 2 | 0 | 0 | 1250 | 1261 | Deamidation (NQ); Carbamidomethylation |
| I.DAHGRQVRF.D | Y | 51.82 | 1084.5526 | 9 | 0.8 | 362.5251 | 3 | 13.73 | 2 | F2:1097 | 21L153\_Guenther\_MPI\_AspN.raw |  | 1.64E9 |  |  | 7 | 0 | 7 | 0 | 0 | 810 | 818 |  |
| G.ESWHLRY.D | Y | 51.78 | 989.4719 | 7 | 1.6 | 495.7440 | 2 | 23.12 | 2 | F2:2810 | 21L153\_Guenther\_MPI\_AspN.raw |  | 5E7 |  |  | 6 | 0 | 6 | 0 | 0 | 667 | 673 |  |
| Y.DPRGNC(+57.02)IAEVDPLGQVTQYRH.D | Y | 51.53 | 2424.1604 | 21 | 2.9 | 809.0631 | 3 | 26.63 | 2 | F2:3456 | 21L153\_Guenther\_MPI\_AspN.raw |  | 4.53E8 |  |  | 7 | 0 | 7 | 0 | 0 | 674 | 694 | Carbamidomethylation |
| T.GLHFNTFR.F | Y | 51.52 | 990.5035 | 8 | -1.3 | 496.2584 | 2 | 23.68 | 1 | F1:2916 | 21L153\_Guenther\_MPI\_Tr.raw | 1.61E7 |  |  |  | 3 | 3 | 0 | 0 | 0 | 1304 | 1311 |  |
| L.YYGSGHLHQINL.D | Y | 51.25 | 1400.6837 | 12 | -1.2 | 701.3483 | 2 | 23.63 | 2 | F2:2900 | 21L153\_Guenther\_MPI\_AspN.raw |  | 3.62E6 |  |  | 2 | 0 | 2 | 0 | 0 | 985 | 996 |  |
| K.RYGYDLLN(+.98)NVVELQSHPAPYGTGLSAVPAQPIAPIIHR.F | Y | 51.14 | 4127.1592 | 38 | 2.6 | 1032.7998 | 4 | 31.52 | 1 | F1:4324 | 21L153\_Guenther\_MPI\_Tr.raw | 4.7E7 |  |  |  | 6 | 6 | 0 | 0 | 0 | 859 | 896 | Deamidation (NQ) |
| G.DLAQVRDSEGQVQRRFAY.D | Y | 51.06 | 2137.0664 | 18 | 2.5 | 713.3645 | 3 | 22.30 | 2 | F2:2665 | 21L153\_Guenther\_MPI\_AspN.raw |  | 2.72E7 |  |  | 3 | 0 | 3 | 0 | 0 | 504 | 521 |  |
| N.LTQTQLPDKR.W | Y | 50.90 | 1198.6670 | 10 | -0.6 | 600.3404 | 2 | 13.29 | 1 | F1:1076 | 21L153\_Guenther\_MPI\_Tr.raw | 5.74E6 |  |  |  | 4 | 4 | 0 | 0 | 0 | 970 | 979 |  |
| E.RLYPDQQR.E | Y | 50.87 | 1074.5570 | 8 | -0.5 | 538.2855 | 2 | 12.97 | 1 | F1:1008 | 21L153\_Guenther\_MPI\_Tr.raw | 1.88E6 |  |  |  | 3 | 3 | 0 | 0 | 0 | 484 | 491 |  |
| R.WN(+.98)SSGQLLEHLDC(+57.02)SGYPTR.F | Y | 50.79 | 2219.9905 | 19 | 2.3 | 741.0058 | 3 | 28.97 | 1 | F1:3870 | 21L153\_Guenther\_MPI\_Tr.raw | 1.8E7 |  |  |  | 1 | 1 | 0 | 0 | 0 | 715 | 733 | Deamidation (NQ); Carbamidomethylation |
| V.DGLGAQQKIR.Y | Y | 50.73 | 1084.5989 | 10 | 0.2 | 543.3068 | 2 | 13.25 | 2 | F2:1003 | 21L153\_Guenther\_MPI\_AspN.raw |  | 2.51E7 |  |  | 6 | 0 | 6 | 0 | 0 | 1236 | 1245 |  |
| Y.DAEHRLIEVRNQTSSGETLVR.M | Y | 50.53 | 2409.2361 | 21 | 1.6 | 804.0873 | 3 | 21.17 | 2 | F2:2455 | 21L153\_Guenther\_MPI\_AspN.raw |  | 2.56E8 |  |  | 7 | 0 | 7 | 0 | 0 | 1156 | 1176 |  |
| I.DALGEHTRFH.Y | Y | 50.10 | 1181.5577 | 10 | -0.1 | 394.8598 | 3 | 14.07 | 2 | F2:1163 | 21L153\_Guenther\_MPI\_AspN.raw |  | 1.57E6 |  |  | 2 | 0 | 2 | 0 | 0 | 747 | 756 |  |
| N.QTSSGETLVR.M | Y | 49.93 | 1076.5461 | 10 | 0.1 | 539.2804 | 2 | 13.98 | 1 | F1:1202 | 21L153\_Guenther\_MPI\_Tr.raw | 1.91E7 |  |  |  | 3 | 3 | 0 | 0 | 0 | 1167 | 1176 |  |
| N.TLPTAPERHLEY.D | Y | 49.82 | 1425.7252 | 12 | 2.6 | 713.8717 | 2 | 19.94 | 2 | F2:2228 | 21L153\_Guenther\_MPI\_AspN.raw |  | 1.71E7 |  |  | 4 | 0 | 4 | 0 | 0 | 1045 | 1056 |  |
| Y.DPRGNC(+57.02)IAEV.D | Y | 49.78 | 1129.5186 | 10 | 1.3 | 565.7673 | 2 | 17.28 | 2 | F2:1745 | 21L153\_Guenther\_MPI\_AspN.raw |  | 4.66E8 |  |  | 3 | 0 | 3 | 0 | 0 | 674 | 683 | Carbamidomethylation |
| R.AEHFQR.S | Y | 49.75 | 786.3773 | 6 | 0.0 | 394.1959 | 2 | 12.20 | 1 | F1:890 | 21L153\_Guenther\_MPI\_Tr.raw | 8.19E5 |  |  |  | 3 | 3 | 0 | 0 | 0 | 773 | 778 |  |
| Y.DRRGNVKAII.D | Y | 49.36 | 1140.6727 | 10 | -0.7 | 571.3433 | 2 | 14.96 | 2 | F2:1337 | 21L153\_Guenther\_MPI\_AspN.raw |  | 8.68E8 |  |  | 3 | 0 | 3 | 0 | 0 | 737 | 746 |  |
| N.DNRLHLEYDSSGRLWLLR.D | Y | 49.35 | 2242.1606 | 18 | 2.0 | 561.5486 | 4 | 30.73 | 2 | F2:4199 | 21L153\_Guenther\_MPI\_AspN.raw |  | 7.45E6 |  |  | 2 | 0 | 2 | 0 | 0 | 442 | 459 |  |
| R.YAYSYDETGNLIAETDPLGHSH.S | Y | 49.31 | 2452.0818 | 22 | 0.7 | 818.3685 | 3 | 30.29 | 1 | F1:4107 | 21L153\_Guenther\_MPI\_Tr.raw | 1.22E7 |  |  |  | 3 | 3 | 0 | 0 | 0 | 626 | 647 |  |
| G.DRNDNRLHLEY.D | Y | 49.03 | 1443.6854 | 11 | 2.2 | 722.8516 | 2 | 19.41 | 2 | F2:2133 | 21L153\_Guenther\_MPI\_AspN.raw |  | 2.37E7 |  |  | 5 | 0 | 5 | 0 | 0 | 439 | 449 |  |
| K.RYGYDLLNNVVELQSHPAPY.G | Y | 49.01 | 2347.1597 | 20 | 1.3 | 783.3948 | 3 | 32.75 | 1 | F1:4541 | 21L153\_Guenther\_MPI\_Tr.raw | 4.33E6 |  |  |  | 2 | 2 | 0 | 0 | 0 | 859 | 878 |  |
| R.LLQEQR.N | Y | 48.88 | 785.4395 | 6 | 0.4 | 393.7272 | 2 | 13.07 | 1 | F1:1029 | 21L153\_Guenther\_MPI\_Tr.raw | 5.37E6 |  |  |  | 3 | 3 | 0 | 0 | 0 | 1210 | 1215 |  |
| P.LGQVTQYRH.D | Y | 48.71 | 1100.5726 | 9 | -0.5 | 367.8646 | 3 | 13.14 | 2 | F2:981 | 21L153\_Guenther\_MPI\_AspN.raw |  | 8.59E6 |  |  | 3 | 0 | 3 | 0 | 0 | 686 | 694 |  |
| I.DAAGRSRKMRWNSSGQLLEHL.D | Y | 48.69 | 2411.2241 | 21 | 1.2 | 804.7496 | 3 | 24.01 | 2 | F2:2970 | 21L153\_Guenther\_MPI\_AspN.raw |  | 7.35E8 |  |  | 6 | 0 | 6 | 0 | 0 | 705 | 725 |  |
| R.IAQVER.L | Y | 48.50 | 714.4024 | 6 | 0.5 | 358.2086 | 2 | 12.56 | 1 | F1:941 | 21L153\_Guenther\_MPI\_Tr.raw | 2.04E6 |  |  |  | 3 | 3 | 0 | 0 | 0 | 479 | 484 |  |
| R.DTFEQAQL.E | Y | 48.38 | 950.4345 | 8 | 1.3 | 476.2251 | 2 | 24.29 | 2 | F2:3025 | 21L153\_Guenther\_MPI\_AspN.raw |  | 3.16E7 |  |  | 3 | 0 | 3 | 0 | 0 | 460 | 467 |  |
| Y.DVLGRRISKSEY.D | Y | 48.26 | 1421.7626 | 12 | 1.3 | 711.8895 | 2 | 15.41 | 2 | F2:1416 | 21L153\_Guenther\_MPI\_AspN.raw |  | 2.6E9 |  |  | 9 | 0 | 9 | 0 | 0 | 1180 | 1191 |  |
| R.DAVGRLLAK.I | Y | 48.03 | 941.5658 | 9 | -0.2 | 471.7901 | 2 | 18.36 | 2 | F2:1944 | 21L153\_Guenther\_MPI\_AspN.raw |  | 1.43E7 |  |  | 3 | 0 | 3 | 0 | 0 | 900 | 908 |  |
| Y.DC(+57.02)QGRLLSSQLADGRA.E | Y | 47.98 | 1745.8479 | 16 | 1.9 | 873.9329 | 2 | 20.24 | 2 | F2:2283 | 21L153\_Guenther\_MPI\_AspN.raw |  | 1.21E7 |  |  | 4 | 0 | 4 | 0 | 0 | 758 | 773 | Carbamidomethylation |
| I.IDALGEHTR.F | Y | 47.96 | 1010.5145 | 9 | -2.6 | 506.2632 | 2 | 21.89 | 1 | F1:2591 | 21L153\_Guenther\_MPI\_Tr.raw | 4.45E7 |  |  |  | 2 | 2 | 0 | 0 | 0 | 746 | 754 |  |
| L.DFTLPGLMPIEWQRFYNSR.D | Y | 47.55 | 2369.1626 | 19 | 3.3 | 1185.5925 | 2 | 34.25 | 2 | F2:4817 | 21L153\_Guenther\_MPI\_AspN.raw |  | 3.5E7 |  |  | 3 | 0 | 3 | 0 | 0 | 322 | 340 |  |
| G.DLAQVR.D | Y | 47.52 | 700.3868 | 6 | -0.7 | 351.2004 | 2 | 13.91 | 2 | F2:1133 | 21L153\_Guenther\_MPI\_AspN.raw |  | 7.98E6 |  |  | 3 | 0 | 3 | 0 | 0 | 504 | 509 |  |
| Q.N(+.98)LRFQGQYL.D | Y | 47.47 | 1138.5770 | 9 | 3.8 | 570.2980 | 2 | 28.51 | 2 | F2:3806 | 21L153\_Guenther\_MPI\_AspN.raw |  | 4.27E6 |  |  | 2 | 0 | 2 | 0 | 0 | 1291 | 1299 | Deamidation (NQ) |
| Y.DDSDNLVGLAERHATGQHRQLFHY.D | Y | 47.41 | 2778.3223 | 24 | 1.6 | 927.1161 | 3 | 26.52 | 2 | F2:3437 | 21L153\_Guenther\_MPI\_AspN.raw |  | 1.42E9 |  |  | 8 | 0 | 8 | 0 | 0 | 1057 | 1080 |  |
| N.DERQLLSATD.P | Y | 47.29 | 1146.5516 | 10 | 1.4 | 574.2839 | 2 | 19.20 | 2 | F2:2095 | 21L153\_Guenther\_MPI\_AspN.raw |  | 4.07E7 |  |  | 3 | 0 | 3 | 0 | 0 | 611 | 620 |  |
| L.DRETGLHFN(+.98)TFRFY.D | Y | 46.73 | 1802.8375 | 14 | 4.5 | 601.9558 | 3 | 31.53 | 2 | F2:4343 | 21L153\_Guenther\_MPI\_AspN.raw |  | 6.54E7 |  |  | 1 | 0 | 1 | 0 | 0 | 1300 | 1313 | Deamidation (NQ) |
| Y.DELGN(+.98)LTQTQLP.D | Y | 46.54 | 1328.6460 | 12 | 4.4 | 665.3332 | 2 | 28.73 | 2 | F2:3847 | 21L153\_Guenther\_MPI\_AspN.raw |  | 1.88E6 |  |  | 1 | 0 | 1 | 0 | 0 | 965 | 976 | Deamidation (NQ) |
| R.RNENGQ(+.98)LLIESS.D | Y | 46.38 | 1359.6630 | 12 | 1.1 | 680.8395 | 2 | 21.65 | 2 | F2:2540 | 21L153\_Guenther\_MPI\_AspN.raw |  | 3.64E6 |  |  | 1 | 0 | 1 | 0 | 0 | 404 | 415 | Deamidation (NQ) |
| P.DIGRFTTP.D | Y | 46.11 | 905.4606 | 8 | 0.8 | 453.7379 | 2 | 22.11 | 2 | F2:2629 | 21L153\_Guenther\_MPI\_AspN.raw |  | 1.19E9 |  |  | 3 | 0 | 3 | 0 | 0 | 1316 | 1323 |  |
| R.LLYIDEQGRR.I | Y | 46.09 | 1261.6779 | 10 | -0.9 | 631.8456 | 2 | 19.36 | 1 | F1:2160 | 21L153\_Guenther\_MPI\_Tr.raw | 1.12E8 |  |  |  | 3 | 3 | 0 | 0 | 0 | 372 | 381 |  |
| A.ERHATGQHRQLFHY.D | Y | 45.94 | 1778.8713 | 14 | -1.3 | 593.9636 | 3 | 14.23 | 2 | F2:1195 | 21L153\_Guenther\_MPI\_AspN.raw |  | 1.87E8 |  |  | 6 | 0 | 6 | 0 | 0 | 1067 | 1080 |  |
| V.DPLGQVTQYRHDAHGQVVEII.D | Y | 45.92 | 2374.2029 | 21 | -2.4 | 792.4063 | 3 | 28.11 | 2 | F2:3732 | 21L153\_Guenther\_MPI\_AspN.raw |  | 1.11E7 |  |  | 2 | 0 | 2 | 0 | 0 | 684 | 704 |  |
| R.RNEN(+.98)GQLLIESS.D | Y | 44.95 | 1359.6630 | 12 | 0.5 | 680.8391 | 2 | 21.71 | 2 | F2:2552 | 21L153\_Guenther\_MPI\_AspN.raw |  | 3.64E6 |  |  | 1 | 0 | 1 | 0 | 0 | 404 | 415 | Deamidation (NQ) |
| A.IIDALGEHTR.F | Y | 44.75 | 1123.5985 | 10 | -3.0 | 562.8049 | 2 | 22.17 | 1 | F1:2638 | 21L153\_Guenther\_MPI\_Tr.raw | 3.56E6 |  |  |  | 2 | 2 | 0 | 0 | 0 | 745 | 754 |  |
| Y.EISVQIEPHPEGGERLLYI.D | Y | 44.68 | 2178.1321 | 19 | 1.0 | 727.0520 | 3 | 31.45 | 2 | F2:4328 | 21L153\_Guenther\_MPI\_AspN.raw |  | 2.96E7 |  |  | 2 | 0 | 2 | 0 | 0 | 357 | 375 |  |
| I.DAHGRQVRFDY.D | Y | 44.59 | 1362.6428 | 11 | -0.1 | 682.3286 | 2 | 16.57 | 2 | F2:1622 | 21L153\_Guenther\_MPI\_AspN.raw |  | 1.68E7 |  |  | 5 | 0 | 5 | 0 | 0 | 810 | 820 |  |
| N.DLNGLPQ(+.98)QLC(+57.02)EP.D | Y | 44.58 | 1383.6340 | 12 | 4.7 | 692.8276 | 2 | 27.51 | 2 | F2:3618 | 21L153\_Guenther\_MPI\_AspN.raw |  | 5.28E6 |  |  | 1 | 0 | 1 | 0 | 0 | 1250 | 1261 | Deamidation (NQ); Carbamidomethylation |
| D.PAGESWHLR.Y | Y | 44.56 | 1051.5199 | 9 | -0.3 | 351.5138 | 3 | 17.34 | 1 | F1:1814 | 21L153\_Guenther\_MPI\_Tr.raw | 2.32E7 |  |  |  | 3 | 3 | 0 | 0 | 0 | 664 | 672 |  |
| W.PGRIAQVERLYP.D | Y | 44.52 | 1397.7778 | 12 | -0.4 | 466.9330 | 3 | 23.80 | 2 | F2:2931 | 21L153\_Guenther\_MPI\_AspN.raw |  | 2.26E7 |  |  | 2 | 0 | 2 | 0 | 0 | 476 | 487 |  |
| P.DKRWLNRLYYGSGHLH.Q | Y | 44.15 | 2014.0286 | 16 | 0.8 | 504.5148 | 4 | 23.24 | 2 | F2:2829 | 21L153\_Guenther\_MPI\_AspN.raw |  | 6.84E7 |  |  | 3 | 0 | 3 | 0 | 0 | 977 | 992 |  |
| Y.YIEEQNLR.F | Y | 44.10 | 1063.5298 | 8 | -0.8 | 532.7717 | 2 | 15.39 | 1 | F1:1457 | 21L153\_Guenther\_MPI\_Tr.raw | 4.53E7 |  |  |  | 4 | 4 | 0 | 0 | 0 | 1286 | 1293 |  |
| R.LLALTNEN(+.98)GESYRFAWDAGNR.L | Y | 44.10 | 2397.1348 | 21 | 1.3 | 800.0532 | 3 | 32.41 | 1 | F1:4483 | 21L153\_Guenther\_MPI\_Tr.raw | 1.17E6 |  |  |  | 1 | 1 | 0 | 0 | 0 | 826 | 846 | Deamidation (NQ) |
| T.DPSGYTTLYQYNRRG.Q | Y | 43.81 | 1789.8384 | 15 | -0.3 | 597.6199 | 3 | 21.68 | 2 | F2:2547 | 21L153\_Guenther\_MPI\_AspN.raw |  | 1.36E7 |  |  | 3 | 0 | 3 | 0 | 0 | 789 | 803 |  |
| A.DGRAEHFQRSSN(+.98)GQLQGYTD.P | Y | 43.67 | 2265.9998 | 20 | 1.3 | 756.3415 | 3 | 14.56 | 2 | F2:1262 | 21L153\_Guenther\_MPI\_AspN.raw |  | 2.46E6 |  |  | 2 | 0 | 2 | 0 | 0 | 770 | 789 | Deamidation (NQ) |
| R.IDAHGR.Q | Y | 43.61 | 667.3401 | 6 | 0.1 | 334.6774 | 2 | 11.62 | 1 | F1:817 | 21L153\_Guenther\_MPI\_Tr.raw | 2.2E5 |  |  |  | 3 | 3 | 0 | 0 | 0 | 809 | 814 |  |
| L.LANTLPTAPER.H | Y | 43.59 | 1181.6404 | 11 | 0.2 | 591.8276 | 2 | 16.90 | 1 | F1:1736 | 21L153\_Guenther\_MPI\_Tr.raw | 1.77E6 |  |  |  | 3 | 3 | 0 | 0 | 0 | 1042 | 1052 |  |
| G.DRN(+.98)DNRLHLEY.D | Y | 43.23 | 1444.6694 | 11 | 0.8 | 482.5641 | 3 | 20.79 | 2 | F2:2383 | 21L153\_Guenther\_MPI\_AspN.raw |  | 1.13E7 |  |  | 3 | 0 | 3 | 0 | 0 | 439 | 449 | Deamidation (NQ) |
| M.PIEWQRFYNSR.D | Y | 43.18 | 1494.7368 | 11 | 1.2 | 499.2535 | 3 | 26.80 | 2 | F2:3489 | 21L153\_Guenther\_MPI\_AspN.raw |  | 1.22E6 |  |  | 1 | 0 | 1 | 0 | 0 | 330 | 340 |  |
| R.LSEQQDLDGSAKR.Y | Y | 42.89 | 1445.7109 | 13 | -0.4 | 482.9107 | 3 | 12.82 | 1 | F1:980 | 21L153\_Guenther\_MPI\_Tr.raw | 1.86E7 |  |  |  | 3 | 3 | 0 | 0 | 0 | 847 | 859 |  |
| A.DGRAEHFQRSS.N | Y | 42.85 | 1288.5908 | 11 | -0.5 | 430.5374 | 3 | 11.94 | 2 | F2:767 | 21L153\_Guenther\_MPI\_AspN.raw |  | 4.95E5 |  |  | 3 | 0 | 3 | 0 | 0 | 770 | 780 |  |
| E.DLGHAPLARVD.G | Y | 42.67 | 1162.6094 | 11 | -0.2 | 388.5437 | 3 | 17.45 | 2 | F2:1779 | 21L153\_Guenther\_MPI\_AspN.raw |  | 5.52E6 |  |  | 2 | 0 | 2 | 0 | 0 | 1226 | 1236 |  |
| Y.GTGLSAVPAQPIAPIIHRFER.D | Y | 42.63 | 2229.2380 | 21 | 0.6 | 558.3171 | 4 | 29.35 | 2 | F2:3955 | 21L153\_Guenther\_MPI\_AspN.raw |  | 1.18E7 |  |  | 2 | 0 | 2 | 0 | 0 | 879 | 899 |  |
| Y.DLLNNVVELQ(+.98)SHPAPYGTGLSAVPAQPIAPIIHRF.E | Y | 42.34 | 3734.9783 | 35 | 3.7 | 1246.0046 | 3 | 33.80 | 2 | F2:4736 | 21L153\_Guenther\_MPI\_AspN.raw |  | 5.02E6 |  |  | 1 | 0 | 1 | 0 | 0 | 863 | 897 | Deamidation (NQ) |
| L.DMAKEFFSQMWRPTVASPAPGSQTKEL.D | Y | 42.33 | 3038.4629 | 27 | 3.3 | 1013.8316 | 3 | 33.50 | 2 | F2:4683 | 21L153\_Guenther\_MPI\_AspN.raw |  | 4.32E6 |  |  | 3 | 0 | 3 | 0 | 0 | 155 | 181 |  |
| R.FHYDCQGR.L | Y | 42.01 | 1024.4185 | 8 | -2.3 | 513.2153 | 2 | 13.89 | 1 | F1:1183 | 21L153\_Guenther\_MPI\_Tr.raw | 1.81E6 |  |  |  | 1 | 1 | 0 | 0 | 0 | 755 | 762 |  |
| P.DIGRFTTPD.P | Y | 41.94 | 1020.4876 | 9 | 1.1 | 511.2516 | 2 | 20.98 | 2 | F2:2417 | 21L153\_Guenther\_MPI\_AspN.raw |  | 3.96E7 |  |  | 3 | 0 | 3 | 0 | 0 | 1316 | 1324 |  |
| R.FYN(+.98)SRDER.R | Y | 41.89 | 1086.4730 | 8 | -0.4 | 544.2436 | 2 | 12.81 | 1 | F1:978 | 21L153\_Guenther\_MPI\_Tr.raw | 5.2E6 |  |  |  | 6 | 6 | 0 | 0 | 0 | 336 | 343 | Deamidation (NQ) |
| H.RYDELGNLTQ(+.98)TQLPDKR.W | Y | 41.80 | 2047.0333 | 17 | 4.3 | 683.3547 | 3 | 23.38 | 1 | F1:2861 | 21L153\_Guenther\_MPI\_Tr.raw | 4.69E0 |  |  |  | 1 | 1 | 0 | 0 | 0 | 963 | 979 | Deamidation (NQ) |
| A.GSLIHRY.D | Y | 41.80 | 844.4555 | 7 | 2.0 | 423.2359 | 2 | 18.54 | 2 | F2:1976 | 21L153\_Guenther\_MPI\_AspN.raw |  | 4.03E6 |  |  | 3 | 0 | 3 | 0 | 0 | 958 | 964 |  |
| Y.DAFGRMIEKRS.G | Y | 41.79 | 1308.6608 | 11 | 0.4 | 437.2277 | 3 | 17.26 | 2 | F2:1743 | 21L153\_Guenther\_MPI\_AspN.raw |  | 4.3E6 |  |  | 2 | 0 | 2 | 0 | 0 | 1135 | 1145 |  |
| T.DSLQRLSR.R | Y | 41.42 | 973.5305 | 8 | 1.2 | 325.5178 | 3 | 13.62 | 2 | F2:1074 | 21L153\_Guenther\_MPI\_AspN.raw |  | 1.4E8 |  |  | 6 | 0 | 6 | 0 | 0 | 578 | 585 |  |
| R.DRLHREVLRTQGQISTRSEY.D | Y | 41.38 | 2443.2681 | 20 | 2.7 | 1222.6447 | 2 | 15.28 | 2 | F2:1394 | 21L153\_Guenther\_MPI\_AspN.raw |  | 1.31E9 |  |  | 8 | 0 | 8 | 0 | 0 | 1007 | 1026 |  |
| Y.DAEHRLI.E | Y | 41.34 | 852.4453 | 7 | -1.0 | 427.2295 | 2 | 15.75 | 2 | F2:1476 | 21L153\_Guenther\_MPI\_AspN.raw |  | 1.51E7 |  |  | 3 | 0 | 3 | 0 | 0 | 1156 | 1162 |  |
| S.DNLVGLAERHATGQHR.Q | Y | 41.26 | 1772.9030 | 16 | -0.8 | 591.9744 | 3 | 18.56 | 2 | F2:1979 | 21L153\_Guenther\_MPI\_AspN.raw |  | 3.48E7 |  |  | 3 | 0 | 3 | 0 | 0 | 1060 | 1075 |  |
| R.FVWDGLR.L | Y | 41.22 | 891.4603 | 7 | 0.5 | 446.7376 | 2 | 28.60 | 1 | F1:3801 | 21L153\_Guenther\_MPI\_Tr.raw | 1.1E9 |  |  |  | 5 | 5 | 0 | 0 | 0 | 1203 | 1209 |  |
| N.TLPTAPER.H | Y | 41.14 | 883.4763 | 8 | -0.1 | 442.7454 | 2 | 14.00 | 1 | F1:1206 | 21L153\_Guenther\_MPI\_Tr.raw | 1.38E8 |  |  |  | 3 | 3 | 0 | 0 | 0 | 1045 | 1052 |  |
| R.FEYDRR.G | Y | 41.07 | 884.4140 | 6 | 1.0 | 295.8122 | 3 | 13.21 | 1 | F1:1058 | 21L153\_Guenther\_MPI\_Tr.raw | 3.24E8 |  |  |  | 6 | 6 | 0 | 0 | 0 | 734 | 739 |  |
| R.DSEGQVQRRFAY.D | Y | 40.46 | 1454.6902 | 12 | -0.1 | 728.3523 | 2 | 17.40 | 2 | F2:1770 | 21L153\_Guenther\_MPI\_AspN.raw |  | 3.22E9 |  |  | 6 | 0 | 6 | 0 | 0 | 510 | 521 |  |
| R.IIASQDGLQGQ(+.98)KETFAYDAAANLLDGPK.A | Y | 40.42 | 2934.4609 | 28 | 3.9 | 979.1647 | 3 | 33.24 | 1 | F1:4627 | 21L153\_Guenther\_MPI\_Tr.raw | 7.32E6 |  |  |  | 1 | 1 | 0 | 0 | 0 | 1086 | 1113 | Deamidation (NQ) |
| Y.DDSDNLVGLA.E | Y | 40.41 | 1017.4614 | 10 | 1.3 | 509.7386 | 2 | 26.77 | 2 | F2:3483 | 21L153\_Guenther\_MPI\_AspN.raw |  | 7.59E6 |  |  | 3 | 0 | 3 | 0 | 0 | 1057 | 1066 |  |
| W.AFEWNDER.Q | Y | 40.34 | 1065.4515 | 8 | -1.5 | 533.7322 | 2 | 23.12 | 1 | F1:2811 | 21L153\_Guenther\_MPI\_Tr.raw | 1.16E7 |  |  |  | 3 | 3 | 0 | 0 | 0 | 606 | 613 |  |
| L.DRETGLHF.N | Y | 39.66 | 973.4617 | 8 | 1.4 | 487.7388 | 2 | 20.98 | 2 | F2:2418 | 21L153\_Guenther\_MPI\_AspN.raw |  | 1.7E7 |  |  | 4 | 0 | 4 | 0 | 0 | 1300 | 1307 |  |
| R.YQVWGN(+.98)TVEEIREPYYIEEQNLR.F | Y | 39.45 | 2928.3928 | 23 | 2.5 | 977.1406 | 3 | 31.35 | 1 | F1:4293 | 21L153\_Guenther\_MPI\_Tr.raw | 1.43E7 |  |  |  | 1 | 1 | 0 | 0 | 0 | 1271 | 1293 | Deamidation (NQ) |
| Y.DAFGRM(+15.99)IEKR.S | Y | 39.40 | 1237.6237 | 10 | 0.0 | 413.5485 | 3 | 13.53 | 2 | F2:1055 | 21L153\_Guenther\_MPI\_AspN.raw |  | 1.88E7 |  |  | 3 | 0 | 3 | 0 | 0 | 1135 | 1144 | Oxidation (M) |
| H.TVAGSLIHR.Y | Y | 39.37 | 952.5454 | 9 | -1.6 | 477.2792 | 2 | 15.59 | 1 | F1:1495 | 21L153\_Guenther\_MPI\_Tr.raw | 2.44E6 |  |  |  | 4 | 4 | 0 | 0 | 0 | 955 | 963 |  |
| L.N(+.98)RLYYGSGHLHQINL.D | Y | 39.37 | 1784.8958 | 15 | 0.8 | 595.9730 | 3 | 24.84 | 2 | F2:3128 | 21L153\_Guenther\_MPI\_AspN.raw |  | 1.14E7 |  |  | 1 | 0 | 1 | 0 | 0 | 982 | 996 | Deamidation (NQ) |
| R.LYYGSGHLH.Q | Y | 39.36 | 1045.4980 | 9 | -0.4 | 349.5065 | 3 | 15.06 | 1 | F1:1394 | 21L153\_Guenther\_MPI\_Tr.raw | 2.95E6 |  |  |  | 3 | 3 | 0 | 0 | 0 | 984 | 992 |  |
| T.DDGRSQYTYD.P | Y | 39.23 | 1218.4789 | 10 | -2.1 | 610.2454 | 2 | 14.04 | 2 | F2:1158 | 21L153\_Guenther\_MPI\_AspN.raw |  | 3.78E6 |  |  | 3 | 0 | 3 | 0 | 0 | 911 | 920 |  |
| L.GETRFVW.D | Y | 38.76 | 893.4395 | 7 | -0.5 | 447.7268 | 2 | 28.17 | 2 | F2:3743 | 21L153\_Guenther\_MPI\_AspN.raw |  | 7.74E6 |  |  | 1 | 0 | 1 | 0 | 0 | 1199 | 1205 |  |
| W.DGLRLLQ.E | Y | 38.28 | 813.4708 | 7 | 1.1 | 407.7431 | 2 | 25.64 | 2 | F2:3275 | 21L153\_Guenther\_MPI\_AspN.raw |  | 9.84E8 |  |  | 3 | 0 | 3 | 0 | 0 | 1206 | 1212 |  |
| L.GEHTRFHY.D | Y | 38.25 | 1045.4729 | 8 | -1.0 | 523.7432 | 2 | 17.96 | 2 | F2:1871 | 21L153\_Guenther\_MPI\_AspN.raw |  | 1.87E7 |  |  | 2 | 0 | 2 | 0 | 0 | 750 | 757 |  |
| L.DRETGLHFNTFRFYDPDIGRFTTP.D | Y | 38.19 | 2901.3833 | 24 | 1.5 | 726.3542 | 4 | 32.34 | 2 | F2:4481 | 21L153\_Guenther\_MPI\_AspN.raw |  | 7.83E6 |  |  | 2 | 0 | 2 | 0 | 0 | 1300 | 1323 |  |
| Y.GYDLLNNVVELQSHPAPYGTGLSAVPAQPIAPIIHR.F | Y | 37.97 | 3807.0107 | 36 | 2.4 | 952.7623 | 4 | 33.47 | 1 | F1:4665 | 21L153\_Guenther\_MPI\_Tr.raw | 7.5E6 |  |  |  | 2 | 2 | 0 | 0 | 0 | 861 | 896 |  |
| R.FYNSR.D | Y | 37.91 | 685.3184 | 5 | 0.6 | 343.6667 | 2 | 12.92 | 1 | F1:998 | 21L153\_Guenther\_MPI\_Tr.raw | 9.33E5 |  |  |  | 3 | 3 | 0 | 0 | 0 | 336 | 340 |  |
| I.DALGEHTR.F | Y | 37.86 | 897.4304 | 8 | -4.0 | 449.7207 | 2 | 21.92 | 1 | F1:2596 | 21L153\_Guenther\_MPI\_Tr.raw | 1.41E7 |  |  |  | 2 | 2 | 0 | 0 | 0 | 747 | 754 |  |
| L.FHYDATGR.I | Y | 37.86 | 965.4355 | 8 | -2.9 | 483.7236 | 2 | 19.47 | 1 | F1:2182 | 21L153\_Guenther\_MPI\_Tr.raw | 6.85E6 |  |  |  | 2 | 2 | 0 | 0 | 0 | 1078 | 1085 |  |
| R.SSNGQ(+.98)LQGYTDPSGY.T | Y | 37.75 | 1573.6532 | 15 | -3.8 | 787.8309 | 2 | 23.42 | 1 | F1:2869 | 21L153\_Guenther\_MPI\_Tr.raw | 1.35E6 |  |  |  | 1 | 1 | 0 | 0 | 0 | 779 | 793 | Deamidation (NQ) |
| A.DGRAEHFQRSSN(+.98)GQLQG.Y | Y | 37.36 | 1886.8619 | 17 | 1.2 | 629.9620 | 3 | 13.04 | 2 | F2:959 | 21L153\_Guenther\_MPI\_AspN.raw |  | 3.42E6 |  |  | 2 | 0 | 2 | 0 | 0 | 770 | 786 | Deamidation (NQ) |
| R.M(+15.99)RYDVLGR.R | Y | 37.17 | 1024.5123 | 8 | 0.0 | 342.5114 | 3 | 16.36 | 1 | F1:1638 | 21L153\_Guenther\_MPI\_Tr.raw | 2.14E7 |  |  |  | 2 | 2 | 0 | 0 | 0 | 1177 | 1184 | Oxidation (M) |
| R.LQHSSLLAN.T | Y | 37.15 | 981.5244 | 9 | -0.9 | 491.7690 | 2 | 16.39 | 1 | F1:1644 | 21L153\_Guenther\_MPI\_Tr.raw | 1.31E8 |  |  |  | 3 | 3 | 0 | 0 | 0 | 1036 | 1044 |  |
| R.SSN(+.98)GQLQGYTD.P | Y | 37.07 | 1169.4836 | 11 | 0.4 | 585.7493 | 2 | 15.51 | 1 | F1:1481 | 21L153\_Guenther\_MPI\_Tr.raw | 8.69E6 |  |  |  | 3 | 3 | 0 | 0 | 0 | 779 | 789 | Deamidation (NQ) |
| R.DGLLGAGWSLPYEISVQIEPHPEGGER.L | Y | 36.94 | 2905.4246 | 27 | 3.5 | 969.4855 | 3 | 34.15 | 1 | F1:4783 | 21L153\_Guenther\_MPI\_Tr.raw | 2.1E6 |  |  |  | 1 | 1 | 0 | 0 | 0 | 345 | 371 |  |
| P.LNQLTAASFT.D | Y | 36.77 | 1064.5502 | 10 | 2.7 | 533.2838 | 2 | 27.07 | 2 | F2:3537 | 21L153\_Guenther\_MPI\_AspN.raw |  | 5.76E6 |  |  | 2 | 0 | 2 | 0 | 0 | 922 | 931 |  |
| Y.DAFGRM(+15.99)IEKRS.G | Y | 36.42 | 1324.6558 | 11 | 0.6 | 442.5595 | 3 | 13.54 | 2 | F2:1058 | 21L153\_Guenther\_MPI\_AspN.raw |  | 4.72E6 |  |  | 3 | 0 | 3 | 0 | 0 | 1135 | 1145 | Oxidation (M) |
| D.ALGEHTR.F | Y | 36.14 | 782.4035 | 7 | -1.7 | 392.2084 | 2 | 22.21 | 1 | F1:2647 | 21L153\_Guenther\_MPI\_Tr.raw | 8.67E5 |  |  |  | 1 | 1 | 0 | 0 | 0 | 748 | 754 |  |
| Y.PTRFEY.D | Y | 35.80 | 811.3864 | 6 | -0.6 | 406.7003 | 2 | 18.09 | 2 | F2:1893 | 21L153\_Guenther\_MPI\_AspN.raw |  | 2.32E6 |  |  | 2 | 0 | 2 | 0 | 0 | 731 | 736 |  |
| R.ETGLHFN.T | Y | 35.72 | 816.3766 | 7 | 1.6 | 409.1962 | 2 | 19.33 | 1 | F1:2154 | 21L153\_Guenther\_MPI\_Tr.raw | 1.34E7 |  |  |  | 3 | 3 | 0 | 0 | 0 | 1302 | 1308 |  |
| G.DSSRLRLSLLG.D | Y | 35.70 | 1215.6935 | 11 | 0.8 | 406.2388 | 3 | 27.73 | 2 | F2:3660 | 21L153\_Guenther\_MPI\_AspN.raw |  | 2.35E9 |  |  | 5 | 0 | 5 | 0 | 0 | 428 | 438 |  |
| R.RGVQRFAY.D | Y | 35.45 | 995.5300 | 8 | 0.3 | 498.7725 | 2 | 16.02 | 2 | F2:1524 | 21L153\_Guenther\_MPI\_AspN.raw |  | 5.22E6 |  |  | 3 | 0 | 3 | 0 | 0 | 1148 | 1155 |  |
| A.DGRAEHFQRSSNGQLQGYT.D | Y | 35.08 | 2149.9890 | 19 | 0.5 | 717.6707 | 3 | 14.55 | 2 | F2:1259 | 21L153\_Guenther\_MPI\_AspN.raw |  | 1.53E8 |  |  | 3 | 0 | 3 | 0 | 0 | 770 | 788 |  |
| D.AAANLLDGPK.A | Y | 35.02 | 968.5291 | 10 | -1.5 | 485.2711 | 2 | 19.34 | 1 | F1:2156 | 21L153\_Guenther\_MPI\_Tr.raw | 9.55E5 |  |  |  | 1 | 1 | 0 | 0 | 0 | 1104 | 1113 |  |
| R.RRWNSQLQIVEYT.D | Y | 34.62 | 1691.8743 | 13 | 1.7 | 564.9663 | 3 | 25.94 | 2 | F2:3330 | 21L153\_Guenther\_MPI\_AspN.raw |  | 2.72E7 |  |  | 1 | 0 | 1 | 0 | 0 | 586 | 598 |  |
| S.DNLVGLAERHATGQHRQLFHYDATGRIIASQ.D | Y | 34.55 | 3473.7664 | 31 | 2.7 | 869.4512 | 4 | 25.96 | 2 | F2:3333 | 21L153\_Guenther\_MPI\_AspN.raw |  | 1.66E7 |  |  | 2 | 0 | 2 | 0 | 0 | 1060 | 1090 |  |
| L.DRETGLHFNTFRF.Y | Y | 34.54 | 1638.7903 | 13 | 3.6 | 547.2726 | 3 | 29.64 | 2 | F2:4005 | 21L153\_Guenther\_MPI\_AspN.raw |  | 3.36E6 |  |  | 2 | 0 | 2 | 0 | 0 | 1300 | 1312 |  |
| R.LIEVR.N | Y | 34.36 | 628.3908 | 5 | 0.0 | 315.2027 | 2 | 15.27 | 1 | F1:1434 | 21L153\_Guenther\_MPI\_Tr.raw | 1.12E8 |  |  |  | 3 | 3 | 0 | 0 | 0 | 1161 | 1165 |  |
| Y.DSSGRLWLLR.D | Y | 34.35 | 1201.6567 | 10 | -2.9 | 401.5583 | 3 | 32.62 | 2 | F2:4529 | 21L153\_Guenther\_MPI\_AspN.raw |  | 1.22E9 |  |  | 3 | 0 | 3 | 0 | 0 | 450 | 459 |  |
| D.SSGRLWLLR.D | Y | 34.29 | 1086.6298 | 9 | 1.2 | 544.3228 | 2 | 28.86 | 2 | F2:3873 | 21L153\_Guenther\_MPI\_AspN.raw |  | 9.39E6 |  |  | 2 | 0 | 2 | 0 | 0 | 451 | 459 |  |
| W.RPTVASPAPGSQTK.E | Y | 34.23 | 1395.7469 | 14 | -0.8 | 466.2559 | 3 | 12.46 | 1 | F1:927 | 21L153\_Guenther\_MPI\_Tr.raw | 2.87E5 |  |  |  | 2 | 2 | 0 | 0 | 0 | 166 | 179 |  |
| T.DSLQRL.S | Y | 34.11 | 730.3973 | 6 | 0.7 | 366.2062 | 2 | 16.70 | 2 | F2:1643 | 21L153\_Guenther\_MPI\_AspN.raw |  | 4.46E7 |  |  | 3 | 0 | 3 | 0 | 0 | 578 | 583 |  |
| R.DAVGRLLAKITDD.G | Y | 34.10 | 1385.7513 | 13 | 3.2 | 693.8851 | 2 | 32.05 | 2 | F2:4431 | 21L153\_Guenther\_MPI\_AspN.raw |  | 1.16E6 |  |  | 2 | 0 | 2 | 0 | 0 | 900 | 912 |  |
| R.LSLLGDRN(+.98)DNR.L | Y | 33.98 | 1272.6422 | 11 | 1.0 | 637.3290 | 2 | 19.58 | 1 | F1:2200 | 21L153\_Guenther\_MPI\_Tr.raw | 1.44E7 |  |  |  | 7 | 7 | 0 | 0 | 0 | 434 | 444 | Deamidation (NQ) |
| L.NN(+.98)VVELQSHPAPYGTGLSAVPAQPIAPIIHRFER.D | Y | 33.85 | 3678.9270 | 34 | -0.3 | 920.7387 | 4 | 30.68 | 2 | F2:4190 | 21L153\_Guenther\_MPI\_AspN.raw |  | 3.51E7 |  |  | 1 | 0 | 1 | 0 | 0 | 866 | 899 | Deamidation (NQ) |
| H.ATGQHRQLFHY.D | Y | 33.74 | 1356.6687 | 11 | 0.4 | 453.2304 | 3 | 16.39 | 2 | F2:1589 | 21L153\_Guenther\_MPI\_AspN.raw |  | 3.73E6 |  |  | 2 | 0 | 2 | 0 | 0 | 1070 | 1080 |  |
| S.VQIEPHPEGGER.L | Y | 33.67 | 1346.6578 | 12 | -0.8 | 449.8929 | 3 | 13.39 | 1 | F1:1095 | 21L153\_Guenther\_MPI\_Tr.raw | 2.65E6 |  |  |  | 2 | 2 | 0 | 0 | 0 | 360 | 371 |  |
| R.IAQVERLYP.D | Y | 33.48 | 1087.6025 | 9 | 1.1 | 544.8091 | 2 | 24.30 | 2 | F2:3027 | 21L153\_Guenther\_MPI\_AspN.raw |  | 1.69E7 |  |  | 3 | 0 | 3 | 0 | 0 | 479 | 487 |  |
| Y.DLLN(+.98)NVVELQSHPAPYGTGLSAVPAQPIAPIIHRFER.D | Y | 33.44 | 4020.1221 | 37 | 2.3 | 805.0335 | 5 | 32.75 | 2 | F2:4553 | 21L153\_Guenther\_MPI\_AspN.raw |  | 6.91E7 |  |  | 2 | 0 | 2 | 0 | 0 | 863 | 899 | Deamidation (NQ) |
| Y.DAEHRLIEVRNQTSSGETLVRMRY.D | Y | 33.38 | 2859.4409 | 24 | 2.9 | 954.1570 | 3 | 24.49 | 2 | F2:3062 | 21L153\_Guenther\_MPI\_AspN.raw |  | 1.36E9 |  |  | 5 | 0 | 5 | 0 | 0 | 1156 | 1179 |  |
| V.AGSLIHRY.D | Y | 33.24 | 915.4926 | 8 | 0.0 | 458.7536 | 2 | 18.54 | 2 | F2:1977 | 21L153\_Guenther\_MPI\_AspN.raw |  | 4.19E6 |  |  | 3 | 0 | 3 | 0 | 0 | 957 | 964 |  |
| A.EHFQRSSNGQLQGYT.D | Y | 32.99 | 1750.8022 | 15 | 0.6 | 584.6084 | 3 | 14.63 | 2 | F2:1274 | 21L153\_Guenther\_MPI\_AspN.raw |  | 1.07E8 |  |  | 2 | 0 | 2 | 0 | 0 | 774 | 788 |  |
| H.DAHGQVVEIIDAAGRSRKMRWNSSGQLLEHL.D | Y | 32.99 | 3472.7744 | 31 | -0.9 | 695.5615 | 5 | 30.86 | 2 | F2:4223 | 21L153\_Guenther\_MPI\_AspN.raw |  | 2.05E7 |  |  | 1 | 0 | 1 | 0 | 0 | 695 | 725 |  |
| R.RDGLLGAGW.S | Y | 32.82 | 943.4875 | 9 | 0.0 | 472.7511 | 2 | 30.23 | 1 | F1:4097 | 21L153\_Guenther\_MPI\_Tr.raw | 4.87E6 |  |  |  | 1 | 1 | 0 | 0 | 0 | 344 | 352 |  |
| R.YYHNDLNGLPQQ.L | Y | 32.67 | 1460.6685 | 12 | 3.7 | 731.3442 | 2 | 19.08 | 1 | F1:2107 | 21L153\_Guenther\_MPI\_Tr.raw | 6.57E5 |  |  |  | 4 | 4 | 0 | 0 | 0 | 1246 | 1257 |  |
| T.QVTDSLQR.L | Y | 32.50 | 945.4879 | 8 | -2.8 | 473.7499 | 2 | 13.48 | 1 | F1:1113 | 21L153\_Guenther\_MPI\_Tr.raw | 9.69E6 |  |  |  | 1 | 1 | 0 | 0 | 0 | 575 | 582 |  |
| I.DEQGRRI.D | Y | 32.46 | 872.4464 | 7 | -0.4 | 437.2303 | 2 | 12.31 | 2 | F2:821 | 21L153\_Guenther\_MPI\_AspN.raw |  | 1.36E6 |  |  | 3 | 0 | 3 | 0 | 0 | 376 | 382 |  |
| Y.DAEHRLIEVRN(+.98)QTSSGETLVRM(+15.99)RY.D | Y | 32.32 | 2876.4199 | 24 | -0.1 | 576.2912 | 5 | 21.80 | 2 | F2:2569 | 21L153\_Guenther\_MPI\_AspN.raw |  | 2.78E7 |  |  | 5 | 0 | 5 | 0 | 0 | 1156 | 1179 | Deamidation (NQ); Oxidation (M) |
| L.SVRRN(+.98)ENGQLLIESS.D | Y | 32.30 | 1701.8645 | 15 | 0.1 | 851.9396 | 2 | 18.15 | 2 | F2:1905 | 21L153\_Guenther\_MPI\_AspN.raw |  | 6.78E6 |  |  | 1 | 0 | 1 | 0 | 0 | 401 | 415 | Deamidation (NQ) |
| L.AERHATGQHRQLFHY.D | Y | 32.27 | 1849.9084 | 15 | -0.1 | 463.4843 | 4 | 14.14 | 2 | F2:1179 | 21L153\_Guenther\_MPI\_AspN.raw |  | 3.74E6 |  |  | 1 | 0 | 1 | 0 | 0 | 1066 | 1080 |  |
| I.EVRNQTSSGETLVRMRY.D | Y | 32.27 | 2025.0061 | 17 | 0.7 | 676.0098 | 3 | 20.20 | 2 | F2:2277 | 21L153\_Guenther\_MPI\_AspN.raw |  | 2.44E7 |  |  | 2 | 0 | 2 | 0 | 0 | 1163 | 1179 |  |
| L.LYIDEQGRR.I | Y | 32.17 | 1148.5938 | 9 | 1.6 | 575.3051 | 2 | 19.45 | 1 | F1:2179 | 21L153\_Guenther\_MPI\_Tr.raw | 1.88E6 |  |  |  | 3 | 3 | 0 | 0 | 0 | 373 | 381 |  |
| R.RGQIR.Q | Y | 31.91 | 628.3769 | 5 | 0.5 | 315.1959 | 2 | 11.58 | 1 | F1:811 | 21L153\_Guenther\_MPI\_Tr.raw | 3.19E5 |  |  |  | 3 | 3 | 0 | 0 | 0 | 802 | 806 |  |
| S.SLLANTLPTAPERHLEY.D | Y | 31.83 | 1924.0054 | 17 | -0.8 | 642.3419 | 3 | 27.43 | 2 | F2:3603 | 21L153\_Guenther\_MPI\_AspN.raw |  | 7.45E6 |  |  | 2 | 0 | 2 | 0 | 0 | 1040 | 1056 |  |
| R.SQYTYDPLNQLTAASFTDNLGNQQALSFSYDALGQLLEEHTVAGSLIHR.Y | Y | 31.65 | 5396.6226 | 49 | 1.7 | 1350.1652 | 4 | 34.77 | 1 | F1:4877 | 21L153\_Guenther\_MPI\_Tr.raw | 1.13E6 |  |  |  | 1 | 1 | 0 | 0 | 0 | 915 | 963 |  |
| R.DRLHREVLRTQGQISTRS.E | Y | 31.62 | 2151.1621 | 18 | -0.4 | 538.7976 | 4 | 13.73 | 2 | F2:1099 | 21L153\_Guenther\_MPI\_AspN.raw |  | 3.45E7 |  |  | 2 | 0 | 2 | 0 | 0 | 1007 | 1024 |  |
| W.SLPQTLTDPAGESWHLR.Y | Y | 31.61 | 1906.9537 | 17 | 0.6 | 636.6589 | 3 | 28.25 | 1 | F1:3735 | 21L153\_Guenther\_MPI\_Tr.raw | 3.22E7 |  |  |  | 2 | 2 | 0 | 0 | 0 | 656 | 672 |  |
| Y.DAEHRLIEVRNQTSSGETLVRM(+15.99)RY.D | Y | 31.59 | 2875.4358 | 24 | 3.1 | 576.0962 | 5 | 22.63 | 2 | F2:2723 | 21L153\_Guenther\_MPI\_AspN.raw |  | 7.25E8 |  |  | 3 | 0 | 3 | 0 | 0 | 1156 | 1179 | Oxidation (M) |
| I.DAAGRSRKM(+15.99)RWNSSGQLLEHL.D | Y | 31.44 | 2427.2190 | 21 | 2.1 | 810.0820 | 3 | 22.55 | 2 | F2:2709 | 21L153\_Guenther\_MPI\_AspN.raw |  | 1.5E8 |  |  | 2 | 0 | 2 | 0 | 0 | 705 | 725 | Oxidation (M) |
| M.RWNSSGQLLEHL.D | Y | 31.41 | 1438.7317 | 12 | 1.5 | 480.5852 | 3 | 27.46 | 2 | F2:3609 | 21L153\_Guenther\_MPI\_AspN.raw |  | 1.91E6 |  |  | 1 | 0 | 1 | 0 | 0 | 714 | 725 |  |
| E.GQVQRRFAY.D | Y | 31.37 | 1123.5886 | 9 | 0.4 | 562.8018 | 2 | 17.57 | 2 | F2:1803 | 21L153\_Guenther\_MPI\_AspN.raw |  | 9.89E6 |  |  | 1 | 0 | 1 | 0 | 0 | 513 | 521 |  |
| I.DAAGRSRKMRWN(+.98)SSGQLLEHL.D | Y | 31.18 | 2412.2080 | 21 | 2.9 | 805.0789 | 3 | 25.98 | 2 | F2:3337 | 21L153\_Guenther\_MPI\_AspN.raw |  | 1.95E7 |  |  | 1 | 0 | 1 | 0 | 0 | 705 | 725 | Deamidation (NQ) |
| A.DQQWRVTRH.W | Y | 31.13 | 1224.6112 | 9 | 0.0 | 409.2110 | 3 | 13.37 | 2 | F2:1026 | 21L153\_Guenther\_MPI\_AspN.raw |  | 6.24E6 |  |  | 3 | 0 | 3 | 0 | 0 | 547 | 555 |  |
| L.GQVTQYRH.D | Y | 31.03 | 987.4886 | 8 | 2.6 | 494.7529 | 2 | 18.76 | 2 | F2:2016 | 21L153\_Guenther\_MPI\_AspN.raw |  | 3.08E6 |  |  | 1 | 0 | 1 | 0 | 0 | 687 | 694 |  |
| Y.DLLN(+.98)NVVELQSHPAPYGTGLSAVPAQPIAPIIHRF.E | Y | 30.84 | 3734.9783 | 35 | 1.9 | 934.7536 | 4 | 33.42 | 2 | F2:4667 | 21L153\_Guenther\_MPI\_AspN.raw |  | 6.08E6 |  |  | 1 | 0 | 1 | 0 | 0 | 863 | 897 | Deamidation (NQ) |
| I.EPHPEGGERLLYI.D | Y | 30.83 | 1508.7623 | 13 | 2.5 | 755.3903 | 2 | 27.56 | 2 | F2:3627 | 21L153\_Guenther\_MPI\_AspN.raw |  | 5.44E6 |  |  | 3 | 0 | 3 | 0 | 0 | 363 | 375 |  |
| Y.DAEHRLIEVRN(+.98)QTSSGETLVRMRY.D | Y | 30.64 | 2860.4250 | 24 | 1.3 | 573.0930 | 5 | 24.43 | 2 | F2:3051 | 21L153\_Guenther\_MPI\_AspN.raw |  | 5.57E8 |  |  | 3 | 0 | 3 | 0 | 0 | 1156 | 1179 | Deamidation (NQ) |
| R.ETGLHFNTF.R | Y | 30.53 | 1064.4927 | 9 | 0.8 | 533.2540 | 2 | 28.64 | 1 | F1:3808 | 21L153\_Guenther\_MPI\_Tr.raw | 5.74E6 |  |  |  | 3 | 3 | 0 | 0 | 0 | 1302 | 1310 |  |
| Y.DLLNNVVELQSHPAPYGTGLSAVPAQPIAPIIHRFER.D | Y | 30.37 | 4019.1379 | 37 | 1.2 | 1340.7216 | 3 | 33.72 | 2 | F2:4722 | 21L153\_Guenther\_MPI\_AspN.raw |  | 3.62E8 |  |  | 2 | 0 | 2 | 0 | 0 | 863 | 899 |  |
| Q.LLEEHTVAGSLIHR.Y | Y | 30.14 | 1573.8577 | 14 | -0.4 | 525.6263 | 3 | 22.04 | 1 | F1:2615 | 21L153\_Guenther\_MPI\_Tr.raw | 2.86E6 |  |  |  | 1 | 1 | 0 | 0 | 0 | 950 | 963 |  |
| R.SGRRGVQRFAY.D | Y | 29.92 | 1295.6847 | 11 | 1.7 | 432.9029 | 3 | 14.47 | 2 | F2:1244 | 21L153\_Guenther\_MPI\_AspN.raw |  | 3.23E7 |  |  | 3 | 0 | 3 | 0 | 0 | 1145 | 1155 |  |
| D.LGHAPLARV.D | Y | 29.88 | 932.5555 | 9 | 0.1 | 467.2851 | 2 | 19.49 | 2 | F2:2148 | 21L153\_Guenther\_MPI\_AspN.raw |  | 6.6E6 |  |  | 1 | 0 | 1 | 0 | 0 | 1227 | 1235 |  |
| P.AGESWHLRY.D | Y | 29.85 | 1117.5305 | 9 | 0.4 | 373.5176 | 3 | 23.57 | 2 | F2:2889 | 21L153\_Guenther\_MPI\_AspN.raw |  | 1.07E7 |  |  | 3 | 0 | 3 | 0 | 0 | 665 | 673 |  |
| L.ANTLPTAPER.H | Y | 29.69 | 1068.5564 | 10 | 0.2 | 535.2856 | 2 | 14.58 | 1 | F1:1305 | 21L153\_Guenther\_MPI\_Tr.raw | 2.88E6 |  |  |  | 1 | 1 | 0 | 0 | 0 | 1043 | 1052 |  |
| R.DRLHREVLR.T | Y | 29.67 | 1192.6788 | 9 | 1.3 | 299.1774 | 4 | 12.72 | 2 | F2:899 | 21L153\_Guenther\_MPI\_AspN.raw |  | 8.03E6 |  |  | 3 | 0 | 3 | 0 | 0 | 1007 | 1015 |  |
| N.LLGETRFVW.D | Y | 29.65 | 1119.6077 | 9 | -2.4 | 560.8098 | 2 | 33.43 | 2 | F2:4670 | 21L153\_Guenther\_MPI\_AspN.raw |  | 3.42E5 |  |  | 1 | 0 | 1 | 0 | 0 | 1197 | 1205 |  |
| L.SVRRNEN(+.98)GQLLIESS.D | Y | 29.63 | 1701.8645 | 15 | -0.4 | 851.9391 | 2 | 18.17 | 2 | F2:1907 | 21L153\_Guenther\_MPI\_AspN.raw |  | 6.78E6 |  |  | 1 | 0 | 1 | 0 | 0 | 401 | 415 | Deamidation (NQ) |
| A.DGRAEHFQRSSNGQ(+.98)LQGYT.D | Y | 29.52 | 2150.9729 | 19 | 3.2 | 538.7522 | 4 | 14.84 | 2 | F2:1314 | 21L153\_Guenther\_MPI\_AspN.raw |  | 2.98E8 |  |  | 1 | 0 | 1 | 0 | 0 | 770 | 788 | Deamidation (NQ) |
| Y.DAFGRM(+15.99)IEKRSGRRGVQRFAY.D | Y | 29.43 | 2515.2979 | 21 | 0.6 | 839.4404 | 3 | 16.13 | 2 | F2:1543 | 21L153\_Guenther\_MPI\_AspN.raw |  | 3.91E6 |  |  | 3 | 0 | 3 | 0 | 0 | 1135 | 1155 | Oxidation (M) |
| L.N(+.98)NVVELQSHPAPYGTGLSAVPAQPIAPIIHRFER.D | Y | 29.40 | 3678.9270 | 34 | 0.2 | 736.7928 | 5 | 30.74 | 2 | F2:4200 | 21L153\_Guenther\_MPI\_AspN.raw |  | 7.09E7 |  |  | 2 | 0 | 2 | 0 | 0 | 866 | 899 | Deamidation (NQ) |
| Y.DVLGRRISK.S | Y | 29.39 | 1042.6246 | 9 | 0.5 | 348.5490 | 3 | 13.50 | 2 | F2:1050 | 21L153\_Guenther\_MPI\_AspN.raw |  | 2.17E6 |  |  | 1 | 0 | 1 | 0 | 0 | 1180 | 1188 |  |
| L.PTAPER.H | Y | 29.29 | 669.3445 | 6 | 0.2 | 335.6796 | 2 | 24.76 | 1 | F1:3097 | 21L153\_Guenther\_MPI\_Tr.raw | 4.58E6 |  |  |  | 1 | 1 | 0 | 0 | 0 | 1047 | 1052 |  |
| G.ESYRFAW.D | Y | 29.28 | 957.4344 | 7 | 2.2 | 479.7256 | 2 | 29.90 | 2 | F2:4050 | 21L153\_Guenther\_MPI\_AspN.raw |  | 5.18E7 |  |  | 3 | 0 | 3 | 0 | 0 | 835 | 841 |  |
| V.PAQPIAPIIHRFER.D | Y | 29.18 | 1643.9259 | 14 | -2.5 | 822.9682 | 2 | 33.74 | 2 | F2:4725 | 21L153\_Guenther\_MPI\_AspN.raw |  | 1.09E6 |  |  | 1 | 0 | 1 | 0 | 0 | 886 | 899 |  |
| Y.STLWPGR.I | Y | 29.07 | 815.4290 | 7 | -1.5 | 408.7211 | 2 | 22.72 | 1 | F1:2736 | 21L153\_Guenther\_MPI\_Tr.raw | 5.81E6 |  |  |  | 1 | 1 | 0 | 0 | 0 | 472 | 478 |  |
| R.DRLHREVLRTQ.G | Y | 28.63 | 1421.7852 | 11 | 0.0 | 474.9356 | 3 | 12.87 | 2 | F2:924 | 21L153\_Guenther\_MPI\_AspN.raw |  | 6.75E6 |  |  | 3 | 0 | 3 | 0 | 0 | 1007 | 1017 |  |
| A.DQQWRVTRHWTDEG.D | Y | 28.62 | 1812.8292 | 14 | -0.2 | 605.2836 | 3 | 19.33 | 2 | F2:2120 | 21L153\_Guenther\_MPI\_AspN.raw |  | 6.93E6 |  |  | 2 | 0 | 2 | 0 | 0 | 547 | 560 |  |
| Y.DLLNN(+.98)VVELQSHPAPYGTGLSAVPAQPIAPIIHRFER.D | Y | 28.33 | 4020.1221 | 37 | 2.3 | 805.0335 | 5 | 32.73 | 2 | F2:4549 | 21L153\_Guenther\_MPI\_AspN.raw |  | 6.91E7 |  |  | 1 | 0 | 1 | 0 | 0 | 863 | 899 | Deamidation (NQ) |
| R.WNSSGQ(+.98)LLEHLD.C | Y | 27.25 | 1398.6415 | 12 | 3.0 | 700.3301 | 2 | 29.76 | 1 | F1:4015 | 21L153\_Guenther\_MPI\_Tr.raw | 0 |  |  |  | 1 | 1 | 0 | 0 | 0 | 715 | 726 | Deamidation (NQ) |
| Y.QYAPNPIGWI.D | Y | 27.09 | 1157.5869 | 10 | 0.3 | 579.8009 | 2 | 34.22 | 2 | F2:4813 | 21L153\_Guenther\_MPI\_AspN.raw |  | 1.43E6 |  |  | 2 | 0 | 2 | 0 | 0 | 1336 | 1345 |  |
| R.RGVQR.F | Y | 26.69 | 614.3612 | 5 | 0.2 | 308.1879 | 2 | 11.49 | 1 | F1:801 | 21L153\_Guenther\_MPI\_Tr.raw | 1.45E5 |  |  |  | 3 | 3 | 0 | 0 | 0 | 1148 | 1152 |  |
| D.NLVGLAERHATGQHRQLFHY.D | Y | 26.69 | 2346.2092 | 20 | 0.7 | 587.5600 | 4 | 23.72 | 2 | F2:2916 | 21L153\_Guenther\_MPI\_AspN.raw |  | 4.73E6 |  |  | 1 | 0 | 1 | 0 | 0 | 1061 | 1080 |  |
| D.SEGQVQRRFAY.D | Y | 26.67 | 1339.6632 | 11 | -0.2 | 670.8387 | 2 | 17.66 | 2 | F2:1819 | 21L153\_Guenther\_MPI\_AspN.raw |  | 5.52E6 |  |  | 1 | 0 | 1 | 0 | 0 | 511 | 521 |  |
| G.N(+.98)LLGETRFVW.D | Y | 26.57 | 1234.6346 | 10 | -3.7 | 618.3223 | 2 | 34.09 | 2 | F2:4790 | 21L153\_Guenther\_MPI\_AspN.raw |  | 8.43E5 |  |  | 1 | 0 | 1 | 0 | 0 | 1196 | 1205 | Deamidation (NQ) |
| H.QLATGLR.C | Y | 26.44 | 757.4446 | 7 | 0.0 | 379.7296 | 2 | 15.02 | 1 | F1:1385 | 21L153\_Guenther\_MPI\_Tr.raw | 6.04E6 |  |  |  | 2 | 2 | 0 | 0 | 0 | 531 | 537 |  |
| R.YYHNDLNGLPQQL.C | Y | 26.26 | 1573.7524 | 13 | 1.1 | 787.8843 | 2 | 26.12 | 1 | F1:3341 | 21L153\_Guenther\_MPI\_Tr.raw | 7.09E6 |  |  |  | 1 | 1 | 0 | 0 | 0 | 1246 | 1258 |  |
| R.SEYDR.C | Y | 26.19 | 668.2766 | 5 | 0.0 | 335.1455 | 2 | 12.04 | 1 | F1:867 | 21L153\_Guenther\_MPI\_Tr.raw | 1.94E5 |  |  |  | 1 | 1 | 0 | 0 | 0 | 1024 | 1028 |  |
| Q.IEPHPEGGER.L | Y | 25.94 | 1119.5309 | 10 | 0.2 | 374.1843 | 3 | 12.13 | 1 | F1:879 | 21L153\_Guenther\_MPI\_Tr.raw | 2.19E5 |  |  |  | 2 | 2 | 0 | 0 | 0 | 362 | 371 |  |
| Y.DRRGNVKAIIDALGEHTRFHY.D | Y | 25.88 | 2467.2832 | 21 | -3.2 | 494.4623 | 5 | 28.92 | 2 | F2:3883 | 21L153\_Guenther\_MPI\_AspN.raw |  | 4.97E7 |  |  | 2 | 0 | 2 | 0 | 0 | 737 | 757 |  |
| R.LWLLR.D | Y | 25.71 | 699.4432 | 5 | 0.2 | 350.7289 | 2 | 31.42 | 1 | F1:4305 | 21L153\_Guenther\_MPI\_Tr.raw | 4.84E8 |  |  |  | 3 | 3 | 0 | 0 | 0 | 455 | 459 |  |
| S.EGQVQRRFAY.D | Y | 25.51 | 1252.6312 | 10 | 0.1 | 627.3229 | 2 | 17.62 | 2 | F2:1811 | 21L153\_Guenther\_MPI\_AspN.raw |  | 1.24E7 |  |  | 1 | 0 | 1 | 0 | 0 | 512 | 521 |  |
| R.DRLHREVLRTQG.Q | Y | 25.48 | 1478.8065 | 12 | -0.3 | 493.9426 | 3 | 12.93 | 2 | F2:938 | 21L153\_Guenther\_MPI\_AspN.raw |  | 3.83E6 |  |  | 2 | 0 | 2 | 0 | 0 | 1007 | 1018 |  |
| Y.DRC(+57.02)GRLRARLQHSSLLANTLPTAPERHLEY.D | Y | 25.44 | 3529.8435 | 30 | 1.5 | 883.4695 | 4 | 24.20 | 2 | F2:3006 | 21L153\_Guenther\_MPI\_AspN.raw |  | 1.48E8 |  |  | 2 | 0 | 2 | 0 | 0 | 1027 | 1056 | Carbamidomethylation |
| S.DNLVGLAER.H | Y | 25.33 | 985.5192 | 9 | -3.4 | 493.7652 | 2 | 22.98 | 1 | F1:2783 | 21L153\_Guenther\_MPI\_Tr.raw | 1.06E6 |  |  |  | 1 | 1 | 0 | 0 | 0 | 1060 | 1068 |  |
| E.HQLATGLR.C | Y | 25.14 | 894.5035 | 8 | 0.1 | 448.2591 | 2 | 18.17 | 1 | F1:1961 | 21L153\_Guenther\_MPI\_Tr.raw | 1.2E6 |  |  |  | 1 | 1 | 0 | 0 | 0 | 530 | 537 |  |
| W.DGLRLLQEQRNQQTSLYVY.E | Y | 25.11 | 2323.1919 | 19 | 1.5 | 775.4057 | 3 | 29.36 | 2 | F2:3957 | 21L153\_Guenther\_MPI\_AspN.raw |  | 1.03E7 |  |  | 1 | 0 | 1 | 0 | 0 | 1206 | 1224 |  |
| N.TFRFY.D | Y | 24.50 | 732.3595 | 5 | 1.2 | 367.1875 | 2 | 27.20 | 2 | F2:3561 | 21L153\_Guenther\_MPI\_AspN.raw |  | 4.77E6 |  |  | 2 | 0 | 2 | 0 | 0 | 1309 | 1313 |  |
| Y.DDSDNLVGLAER.H | Y | 24.50 | 1302.6051 | 12 | 0.1 | 652.3099 | 2 | 25.17 | 1 | F1:3167 | 21L153\_Guenther\_MPI\_Tr.raw | 4.33E6 |  |  |  | 1 | 1 | 0 | 0 | 0 | 1057 | 1068 |  |
| L.DGPKAGAGLVVHNKL.L | Y | 24.44 | 1474.8256 | 15 | -1.0 | 492.6153 | 3 | 16.82 | 2 | F2:1663 | 21L153\_Guenther\_MPI\_AspN.raw |  | 2.32E6 |  |  | 1 | 0 | 1 | 0 | 0 | 1110 | 1124 |  |
| Y.DAFGRMIEKRSGR.R | Y | 24.24 | 1521.7833 | 13 | 1.3 | 508.2690 | 3 | 15.46 | 2 | F2:1425 | 21L153\_Guenther\_MPI\_AspN.raw |  | 2.17E6 |  |  | 1 | 0 | 1 | 0 | 0 | 1135 | 1147 |  |
| P.DKRWLNRLYYGSGHLHQINLDGQVVS.D | Y | 24.17 | 3067.5740 | 26 | 3.7 | 1023.5358 | 3 | 27.39 | 2 | F2:3597 | 21L153\_Guenther\_MPI\_AspN.raw |  | 3.16E7 |  |  | 2 | 0 | 2 | 0 | 0 | 977 | 1002 |  |
| A.EVDPLGQVTQYRH.D | Y | 24.08 | 1540.7634 | 13 | 0.4 | 514.5953 | 3 | 23.79 | 2 | F2:2928 | 21L153\_Guenther\_MPI\_AspN.raw |  | 1.49E8 |  |  | 1 | 0 | 1 | 0 | 0 | 682 | 694 |  |
| R.YRYDAF.G | Y | 24.07 | 833.3708 | 6 | -1.5 | 417.6920 | 2 | 23.19 | 1 | F1:2824 | 21L153\_Guenther\_MPI\_Tr.raw | 4.12E6 |  |  |  | 1 | 1 | 0 | 0 | 0 | 1132 | 1137 |  |
| R.DRLHREVLRTQGQISTR.S | Y | 23.96 | 2064.1301 | 17 | 0.3 | 517.0400 | 4 | 13.68 | 2 | F2:1088 | 21L153\_Guenther\_MPI\_AspN.raw |  | 1.85E7 |  |  | 1 | 0 | 1 | 0 | 0 | 1007 | 1023 |  |
| T.DSLQRLSRRRWNSQLQIVEYT.D | Y | 23.73 | 2647.3943 | 21 | 1.5 | 1324.7064 | 2 | 26.21 | 2 | F2:3380 | 21L153\_Guenther\_MPI\_AspN.raw |  | 7.33E6 |  |  | 1 | 0 | 1 | 0 | 0 | 578 | 598 |  |
| Y.DVLGRRISKSE.Y | Y | 23.55 | 1258.6993 | 11 | 0.3 | 420.5739 | 3 | 13.62 | 2 | F2:1075 | 21L153\_Guenther\_MPI\_AspN.raw |  | 1.19E6 |  |  | 1 | 0 | 1 | 0 | 0 | 1180 | 1190 |  |
| G.EHTRFHY.D | Y | 23.53 | 988.4515 | 7 | 0.1 | 330.4911 | 3 | 13.56 | 2 | F2:1061 | 21L153\_Guenther\_MPI\_AspN.raw |  | 1.54E7 |  |  | 1 | 0 | 1 | 0 | 0 | 751 | 757 |  |
| R.RGNVKAIIDALGEHTR.F | Y | 23.37 | 1748.9645 | 16 | -2.6 | 438.2473 | 4 | 25.83 | 1 | F1:3288 | 21L153\_Guenther\_MPI\_Tr.raw | 1.22E6 |  |  |  | 1 | 1 | 0 | 0 | 0 | 739 | 754 |  |
| total 450 peptides |
| --- |

Peptide List

  
  

---

Prepared with PEAKS ™ (bioinfor.com)
