# Supplementary material for: Structure of a bacterial Rhs effector exported by the type VI secretion system
Source: PLoS Pathog. 2022 Jan 5;18(1):e1010182. doi: 10.1371/journal.ppat.1010182 (PMC8765631; doi:10.1371/journal.ppat.1010182)
Supplement: Attachment — Submitted filename: Author_Response.docx [file ppat.1010182.s014.docx]

We thank the reviewers for their positive and constructive feedback, which aided us to further improve the manuscript. Major modifications of the manuscript are highlighted in yellow. Below we include our detailed response to each point raised.

**Reviewer #1 (remarks to the authors):**

**Major**

None

**Minor**

1) Figure 2C, y-axis: I just want to clarify that this is the Log10 of the Competitive Index as calculated in the methods, i.e. Log10((Donor CFU/Recipient CFU)endpoint / (Donor CFU/Recipient CFU)beginning). Perhaps just clarify in the methods.

Yes, the y-axis is log base 10. Therefore, there are 100-fold more donor cells at the end of the competition experiment when the recipient lacks *rhsA-rhsI* compared to the control. We have amended the methods as per the reviewer’s suggestion.

2) Figure 2G, RhsA∆N: I could not find a exact definition of this construct. Based on the main text line 169, I’m assuming it is an N-terminal deletion of RhsA at AA305, but I wound define this either in the legend or in the main text.

This construct lacks the first 297 amino acids of RhsA and we now indicate this in the figure legend as suggested. We designed this truncation to mimic RhsA lacking its cleaved N-terminal domain. It does not perfectly align with the H304-P305 cleavage site because this plasmid was generated prior to our determination of the cryo-EM structure. However, in our revised manuscript, we have included new mutagenesis data confirming the site of N-terminal cleavage (new Fig. S5B).

3) Supplementary Figure 3C: Should residue 313 be a K not an A?

Indeed, residue 313 is a lysine. We have corrected the figure in the revised manuscript.

4) Supplementary Figure 3H: There is also impaired cleavage of the H530A construct, but this was not mentioned in the text. This residue is highlighted in Supplementary Figure 3G, and thus seems like an important residue to comment on. This construct also appears to have a majority of -CT bands, not +CT bands, where the D318A/D319A/D320A construct has a mixture of both. Is there a possible relation of this N-terminal region to C-terminal cleavage? This may be an instance where further experiments are outside the scope of this manuscript, but I think it may be worth a comment.

We thank the reviewer for their detailed thoughts on the N-terminal cleavage site mutants. We highlighted residue H530 because it is conserved across class I prePAAR Rhs effectors.

As in the case of the triple mutant (D318-D320 to alanine), we believe that the minimally impaired cleavage is due to a possible structural rearrangement close to the cleavage site. We have added a sentence in the revised manuscript (l. 209).

It is an interesting thought that the N- and C-terminal cleavage are possibly coupled. Since both cleavage sites reside inside the cocoon, the cleavage events could indeed influence each other. To rule out that the catalytic center of the C-terminal autoprotease has an influence on N-terminal cleavage we created a construct lacking the C-terminal residues 1324 – 1486, which includes both of the C-terminal aspartyl protease catalytic residues. This construct was not deficient in N-terminal cleavage, indicating that there is no direct correlation between the proteolytic events. We have included this result in the revised manuscript (see new supplemental Figure 5I).

5) Sentences from line 333 to 338. I think this appears to be the most plausible scenario. What is not hypothesized here is how the N-terminal TM helices, which have been cleaved from the Rhs shell, may subsequently interact with the C-terminal toxin as it leaves the Rhs shell through the opened seal. Would you propose that the Shell and TM helices come together to funnel the toxin across the inner membrane, or do you see release of the toxin from the Rhs shell as a separate event from membrane translocation (i.e. the toxin has to “find” the TM helices to translocate across the inner membrane). I understand this section is a proposed model, but it may be strengthened by a bit more explanation.

We thank the reviewer for these interesting thoughts. We think as well that the most plausible scenario is that the Rhs barrel resides in the periplasm and the transmembrane helices insert into the inner cell membrane. Our proposed model shares similarity with the most recently proposed model for the translocation of the diphtheria toxin from *Corynebacterium diphtheriae* (Murphy 2011, *Toxins*). Two α-helical hairpins are proposed to form a translocation channel in the endosomal membrane through which the toxin can enter the cytosol. The potential of the diphtheria toxin to refold in the target cell cytosol is thought drive the translocation through the loosened-up membrane structure created by the two transmembrane helices. Moreover, in the T6SS field, it was shown by Pei et al. (2020 Nature Communications) that VIRN & VIRC (analogs to the cleaved N-terminal domain of RhsA and the cleaved C-terminal toxin) interact directly with each other even in the absence of the Rhs shell. Hence, it seems possible in our scenario that once the seal is opened from RhsA the toxin will be tugged along and threaded out of the barrel at the same time. We have added this paragraph to the revised manuscript.

**Reviewer #2 (remarks to the authors):**

**Major**

1) The mechanism of C-terminal portion cleavage appears to be similar to the process described for the Tc toxins and previously characterised representatives of this family. The authors suggest that the C-terminal portion cleavage can be abrogated via mutations of conserved catalytic residues, that are validated by the competition assay (Fig 2C). However, the overexpression of the same mutants in the assay presented at Supp Fig 3E suggests that these mutants retain some (less than the wild type) toxicity. Why isn’t this toxicity manifesting in the competition assay?

Indeed, even though both catalytic aspartates were individually mutated some toxicity remains. However, we overexpressed the mutants at an artificially high level in *E. coli* and therefore this observed toxicity is likely due to overexpression, which could include misfolded RhsA subpopulations whereby the structurally complex Rhs cage does not fold properly but the comparatively simple single domain toxin does. In support of this, we only obtain milligram quantities of folded, soluble RhsA for our structural work when we express it at 18 degrees C but not 37 degrees C. Based on our previously published work, we also know that the abundance of RhsA in *P. protegens* is extremely low, even compared to other T6SS effectors (Ahmad et al, eLife, 2020). To simply detect RhsA in *P. protegens* by western blotting, we had to fuse it to a His-tag and affinity purify it from a 100mL culture. This low abundance is consistent with the prevailing model of T6SS in which only a single PAAR-containing effector (i.e. RhsA) is delivered into recipient cells per T6SS firing event. To mitigate the reviewer’s concern, we have included new overexpression data in *E. coli* using lower concentrations of inducer (0.01% and 0.05% (w/v) rhamnose) to better mimic the low expression levels observed in *P. protegens* (though we expect that these RhsA levels still exceed their native levels in *P. protegens*). Consistent with this, we find that at lower expression levels in *E. coli*, the residual toxicity of the C-terminal cleavage mutants is substantially reduced (new Figure S3B).

The biochemical activity of C-terminal toxin is only implied via sequence similarity but never tested. Is the activity of C-terminal domain inhibited by the appropriate immunity protein? The presence of immunity protein often helps stabilizing and obtaining the structure of toxic domain. Was this attempted?

We thank the reviewer for raising this point. Our initial characterization of the C-terminal toxin domain of RhsA and its cognate immunity protein, RhsI, was first reported in Tang JY et al, 2018, JBC. Specifically, in Figure 1C we show the immunity function of RhsI in *E. coli*. Furthermore, in Ahmad et al, 2020, eLife we demonstrate RhsI’s immunity function in *P. protegens* during interbacterial competition. In the current manuscript, the immunity function of RhsI is shown in the *E. coli* toxicity assays shown in Supplemental Figure S3.

With regards to obtaining a structure of RhsA’s toxin domain, in Figure S1 of our JBC we found that the C-terminal domain of RhsA in isolation is so toxic that it could only be cloned when it contained a D1404A point mutation, which is a predicted catalytic residue based on the endonuclease alignment shown in Figure S2A from the same paper. This mutation alleviates some of the toxicity issues with overexpressing this toxin, but it also makes it difficult to characterize its biochemical activity because presumably this residue is important for toxin function.

We have also tried to obtain a structure of RhsA’s toxin domain in complex with RhsI but have encountered a number of technical challenges including the extreme potency of the wild-type toxin domain alluded to above (even when in complex with RhsI), the observation that this toxin-immunity complex co-purifies with DNA, and other issues. We did not encounter these technical problems with full-length RhsA, probably because the Rhs cocoon reduces the amount of ‘free’ toxin domain in the bacterial cytoplasm. Therefore, for this particular effector, it has been much easier working with the full-length protein than it has been with the isolated toxin domain. Overcoming these technical challenges will be the focus of future investigations.

2) Presented effort for gaining insight into RhsA’s N-terminal portion’s cleavage is even less convincing. The specific position of the cleavage is determined based on the absence of interpretable density after residue 305, which is not particularly accurate and can also be due to lability of this portion of the structure. There are specific mass spectrometry based assays that should be used to identify/confirm the specific position of N-terminal cleavage. Wrongly estimated cleavage site could be one of possible reasons for failing to identify the catalytic residues responsible for auto-proteolysis of N-terminal portion. Obtaining non cleavable version of RhsA could have provided insights into this mechanism. Since the mutation of “usual suspects” did not reveal the culprits more unbiased approach (alanine scanning mutagenesis?) must be applied.

As the reviewer nicely summarizes, we tried to mutate residues potentially involved in catalyzing the N-terminal cleavage without success. Based on the work of Pei et al. 2020 and based on sequence alignments we are quite confident, that the cleavage site is between residues H304/P305. However, to better substantiate this conclusion, we performed two additional experiments as suggested by the reviewer: *de novo* protein sequencing via LC-MS/MS of the cleaved Rhs-cage and generating a cleavage-resistant mutant. Both experiments confirmed that the cleavage site is indeed between residues H304/P305. We have included these new results in the revised manuscript (see new Figs S5A and S5B).

3) The model proposed for conformational changes of RhsA upon delivery by T6SS into recipient cell (Fig. 6) is by far the weakest and most over-speculative part of this study. Primarily based on experimentally unconfirmed position of the cleavage site it liberally uses analogy with Tc toxin while continuously highlighting the significant differences between these proteins and RhsA. Since no structural or functional data is provided in support of this model, I see no way of assessing its merit. Just to provide a specific example, provided model suggests that the 2 predicted transmembrane helices will facilitate the threading and/or translocation of either toxin or the entire cocoon/toxin portion through the inner membrane into recipient cell’s cytoplasm. Would authors provide an example of a translocation channel composed with only 2TMs that their model is based on? Any experimental assays tried to show the formation of such translocation channel by these 2 TMs?

We thank the reviewer for the detailed criticism of our proposed model. As described above, we performed now additional experiments that show that indeed both the N- and C-terminal cleavage sites are at the positions we have initially described in the manuscript.

Concerning the threading of the toxin through the inner cell membrane, we propose that the two transmembrane helices could form a very small translocation pore. Most likely, this pore is too small to accommodate the folded toxin and is thus thought to translocate the toxin into the cytosol in an unfolded state. Our model shares similarity with the current model proposed for toxin translocation of the *Corynebacterium diphtheriae* diphtheria toxin. In this case, two α-helical hairpins insert into the endosomal membrane to form a translocation channel through which the toxin is threaded into the cytosol of the attacked cell (Murphy 2011, *Toxins*). We have added this comparison to the revised manuscript.

**Minor**

1) Given the important similarity between RhsA and Tc toxins/ teneurin discussed throughout the manuscript more information about these proteins must be provided in the introduction.

We thank the reviewer for raising this point. We have added more information on Tc toxins and teneurins to the introduction.

2) L-62-63: “latter likely plays a role in target in cell penetration”. In the context of this sentence “latter” refers to transmembrane helices while the cell penetration is rather function of VgrG spike, no? Consider rewording.

We rephrased the sentence and it should be now clear which element contributes to which function.

3) L-74: “Tc toxin”. – As per my general comment above the Tc toxin needs proper introduction/explanation prior to this statement.

See point 1) above. We have added more explanations in the introduction.

4) L-134: Supp Fig 3C-E. Should be corrected to Supp Fig 3B.

This has been corrected.

5) L-151: Is the functional prediction based on structural similarity or primary sequence features?

The functional prediction is based on both, sequence similarity to other predicted nucleases (see Figure S2 in Tang JY et al., 2018, JBC) and based on biochemical activity of characterized homologous proteins (Koskiniemi et al. 2013 *PNAS*). We have modified the sentence in the revised version of the manuscript.

6) L-246-249: “hyperconserved region”. I am not sure there is such a term. Earlier in introduction, C-terminal part of Rhs is mentioned as “hypervariable region”, but within this domain there is hyperconserved region?

We agree with the reviewer that the term “hyperconserved” causes confusion. We used now a different wording in the manuscript.

7) Fig 3C: Is it teneurin structure presented here? If so, please add the proper statement to the legend as the title for Fig 3 refers to RhsA structure. From main text, it appears to be the teneurin structure.

We agree with the reviewer that it is not directly evident to which structure the helix belongs in Figure 3C. This helix is the anchor helix of RhsA. We modified slightly the figure and figure legend to clarify the raised point.

8) L-309: “After cleavage…” Is it after cleavage that EagR1 binds to N-terminal TM or is it before? From Fig6, it looks like EagR1 binds before the cleavage.

We thank the reviewer for pointing this out. We corrected the sentence in the manuscript accordingly.

9) L-327: “As proposed previously…” Appropriate reference is needed.

We have added the corresponding reference to the manuscript.

**Reviewer #3 (remarks to the authors):**

1) The observed dimerisation of RhsA is striking on two counts. The head-to-tail dimers are initially striking for their similarity to dimers of the eukaryotic RHS-repeat containing protein Teneurin. It is also striking that this arrangement seems non-productive in the context of the T6SS. This is an issue that the authors should address. Presumably the dimer is an artefact of the isolated VgrG1-RhsA complex? Is it possible to rationalise the structure of the VgrG1-RhsA complex seen here with the T6SS spike structure reported by Nazarov et al. ([doi.org/10.15252/embj.201797103](http://doi.org/10.15252/embj.201797103))?

As this reviewer mentions, the formation of head-to-tail dimers would potentially require further investigation to identify a potential physiological role of this complex. In comparison to the dimers formed between teneurins, dimerization of RhsA occurs via a different interface. While in teneurins, the EGF-like domains induce dimerization by disulphide bridge formation, the RhsA dimer is established via direct interactions of the Rhs repeats. We agree with the reviewer that this arrangement would certainly seem to be counterproductive in the context of what is known about T6SS spike ‘firing’ given that the proper orientation of the VgrG spike is likely crucial for outer membrane penetration of the target cell. Based on the data presented in the referenced Nazarov paper, the authors of this study speculate that the cavity around the VgrG-PAAR spike complex could accommodate up to three ~150kDa proteins. This makes sense in the context of effectors such as Tse1 that interact with VgrG in a 3:3 ratio (see Flaugnatti et al, EMBO, 2020). However, given the established 3:1 stoichiometry between VgrG proteins and PAAR domains (such as the PAAR domain present in RhsA’s N-terminus), we favour a model in which a single RhsA molecule interacts with its cognate homotrimeric VgrG1 spike.

2) Although it builds on previously published work, the delivery model suggested in Figure 6 is the most speculative part of the paper, and does not seem fully supported by the available data. It is unclear how two trans-membrane helices will be sufficient to form a pore able to translocate a polypeptide through the inner membrane. Is it possible that inner membrane proteins in the target cell are also required? It is additionally unclear how the T6SS contractile event does not simply push the sharp PAAR tip through the inner membrane. Not all PAAR-containing effectors contain a TM-helix-containing pre-PAAR domain, which implies at least the existence of an alternate mechanism.

We thank the reviewer for the well-justified criticism on our proposed model of RhsA delivery into target cells. We also agree that potential other routes exist which are not depicted in the model figure. However, based on previous work we could show *in vitro* that effectors loaded onto VgrG can spontaneously translocate liposomal membranes by a yet unknown mechanism (Quentin et al, Nature Microbiology, 2018). Therefore, direct translocation of the entire RhsA effector across the inner membrane of the target cell is one potential route. If the barrel is not translocated completely and would remain inside the periplasm, one potential mechanism would be that the transmembrane helices aid the translocation of the toxin into the cytosol. Our model shares similarity with the current model proposed for the translocation of the diphtheria toxin from *Corynebacterium diphtheriae* (Murphy 2011, *Toxins*). In this case, two α-helical hairpins insert into the endosomal membrane to form a translocation channel through which the toxin is threaded into the cytosol of the attacked cell.

As the reviewer points out, there are multiple other potential routes for delivery of the toxin into the cytosol. This potentially involves other membrane proteins and periplasmic proteins that could facilitate toxin translocation and would therefore be part of the toxin delivery route. We have added respective paragraphs to the revised manuscript. It is worth noting, however, that in contrast to receptor-mediated toxin delivery systems in bacteria (bacteriophages, colicins, contact-dependent inhibition, etc.), T6SS-mediated bacterial killing is broad spectrum (see Schwarz et al, 2010, Plos Pathogens and Chatzidaki-Livanis, 2016, PNAS). In our opinion, this makes a receptor-mediated translocation model less likely, though it is of course still a formal possibility.

**Major**

1) The autoproteolysis of the N-terminal domain is a significant new finding. Frustratingly, mutation of the identified possible active site residues for the protease activity (e.g. C538) does not alter protease activity. It is therefore suggested that the encapsulated toxin contributes active site residues for the protease activity. Given that the C-terminal toxin domain is unfolded within the cocoon, it seems unlikely that it can contribute active site residues to the proteolytic cleavage. However, this idea is testable: Is the N-terminal domain cleaved if a truncated protein lacking the C-terminal toxin is expressed? This is a relatively straightforward experiment that should be performed.

We thank the reviewer for suggesting this straightforward experiment. Following their advice, we generated a mutant that lacks the C-terminal toxin and checked if N-terminal cleavage still occurs. The new results show that N-terminal cleavage is not abolished if the C-terminal toxin domain is removed, demonstrating that the C-terminal toxin is not involved in N-terminal cleavage. We have included the new results and rephrased the according paragraph in the revised manuscript (see new supplemental Figure 5I).

**Minor**

1) Line 79: It’s not clear what is meant by “host cell targeting”.

We used a different wording to clarify the raised point.

2) Line 93 and elsewhere: RHS repeats do not form a β-barrel, which is a specific defined protein topology where β-sheets form a closed toroid distinguished by continuous hydrogen bonds between strands. In contrast RHS repeats form an extended β-sheet which is folded around onto itself. This is best described as a “cocoon “or a “shell” instead.

We thank the reviewer for bringing this point up and agree with it. We used proper phrasing in the rest of the manuscript.

3) Line 168-169 -ΔN and ΔNT is somewhat confusing terminology.

We apologize for causing confusion. We renamed the construct ∆NT to “∆TMD” to more intuitively indicate that it only lacks the N-terminal transmembrane domains whereas ∆N refers to the construct in which the entire cleaved N-terminal region has been omitted. We hope this clarifies the constructs used in our study. This new designation has been updated in the manuscript and figures accordingly.

4) Line 226: The phrase “Rhs core” has been used previously in the published literature to refer to hyperconserved autoprotease region. Here the cocoon is referred to as the “core”, which is potentially confusing.

We thank the reviewer for the comment. We changed throughout the manuscript “Rhs core” either to “Rhs repeats” or “Rhs barrel” which is more consistent.

5) Lines 752-756 Should the PDB and EMDB IDs be listed?

At the point we submitted the manuscript, the experimental data was still under curation for both databases. Now, the data was accepted for both databases and we have added the respective IDs into the manuscript.
